# Single-Cell Dissection Identifies METTL7B as Associated with Cell Adhesion-Mediated Tumor Invasion in Lung Adenocarcinoma and Glioblastoma

**DOI:** 10.3390/cancers18091384

**Published:** 2026-04-27

**Authors:** Jie Mao, Jinquan Xia, Huibin Song, Fuhua Zhong, Huiyi Feng, Junhui Chen, Guangsuo Wang

**Affiliations:** 1 Department of Neurosurgery, Longgang Central Hospital of Shenzhen, Shenzhen 518116, China; 2 Shenzhen People’s Hospital (The First Affiliated Hospital, Southern University of Science and Technology; The Second Clinical Medical College, Jinan University), Shenzhen 518020, China; 3 Shenzhen Clinical Medical College, Guangzhou University of Chinese Medicine, Shenzhen 518116, China; 4 Department of Central Laboratory, Shenzhen People’s Hospital (The First Affiliated Hospital, Southern University of Science and Technology; The Second Clinical Medical College, Jinan University), Shenzhen 518020, China; 5 Department of Thoracic Surgery, Shenzhen People’s Hospital (The First Affiliated Hospital, Southern University of Science and Technology; The Second Clinical Medical College, Jinan University), Shenzhen 518020, China; 6 Department of Oncology, Shenzhen Hospital of Southern Medical University, Shenzhen 518100, China; 7 Intervention and Cell Therapy Center, Peking University Shenzhen Hospital, Shenzhen 518036, China; 8 Department of Minimally Invasive Intervention, Peking University Shenzhen Hospital, Shenzhen 518036, China

**Keywords:** METTL7B, cell adhesion signaling, cancer invasion and migration, single-cell RNA sequencing, ITGA3

## Abstract

**Background**: Tumor invasion is the central barrier to effective immunotherapy in lung adenocarcinoma (LUAD) and glioblastoma. Cell adhesion signaling critically shapes tumor–microenvironment interactions, yet the upstream regulators coordinating these invasive programs at single-cell resolution remain incompletely understood. Methyltransferase Like protein 7B (METTL7B) has recently emerged as a candidate oncogenic regulator, but its lineage-specific functions and the potential downstream effectors are unclear. **Methods**: We integrated publicly available single-cell RNA sequencing datasets from LUAD and glioblastoma with The Cancer Genome Atlas (TCGA) transcriptomic analyses to resolve *METTL7B*-associated malignant cell states and microenvironmental interactions. Functional enrichment analyses identified invasion- and focal adhesion pathways linked to *METTL7B* expression. Gain- and loss-of-function experiments were conducted in LUAD and glioblastoma cell lines to validate downstream cell adhesion effectors. Spatial expression patterns were examined using immunofluorescence, and transwell assays were used to assess migratory and invasive phenotypes. **Results**: Single-cell analyses revealed that *METTL7B* was selectively enriched in malignant epithelial cells in LUAD and glioblastoma and defined a transcriptional program characterized by cell adhesion signaling. Integrin Alpha 3 (*ITGA3*) emerged as a conserved downstream effector of *METTL7B*, with progressive upregulation from minimally invasive to invasive LUAD and glioblastoma. Functional perturbation confirmed that METTL7B enhances tumor cell migration and invasion through integrin-associated pathways. **Conclusions**: METTL7B acts as a potential lineage-enriched regulator of invasive tumor states by activating cell adhesion signaling in LUAD and glioblastoma. These findings position METTL7B as a putative prognostic factor for strategies aimed at limiting invasion in lung cancer and glioblastoma.

## Simple Summary

Tumor invasion limits the effectiveness of immunotherapy in lung adenocarcinoma and glioblastoma. This study shows that METTL7B promotes cancer cell invasion by activating cell adhesion signaling. Using single-cell analysis datasets and functional experiments, we identified ITGA3 as a key downstream effector of METTL7B. METTL7B enhances tumor cell migration and invasion through cell adhesion-associated pathways. These findings suggest that METTL7B may serve as a potential prognostic factor for limiting invasion in these aggressive cancers.

## 1. Introduction

Lung cancer and gliomas are among the most lethal malignancies worldwide and most of the diagnoses have already progressed to the middle to late stages, posing major clinical and societal challenges [1]. In lung cancer, LUAD represented the most common histological subtype of non-small cell lung cancer (NSCLC), while glioblastoma represented the most common histological subtype of gliomas [2,3,4]. In addition to surgery, conventional radiotherapy and chemotherapy, immunotherapy including PD-1 antibodies, CAR-T-cell therapy, tumor neoantigen vaccines, and oncolytic viruses have shown encouraging prospects in the treatment of LUAD and glioblastoma [5]. However, both LUAD and glioblastoma are marked by pronounced intratumoral heterogeneity and dynamic tumor–microenvironment interactions, which complicate disease management and limit durable therapeutic responses [6,7]. Understanding the cellular heterogeneity and molecular mediators of LUAD and glioblastoma has been a longstanding challenge. Traditional bulk transcriptomic and genomic approaches have provided important insights into recurrent mutations, oncogenic signaling pathways, and immune evasion mechanisms [8,9]. However, bulk analyses average signals across diverse cell types, obscuring lineage-specific programs and preventing the resolution of rare subpopulations that may drive tumor initiation, progression, and resistance. Recent advances in single-cell RNA sequencing (scRNA-seq) have revolutionized cancer biology by enabling dissection of complex tumor ecosystems at single-cell resolution [10]. ScRNA-seq studies in LUAD have uncovered malignant epithelial subclones with distinct proliferative or metastatic programs, as well as diverse immune infiltrates including exhausted T cells, immunosuppressive myeloid populations, and fibroblasts contributing to stromal remodeling [11,12]. In gliomas, scRNA-seq has revealed a spectrum of malignant glial states, including neural progenitor-like, oligodendrocyte-like, and astrocyte-like phenotypes, along with tumor-associated macrophages and microglia that foster immunosuppression and angiogenesis [13,14]. These findings highlight the value of scRNA-seq in elucidating tumor heterogeneity and identifying lineage-specific molecular mediators. Among the emerging molecules of interest in cancer biology is METTL7B, a member of the Methyltransferase Like (METTL) family of putative methyltransferases [15]. Unlike well-studied RNA methyltransferases such as Methyltransferase-like protein 3 (METTL3) and Methyltransferase-like protein 14 (METTL14), which regulate m^6^A RNA modifications, the biological functions of METTL7B are far less characterized [15]. METTL7B has been implicated in lipid droplet biology, endoplasmic reticulum function, and redox homeostasis, suggesting potential roles in cellular stress responses and metabolism [16]. Recently, we and other researchers have hinted at possible oncogenic functions of METTL7B. For instance, aberrant METTL7B expression has been observed in certain malignancies such as thyroid, lung and breast cancers, and its upregulation has been related to enhanced tumor proliferation, Tyrosine Kinase Inhibitor (TKI) resistance and poor prognosis in some cohorts [16,17,18,19]. However, its cell type-specific expression patterns, mechanistic contributions to tumor progression, and clinical relevance in diverse cancers remain poorly understood.

There is a critical need to identify molecular regulators that drive invasive phenotypes across distinct cancer lineages. In this study, we investigated *METTL7B* as a previously underexplored regulator of tumor invasiveness by integrating single-cell transcriptomic profiling of LUAD and glioblastoma with functional and spatial validation. We specifically sought to define the cellular sources of *METTL7B* within malignant ecosystems, elucidate its downstream engagement of cell adhesion signaling pathways, and determine its contribution to tumor cell migration and invasion. By linking METTL7B to lineage-enriched invasive programs in lung and brain tumors, this work aims to establish METTL7B as a putative prognostic factor for limiting tumor progression and invasion.

## 2. Materials and Methods

### 2.1. Patient Samples and Ethical Approval

Primary tumor tissues and matched adjacent non-tumor tissues were collected from patients with LUAD and glioblastoma who underwent surgical resection at Shenzhen People’s Hospital between January 2023 and August 2025. The cohort included three paired samples each of minimally invasive LUAD, invasive LUAD, low-grade glioblastoma, and high-grade glioblastoma, together with corresponding adjacent tissues. Histopathological classification and grading were independently confirmed by two certified pathologists according to World Health Organization (WHO) criteria. All patients provided written informed consent prior to sample collection. The study protocol was reviewed and approved by the Institutional Ethics Committee of Shenzhen People’s Hospital (approval numbers: LL-KY-2022245 and LL-KY-2024161) and was conducted in accordance with the Declaration of Helsinki.

### 2.2. Cell Lines and Culture Conditions

The human NSCLC cell line A549 (lung adenocarcinoma cell, mutation: KRAS G12S and STK11 Q37X) was cultured in RPMI-1640 medium (Sigma-Aldrich, St. Louis, MO, USA), and the human glioma cell line U251 (glioblastoma cell, mutation: TP53 A273H, TERT C228T and PTEN E242Vfs*15) was cultured in improved DMEM (CellCook, Guangzhou, China). Both cell lines were obtained from CellCook. Both media were supplemented with 10% fetal bovine serum (Gibco, Waltham, MA, USA) and 1% penicillin-streptomycin (Gibco). Both cells were maintained at 37 °C in a humidified incubator containing 5% CO_2_ and routinely tested to confirm the absence of mycoplasma contamination. The cell lines were authenticated by short tandem repeat (STR) profiling (Supplementary Files S1 and S2).

### 2.3. Lentiviral Production and Knockout of METTL7B

The pLKO.1-GFP control vector were used for gene knockdown experiments. Lentivirus packaging, concentration, and transduction were performed as previously described [19]. For gene knockout, a single-guide RNA (sgRNA) targeting human METTL7B (5′-GTTCTACCCACCGGGCTGCA-3′) was synthesized and cloned into the pLV-U6-puro vector. Lentiviral particles were generated using standard packaging plasmids and used to infect LUAD and glioblastoma cells, followed by puromycin selection to establish stable knockout cell lines. Knockdown and knockout efficiencies were confirmed by qRT-PCR and Western blotting prior to downstream functional assays.

### 2.4. Single-Cell RNA Sequencing Data Acquisition and Processing

Publicly available scRNA-seq datasets were analyzed to delineate METTL7B-associated malignant states and tumor microenvironmental interactions. LUAD datasets were obtained from ArrayExpress (E-MTAB-6149 and E-MTAB-6653; 8 samples) and Gene Expression Omnibus (GEO) (GSE123902; 12 samples), while glioblastoma data were downloaded from GEO (GSE273274) [20]. Raw data were processed using Seurat (v4.1.0). Quality control filters excluded cells with fewer than 300 detected genes, mitochondrial gene content exceeding 20% for LUAD or 10% for glioma, and genes expressed in fewer than three cells. Doublets were identified and removed using DoubletFinder (v2.0.3). Batch effects across datasets were corrected using Harmony (v0.1.0). Cell clustering and annotation were performed using Seurat (v4.1.0), guided by canonical marker genes, published annotations, and the CellMarker database. Differential gene expression analysis was conducted using the Seurat FindMarkers function (min.pct = 0.1, logfc.threshold = 0.25, adjusted *p* < 0.05). Functional enrichment analyses of METTL7B-associated gene signatures were performed using clusterProfiler (v3.18.1), focusing on Gene Ontology (GO) biological processes and Kyoto Encyclopedia of Genes and Genomes (KEGG) pathways related to cell–substrate adhesion, focal adhesion, and tumor invasion.

### 2.5. TCGA and Gene Expression Profiling Interactive Analysis 2 (GEPIA2) Expression Analysis

The expression patterns of *METTL7B* in LUAD and glioblastoma were further evaluated using RNA-seq data from TCGA via the GEPIA2 web platform [21,22]. Tumor samples were compared with corresponding normal tissues to assess differential expression and clinical relevance.

### 2.6. Immunofluorescence Staining

Paraffin-embedded tissue sections from patients with LUAD and glioblastoma were subjected to immunofluorescence staining. Sections were first deparaffinized by incubation at room temperature for 10 min, followed by immersion in xylene (3 min), 100% ethanol (5 min), 85% ethanol (5 min), and 75% ethanol (5 min), and rinsed in double-distilled water (5 min). Followed antigen retrieval, sections were washed three times in PBS (5 min each) on a shaker. After blocking with 3% bovine serum albumin (BSA) for 30 min, sections were incubated overnight at 4 °C in a humidified chamber with primary antibodies against METTL7B (Proteintech, 17001-1-AP) and ITGA3 (Proteintech, 66070-1-Ig). The following day, slides were washed three times with PBS (5 min each) and incubated for 1 h at room temperature in the dark with appropriate fluorophore-conjugated secondary antibodies. Nuclei were counterstained with DAPI for 15 min, and slides were mounted with an anti-fade reagent. Images were acquired using a fluorescence microscope and analyzed with ImageJ software (version 1.8.0).

### 2.7. Quantitative Real-Time PCR (qRT-PCR)

Total RNA from LUAD and glioblastoma cells was extracted with Trizol and reverse-transcribed to cDNA (25 °C 5 min, 42 °C 30 min, 85 °C 5 s). qPCR was performed with PerfectStart Green SuperMix using a two-step protocol (94 °C 30 s; 94 °C 5 s, 60 °C 30 s × 40 cycles). Relative expression was calculated using the 2^−ΔΔCt^ method, normalized to GAPDH. Primer sequences: ITGA3: F 5′-CAGATGGATGTGGATGAGAA-3′; R 5′-TTGTGGACGATGTTGATGA-3′; METTL7B: F 5′-CCAGATAAAGGGGCTTACAGGAG-3′; R 5′-TCAGCCATGCTCTTTGTCAGG-3′; GAPDH: F 5′-GTCTCCTCTGACTTCAACAGCG-3′; R 5′-ACCACCCTGTTGCTGTAGCCAA-3′.

### 2.8. Western Blot

Total protein was extracted from METTL7B-overexpressing, METTL7B-knockout, and corresponding control LUAD and glioblastoma cells. Protein concentrations were determined using a BCA assay kit. Equal amounts of protein (50 μg per lane) were separated by Sodium Dodecyl Sulfate-Polyacrylamide Gel Electrophoresis (SDS–PAGE) and transferred onto polyvinylidene difluoride (PVDF) membranes. Membranes were blocked with 5% skim milk at room temperature for 1 h and incubated overnight at 4 °C with primary antibodies against METTL7B, ITGA3, and GAPDH. After washing, membranes were incubated with Horseradish Peroxidase (HRP)-conjugated secondary antibodies at room temperature for 1 h. Protein bands were visualized using Enhanced Chemiluminescence (ECL) detection system.

### 2.9. Migration and Invasion Assays

Migration assays were performed using uncoated Transwell chambers (8.0 μm pore size). LUAD and glioblastoma cells (3 × 10^4^/well) in serum-free medium were seeded into the upper chambers, with 10% FBS medium in the lower chambers. After 24 h, migrated cells were fixed with paraformaldehyde, stained with 0.1% crystal violet, washed, and counted microscopically. Invasion assays were performed using Matrigel-coated Transwell chambers. After rehydration, 3 × 10^4^ cells were seeded into the upper chambers and incubated for 24 h. Cells that invaded through the Matrigel were fixed, stained, and counted as above.

### 2.10. Statistical Analysis

Data are presented as mean ± SD. Statistical differences between groups were assessed by unpaired Student’s *t*-test or one-way ANOVA followed by Bonferroni’s multiple comparison tests using GraphPad Prism 10.0. A *p* value < 0.05 was considered statistically significant.

## 3. Results

### 3.1. Single-Cell Transcriptomic Profiling of Lung Adenocarcinoma and Glioblastoma

**Figure 1:**
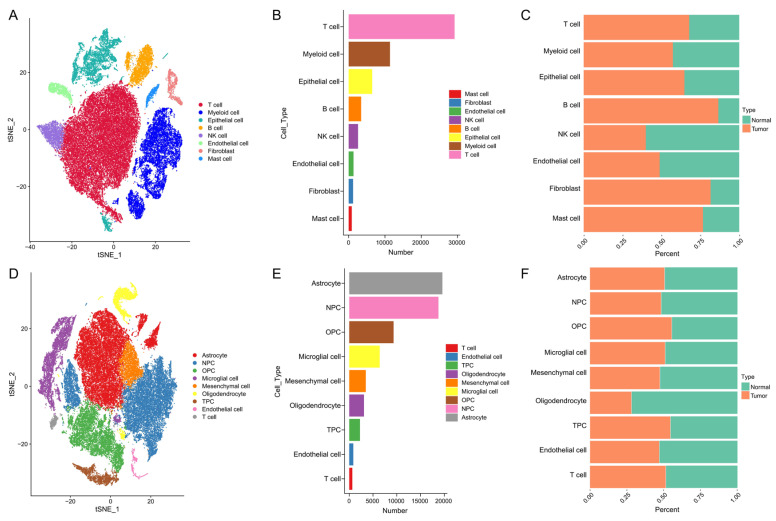
Single-cell transcriptomic landscapes of lung adenocarcinoma and glioblastoma. (**A**–**C**) t-SNE visualization of lung adenocarcinoma single-cell transcriptomes showing distinct clusters by cell type (**A**), cell number distribution across clusters (**B**), and proportions of tumor versus adjacent non-tumor samples within each cell type (**C**). (**D**–**F**) t-SNE visualization of glioma single-cell transcriptomes showing distinct clusters by cell type (**D**), cell number distribution across clusters (**E**), and proportions of tumor versus adjacent non-tumor samples within each cell type (**F**).

Given the similar tumor heterogeneity between LUAD and glioblastoma, we conducted analysis in scRNA-seq datasets on tumor and matched adjacent non-tumor tissues from publicly available scRNA-seq datasets. In LUAD, t-SNE visualization revealed clear clustering of single cells into multiple populations corresponding to T cell, myeloid cell, epithelial cell, B cell, NK cell, endothelial cell, fibroblast and mast cell (Figure 1A). Quantitative assessment showed variable cell numbers across clusters, with T-cell, myeloid and epithelial cell being predominant (Figure 1B). Comparison of sample origins demonstrated distinct shifts in cellular composition between tumor and adjacent tissues, with tumor samples enriched in T-cell, myeloid cell, Epitheial cell, B-cell, fibroblast and mast cell (Figure 1C). In glioblastoma, t-SNE analysis similarly delineated discrete clusters corresponding to Astrocyte, Neural Progenitor Cells (NPC), Oligodendrocyte Progenitor Cells (OPCs), microglial cells, mesenchymal cells, oliodendrocyte, Transition Progenitor Cells (TPCs), endothelial cells and T cells (Figure 1D). The distribution of cell numbers across these clusters highlighted the dominance of Astrocyte, NPC, OPC, microglial cell (Figure 1E). Analysis of tumor versus adjacent samples further revealed that the overall proportions of most cell types were comparable between tumor and normal tissues, with the exception of oligodendrocytes, which were predominantly enriched in normal samples (Figure 1F). Together, these data illustrate the distinct cellular landscapes of lung adenocarcinoma and glioblastoma, highlighting both tumor-type-specific heterogeneity and systematic differences between malignant and adjacent non-malignant tissues.

### 3.2. METTL7B Is Upregulated in Lung Adenocarcinoma and Glioblastoma and Exhibits Cell Type-Specific Enrichment

**Figure 2:**
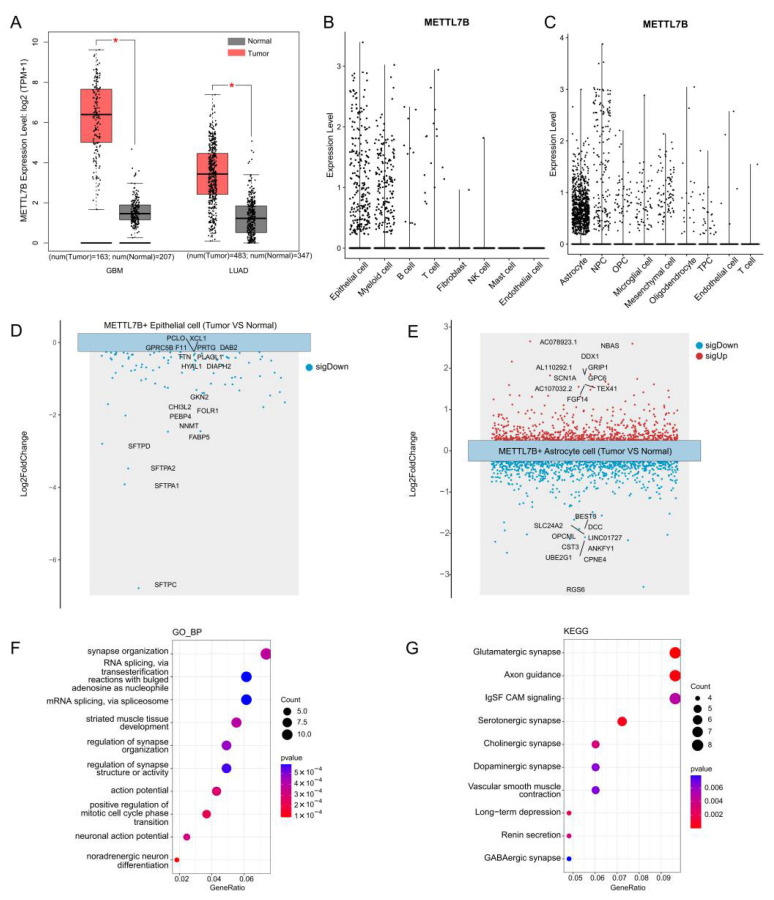
METTL7B expression and functional characterization in lung adenocarcinoma and glioblastoma. (**A**) TCGA analysis via GEPIA2 showing METTL7B upregulation in tumor versus normal tissues of lung adenocarcinoma (LUAD) and glioblastoma.* *p* < 0.05. (**B**,**C**) Single-cell t-SNE plots showing METTL7B enrichment in LUAD epithelial/myeloid cells (**B**) and glioblastoma astrocytes/neural progenitors (**C**). (**D**,**E**) Differential expression analyses of METTL7B-positive LUAD epithelial cells (**D**) and glioblastoma astrocytes (**E**) comparing tumor with adjacent tissues, highlighting the top 10 up- and downregulated genes. (**F**) GO enrichment of upregulated genes in METTL7B-positive glioblastoma astrocytes. (**G**) KEGG enrichment of upregulated genes in METTL7B-positive glioblastoma astrocytes.

We analyzed TCGA datasets using the GEPIA2 platform. *METTL7B* expression was higher in tumor tissues than in adjacent ones in both LUAD and glioblastoma (Figure 2A). In LUAD, *METTL7B* was predominantly expressed in epithelial cells, with additional enrichment in myeloid populations (Figure 2B). In glioblastoma, *METTL7B* expression was most pronounced in astrocytes, followed by neural progenitor cells (Figure 2C). These results demonstrate that *METTL7B* expression is not uniformly distributed across the tumor microenvironment but is instead concentrated within lineage-specific populations that play central roles in tumor biology.

### 3.3. Functional Differences Between METTL7B-Positive Cells in Tumor Versus Adjacent Tissues

To reveal the transcriptional programs associated with *METTL7B* expression, we compared *METTL7B*-positive cells from tumor versus adjacent tissues. In LUAD epithelial cells, tumor-derived *METTL7B*-positive cells exhibited 92 downregulated genes compared with their adjacent counterparts (adjusted *p* < 0.05) (Supplementary Table S1). The top downregulated genes included *PCLO*, *XCL1*, *GPRC5B*, *F11*, *PRTG* and *DAB2* (Figure 2D). In glioblastoma, *METTL7B*-positive astrocytes from tumor tissues demonstrated 214 upregulated and 390 downregulated genes compared with adjacent astrocytes (Supplementary Table S2). The most upregulated genes included *NBAS*, *DDX1*, *GPIP1*, *SCN1A*, *GPC6* and *FGF14*, whereas genes such as *BEST3*, *SLC24A2*, *DCC*, *OPCML*, *LINC01727*, *CST3*, *ANKFY1*, *UBE2G1*, *CPNE4*, and *RGS6* were down-regulated (Figure 2E). Among the most upregulated genes listed, FGF14, SCN1A, and GPC6 have the most significant and well-documented pro-tumor roles in glioma, supported by functional and clinical evidence [23,24,25]. Functional enrichment analysis using Gene Ontology terms indicated that upregulated genes were significantly enriched in synapse organization, RNA splicing, via transesterification reactions with bulged adenosine as nucleophile, mRNA splicing, via spliceosome, striated muscle tissue development, regulation of synapse structure or activity, action potential, positive regulation of cell cycle phase transition and neuronal action potential (Figure 2F). KEGG pathway analysis underscored enrichment of pathways linked to glutamatergic synapse, axon guidance, IgSF CAM signaling, serotonergic synapse, cholinergic synapse, dopaminergic synapse, Vascular smooth muscle contraction, long-term depression, renin secretion and GABAergic synapse (Figure 2G).

### 3.4. Functional Programs of METTL7B-Positive Tumor Cells in Lung Adenocarcinoma and Glioblastoma

**Figure 3:**
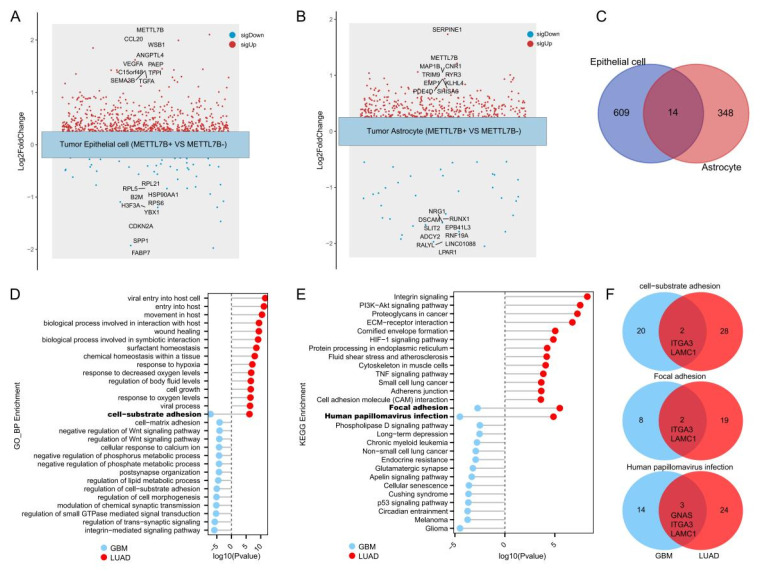
Functional characterization of METTL7B-positive tumor cells in lung adenocarcinoma and glioblastoma. (**A**) Differential expression analysis of METTL7B-positive versus negative epithelial tumor cells from LUAD, showing the top 10 upregulated and downregulated genes. (**B**) Differential expression analysis of METTL7B-positive versus negative astrocytic tumor cells from glioma, showing the top 10 upregulated and downregulated genes. (**C**) Overlap of upregulated genes between METTL7B-positive LUAD epithelial cells and glioblastoma astrocytes. (**D**) GO enrichment of upregulated genes in LUAD epithelial and glioblastoma astrocytic tumor cells, highlighting the top 10 terms and shared biological processes. (**E**) KEGG pathway enrichment of upregulated genes in LUAD epithelial and glioblastoma astrocytic tumor cells, with overlapping pathways including focal adhesion, and human papillomavirus infection. (**F**) Identification of core genes (ITGA3 and LAMC1) within the shared cell adhesion pathway for subsequent experimental validation.

To investigate the transcriptional consequences of *METTL7B* activation within tumors, we stratified malignant cells into *METTL7B*-positive and -negative groups and performed differential expression analyses. In LUAD epithelial tumor cells, 623 genes were upregulated and 44 were downregulated in the METTL7B-positive group (adjusted *p* < 0.05) (Supplementary Table S3). The most upregulated genes included *METTL7B*, *CCL20*, *ANGPTL4*, *WSB1*, *VEGFA*, *PAEP*, *TGFA* and *TFPI*, while downregulated genes included *RARRES1*, *SLPI*, *AGER*, *S100A8*, *CDKN2A*, *KRT17*, *PGC*, *SPP1* and *FABP7* (Figure 3A). Similarly, in glioblastoma astrocytic tumor cells, 362 genes were upregulated and 36 downregulated in *METTL7B*-positive cells (Supplementary Table S4), with notable increases in *METTL7B*, *SERPINE1*, *CNR1*, *MAP1B*, *TRIM9*, *KLHL4* and *EMP1* and decreases in *NRG1*, *DSCAM*, *EPB41L3*, *RNF19A*, *ADCY2*, *SLIT2*, and *LINC01088* (Figure 3B). Cross-tumor comparison uncovered both lineage-restricted and conserved transcriptional programs, including a common *METTL7B*-associated gene module comprising 23 upregulated genes shared between LUAD epithelial and glioblastoma astrocytic malignant cells (Figure 3C). Functional enrichment analysis highlighted overlapping biological processes related to tumor microenvironment remodeling, notably cell–substrate adhesion (Figure 3D). KEGG pathway analysis identified a core set of signaling pathways shared by *METTL7B*-positive cells in both LUAD and glioblastoma, including focal adhesion, and human papillomavirus (HPV) infection pathways (Figure 3E). Notably, within the common functional enrichment terms, *ITGA3* and *LAMC1* were consistently upregulated across both tumor types, supporting a conserved role for METTL7B in cell adhesion signaling programs (Figure 3F).

### 3.5. The Expression of METTL7B Is Significantly Positively Correlated with ITGA3 in LUAD and Glioblastoma Cells

**Figure 4:**
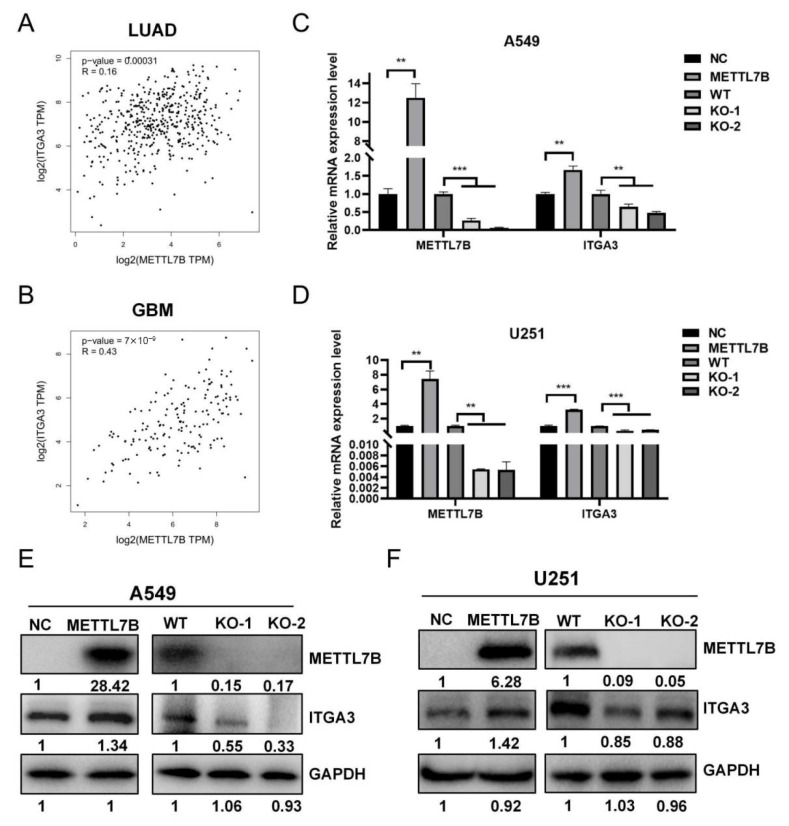
METTL7B upregulates ITGA3 expression at the mRNA and protein levels in lung adenocarcinoma and glioblastoma cells. (**A**,**B**) The correlation analysis of METTL7B and ITGA3 in LUAD and glioblastoma from GEPIA database. (**C**,**D**) qRT-PCR analysis of METTL7B and ITGA3 mRNA levels in A549 (**C**) and U251 (**D**) after METTL7B overexpression or knockout. (**E**,**F**) Western blot analyses of METTL7B and ITGA3 protein expression in A549 (**E**) and U251 (**F**) cells after METTL7B overexpression or knockout. *n* = 3; ** *p* < 0.01 and *** *p* < 0.001.

To validate the relationship between *METTL7B* and its putative downstream target *ITGA3*, we analyzed the correlation between *METTL7B* and *ITGA3* using the GEPIA database. The results showed that the expression of *METTL7B* and *ITGA3* was significantly positive correlated in tumor tissues of LUAD and glioblastoma patients (Figure 4A,B). we manipulated *METTL7B* expression in multiple LUAD and glioblastoma cell lines. In LUAD A549 and glioblastoma U251 cells, *METTL7B* overexpression induced ITGA3 expression, whereas knockout of *METTL7B* suppressed both *METTL7B* and *ITGA3* (Figure 4C,D). At the protein level, Western blot analyses corroborated these findings. METTL7B overexpression elevated METTL7B protein abundance while METTL7B knockout consistently suppressed ITGA3 protein levels in both U251 and A549 cells (Figure 4E,F). Together, these results confirm that the expression of METTL7B is significantly positively correlated with ITGA3 at both mRNA and protein levels across LUAD and glioblastoma models. These data highlight ITGA3 as a potential downstream effector of METTL7B.

### 3.6. METTL7B and ITGA3 Are Upregulated Expressed in Glioblastoma and LUAD as the Tumor Progresses

**Figure 5:**
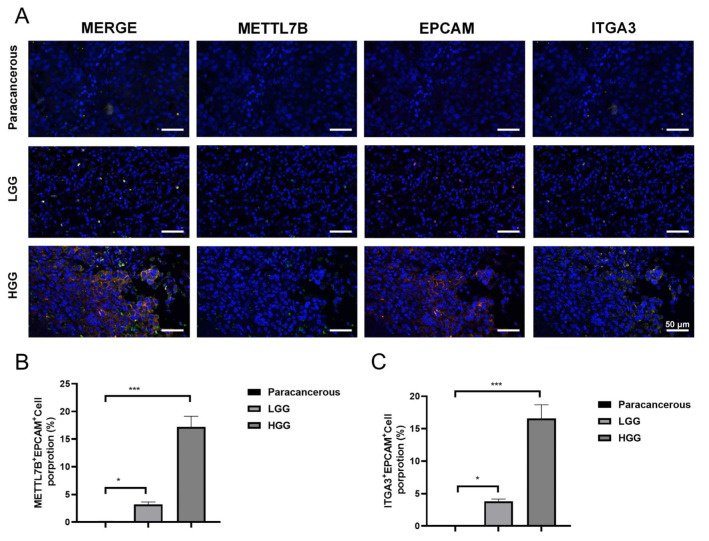
Immunofluorescent staining of METTL7B and ITGA3 in glioblastoma tissues. (**A**) Representative immunofluorescent images of METTL7B and ITGA3 in paracancerous brain tissue, low-grade glioblastoma, and high-grade glioblastoma. (**B**) Statistical analysis of METTL7B^+^EPCAM^+^cell proportion. (**C**) Statistical analysis of ITGA3^+^EPCAM^+^cell proportion. Scale bar: 50 μm. n = 3; * *p* < 0.05 and *** *p* < 0.001.

**Figure 6:**
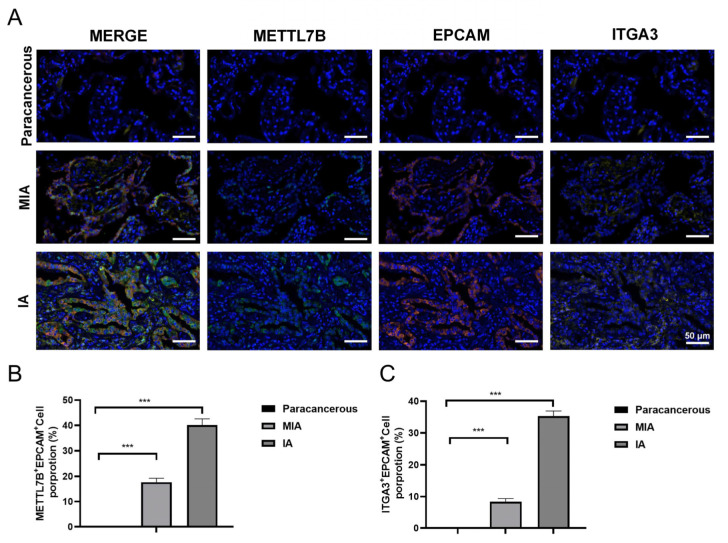
Immunofluorescent staining of METTL7B and ITGA3 in LUAD tissues. (**A**) Representative immunofluorescent images of METTL7B and ITGA3 in paracancerous lung tissue, mini-invasive and invasive LUAD. (**B**) Statistical analysis of METTL7B^+^EPCAM^+^cell proportion. (**C**) Statistical analysis of ITGA3^+^EPCAM^+^cell proportion. Scale bar: 50 μm. n = 3; *** *p* < 0.001.

To assess the clinical relevance of METTL7B and ITGA3, immunofluorescence staining was performed on glioblastoma tissues and matched paracancerous brain samples. METTL7B and ITGA3 were undetectable in paracancerous tissues but were robustly expressed in glioblastoma specimens. Importantly, both proteins exhibited significantly higher fluorescence intensity in high-grade glioblastoma (HGG) compared with low-grade glioblastoma (LGG) tumors, indicating a positive association between METTL7B–ITGA3 expression and glioblastoma malignancy (Figure 5A–C). Consistent findings were observed in lung adenocarcinoma samples. Immunofluorescence analysis revealed minimal or absent expression of METTL7B and ITGA3 in paracancerous lung tissues, whereas both proteins were clearly detected in minimally invasive (MIA) and invasive (IA) LUAD specimens. Notably, fluorescence intensity for both METTL7B and ITGA3 was markedly increased in invasive LUAD relative to minimally invasive lesions, demonstrating a stepwise upregulation associated with increasing tumor invasiveness (Figure 6A–C). These results above revealed that METTL7B and ITGA3 were upregulated expressed in glioblastoma and LUAD as the tumor progression.

### 3.7. METTL7B Enhances Tumor Cell Migration and Invasion

**Figure 7:**
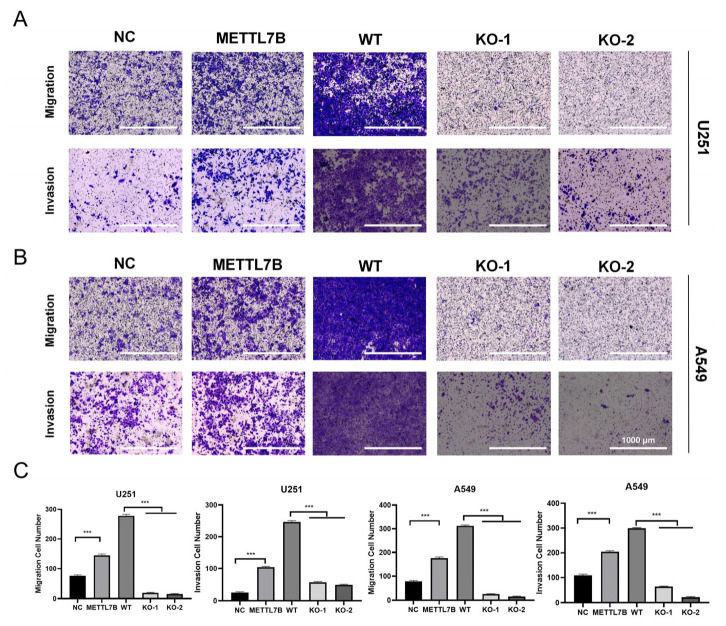
METTL7B promotes the migration and invasion of glioblastoma and lung adenocarcinoma cells. (**A**) Representative images of migration and invasion assays in U251 cells with METTL7B overexpression or knockout. (**B**) Representative images of migration and invasion assays in A549 cells with METTL7B overexpression or knockout. (**C**) Quantification of migrated and invaded cells in U251 and A549 cell groups. METTL7B overexpression significantly enhanced migration and invasion, whereas METTL7B knockout suppressed both processes. Scale bar: 1000 μm. *n* = 3; *** *p* < 0.001.

To determine functional impact of METTL7B on tumor aggressiveness, we performed transwell migration and invasion assays in glioblastoma and LUAD cell lines. In U251 glioblastoma cells, METTL7B overexpression markedly increased cell migration and invasion compared with control, while METTL7B knockout significantly suppressed both phenotypes relative to wild-type cells (Figure 7A,C). A similar pattern was observed in A549 lung adenocarcinoma cells, where METTL7B overexpression promoted migration and invasion, whereas METTL7B knockout inhibited these processes (Figure 7B,C). These findings demonstrate that METTL7B contributes to invasive and migratory potential. Moreover, METTL7B can suppress tumor progression and invasion. Addressing these questions will further refine the therapeutic potential of METTL7B-centered interventions for limiting invasive and aggressive tumor behavior.

## 4. Discussion

In this study, we combined single-cell transcriptomic analysis with functional validation to delineate the role of METTL7B in LUAD and glioblastoma. Our data demonstrated that METTL7B was significantly upregulated in both cancers, with preferential enrichment in epithelial cells in LUAD and astrocytic cells in glioblastoma. At the single-cell level, METTL7B-positive tumor cells showed extensive transcriptional reprogramming relative to METTL7B-negative counterparts, converging on cell adhesion and invasion pathways. Functional assays confirmed that METTL7B regulates ITGA3 expression and enhances tumor cell migration and invasion. Importantly, immunofluorescence staining revealed that both METTL7B and ITGA3 were absent in paracancerous tissues but progressively increased from mini-invasive to invasive LUAD, and from low-grade to high-grade glioblastoma, highlighting their association with tumor aggressiveness. The similar heterogeneity of tumors and the interactions between dynamic tumor microenvironments result in the poor prognosis and rapid progression in LUAD and glioblastoma patients. ScRNA-seq allowed us to identify lineage-specific enrichment of METTL7B within malignant epithelial populations in LUAD and astrocytic populations in glioblastoma-findings that would be obscured in bulk transcriptomic datasets. This restricted enrichment indicated that METTL7B was not a generalized stress-response gene, but instead exerted lineage-specific oncogenic functions. Functional assays further confirmed that METTL7B promoted invasion and migration, suggesting that METTL7B may serve as a putative prognostic factor in malignant progression.

Previous studies support our findings and underscore oncogenic action of METTL7B. In NSCLC, Liu et al. reported that METTL7B was essential for cancer progression, acting through cell cycle regulation and CCND1-mediated G1/S progression [17]. Consistently, Ali et al. and Li et al. both demonstrated that METTL7B was overexpressed in LUAD tissues and correlates with poor prognosis [26,27]. These clinical associations are aligned with the current observation that METTL7B is enriched in malignant epithelial cells and promotes invasive phenotypes. Moreover, Song et al. showed that METTL7B contributed to EGFR-TKI resistance in LUAD by regulating redox metabolism and m^6^A RNA modification, and that silencing METTL7B re-sensitizes resistant tumors to gefitinib and osimertinib [16]. Together, these studies establish METTL7B as a mediator of proliferation, invasion and therapeutic resistance, highlighting its multifaceted role in lung cancer biology. In glioblastoma, converging evidence also points to METTL7B as a determinant of malignancy. Xu et al. found that METTL7B promoted glioblastoma progression by suppressing EGR1 expression, leading to enhanced proliferation [28]. Constantinou et al. further demonstrated that METTL7B regulates lineage specification in glioblastoma by directing neural stem cell-to-astrocyte differentiation trajectories, thereby influencing both tumor size and invasiveness [29]. Pan-cancer and glioma-specific analyses additionally revealed that high METTL7B expression correlates with poor prognosis, immune infiltration, and immune checkpoint expression [30,31]. These findings complement the present data, which identified astrocytic enrichment of METTL7B and linked its expression with invasive transcriptional programs and glioblastoma grade.

Our results also identify *ITGA3* as a robust downstream effector of *METTL7B* in both LUAD and glioblastoma. Given the well-established role of integrin signaling in invasion, angiogenesis, and therapy resistance, the identification of *ITGA3* as a consistent *METTL7B*-regulated target suggests a unifying mechanism by which *METTL7B* promotes cell adhesion–tumor communication. The immunofluorescence findings, showing stronger METTL7B and ITGA3 expression in invasive LUAD compared with mini-invasive lesions, further underscore this mechanism and provide a histopathological correlate to the transcriptional and functional data. In LUAD, early disease appears to co-opt a transitional injury/plasticity state dependent on a KRAS-ITGA3-SRC axis [32], suggesting ITGA3 contributes to the initiation-phase circuitry. At the post-transcriptional level, S100A16 stabilizes *ITGA3* mRNA via MOV10 to maintain ECM-receptor signaling and malignant phenotypes in LUAD [33]. In glioblastoma, nanoparticle-delivered si-lncRNA NONHSAT159592.1 suppresses proliferation, Epithelial–Mesenchymal Transition (EMT), and invasion through ITGA3-related pathway, and ITGA3 overexpression reverses these inhibitory effects [34], directly supporting an ITGA3-anchored pro-invasive signaling route. Complementarily, ITGA3 drives glioblastoma stemness and invasion via POU3F2 regulation; ITGA3 knockdown or anti-ITGA3 antibody reduces tumor growth and stemness markers in vivo [35]. Nakada et al. demonstrated that ITGA3 was overexpressed in glioma stem-like cells, localized to invasive fronts and perivascular niches, and drived invasion through ERK1/2 signaling [36]. Findings position *METTL7B* as an upstream regulator of integrin-driven signaling programs that shape the invasive behavior in both lung adenocarcinoma and glioblastoma. The coordinated upregulation of *METTL7B* and *ITGA3* during the transition to invasive LUAD and high-grade glioblastoma underscores their potential value as biomarkers of tumor aggressiveness. Collectively, these observations support a model in which METTL7B promotes invasive progression by coupling malignant cell states to cell adhesion signaling, thereby identifying METTL7B and its downstream pathways as attractive targets for therapeutic strategies aimed at restraining tumor invasion.

While this study provides mechanistic insight into METTL7B-mediated tumor invasion, several aspects warrant further investigation. A limitation of this study is the use of only two independent Clustered Regularly Interspaced Short Palindromic Repeats-associated protein 9 (CRISPR-Cas9) knockout clones without systematic off-target profiling. While the two clones showed consistent phenotypes, we cannot completely exclude the possibility that clone-specific off-target effects may have contributed to the observed results. Future studies employing multiple independent sgRNAs will be necessary to definitively establish the specific role of METTL7B in the progression of LUAD. Secondly, expansion of single-cell and spatial analyses to larger, independent clinical cohorts will be valuable for validating the clinical relevance of METTL7B-defined invasive states. Moreover, delineating the precise molecular mechanisms through which METTL7B regulates integrin and cell adhesion-associated genes whether through transcriptional, post-transcriptional, or epigenetic control represents an important direction for future research. Finally, in vivo functional studies will be essential to determine the extent to which targeting METTL7B can suppress tumor progression and invasion. Addressing these questions will further refine the therapeutic potential of METTL7B-centered interventions for limiting invasive and aggressive tumor behavior.

## 5. Conclusions

This study identifies *METTL7B* as a potential lineage-enriched regulator of invasive progression in lung adenocarcinoma and glioblastoma. Through integrative single-cell transcriptomic analyses and functional validation, we demonstrate that METTL7B promotes tumor cell invasion by activating cell adhesion signaling, with ITGA3 emerging as a conserved downstream effector across both tumor types. The progressive upregulation of METTL7B in invasive and high-grade tumors underscores its clinical relevance as a biomarker of aggressiveness. Importantly, our findings position METTL7B as a putative prognostic factor, suggesting a potential link between METTL7B expression and cell adhesion signaling in tumor invasion in lung and brain cancers, pending further functional validation.

## Data Availability

The datasets generated for this study are available from the corresponding author GW upon reasonable request.

## Supplementary Materials

The following supporting information can be downloaded at https://www.mdpi.com/article/10.3390/cancers18091384/s1. File S1: Report of human A549 cell line authentication. File S2: Report of human U251 cell line authentication. Table S1: The DEGs list between tumor and normal groups in the METTL7B positive epithelial cells of LUAD; Table S2: The DEGs list between tumor and normal groups in the METTL7B positive astrocyte cells of Glioblastoma; Table S3: The DEGs list between the METTL7B positive group and METTL7B negative group in the malignant cell in LUAD; Table S4: The DEGs list between the METTL7B positive group and METTL7B negative group in the malignant cell in Glioblastoma.
