# Supplementary material for: Single-Cell Dissection Identifies METTL7B as Associated with Cell Adhesion-Mediated Tumor Invasion in Lung Adenocarcinoma and Glioblastoma
Source: Cancers (Basel). 2026 Apr 27;18(9):1384. doi: 10.3390/cancers18091384 (PMC13163069; doi:10.3390/cancers18091384)

Fig 4E NC,METTL7B-First replication

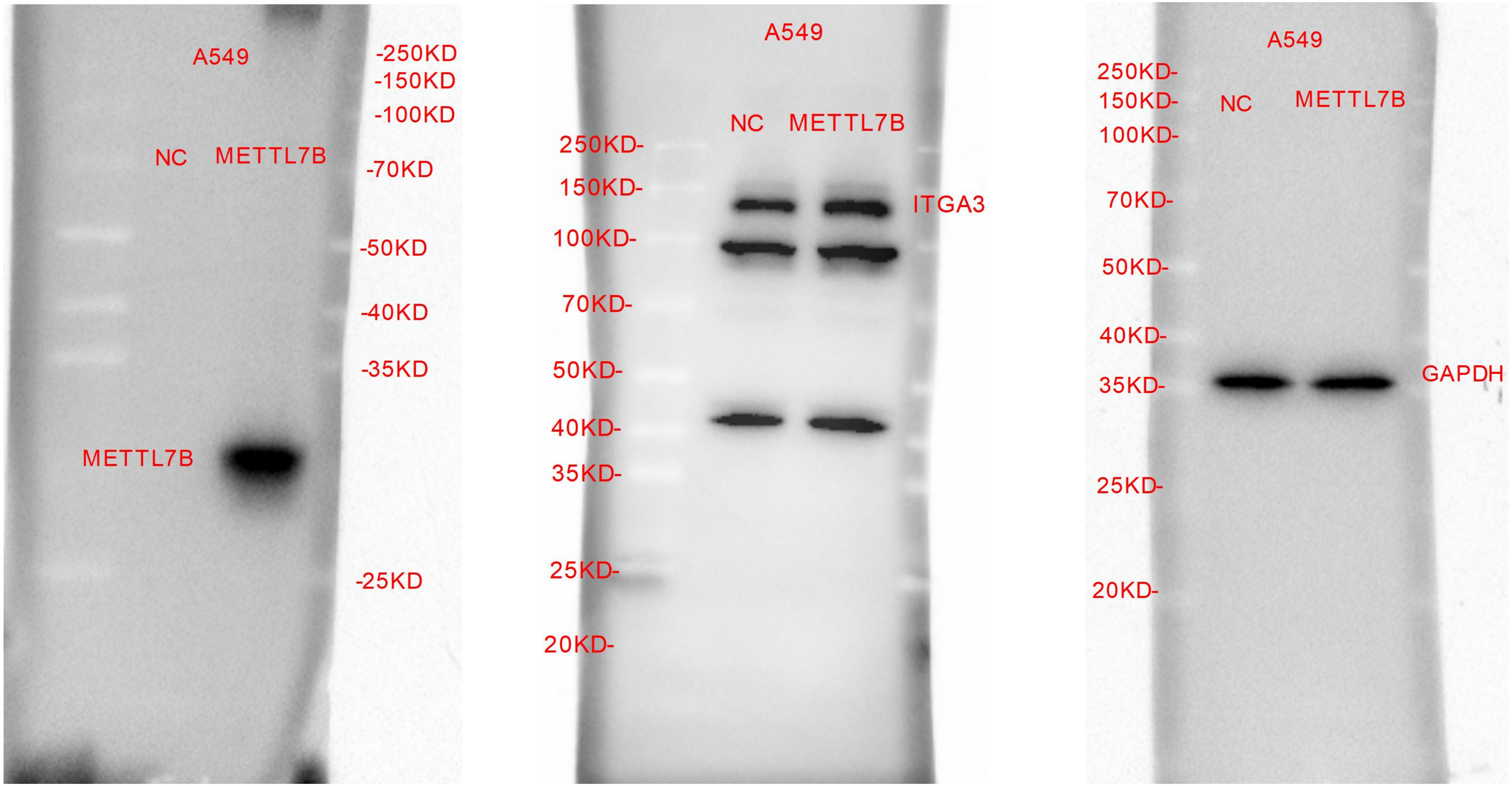

Fig 4E NC,METTL7B-Second replication

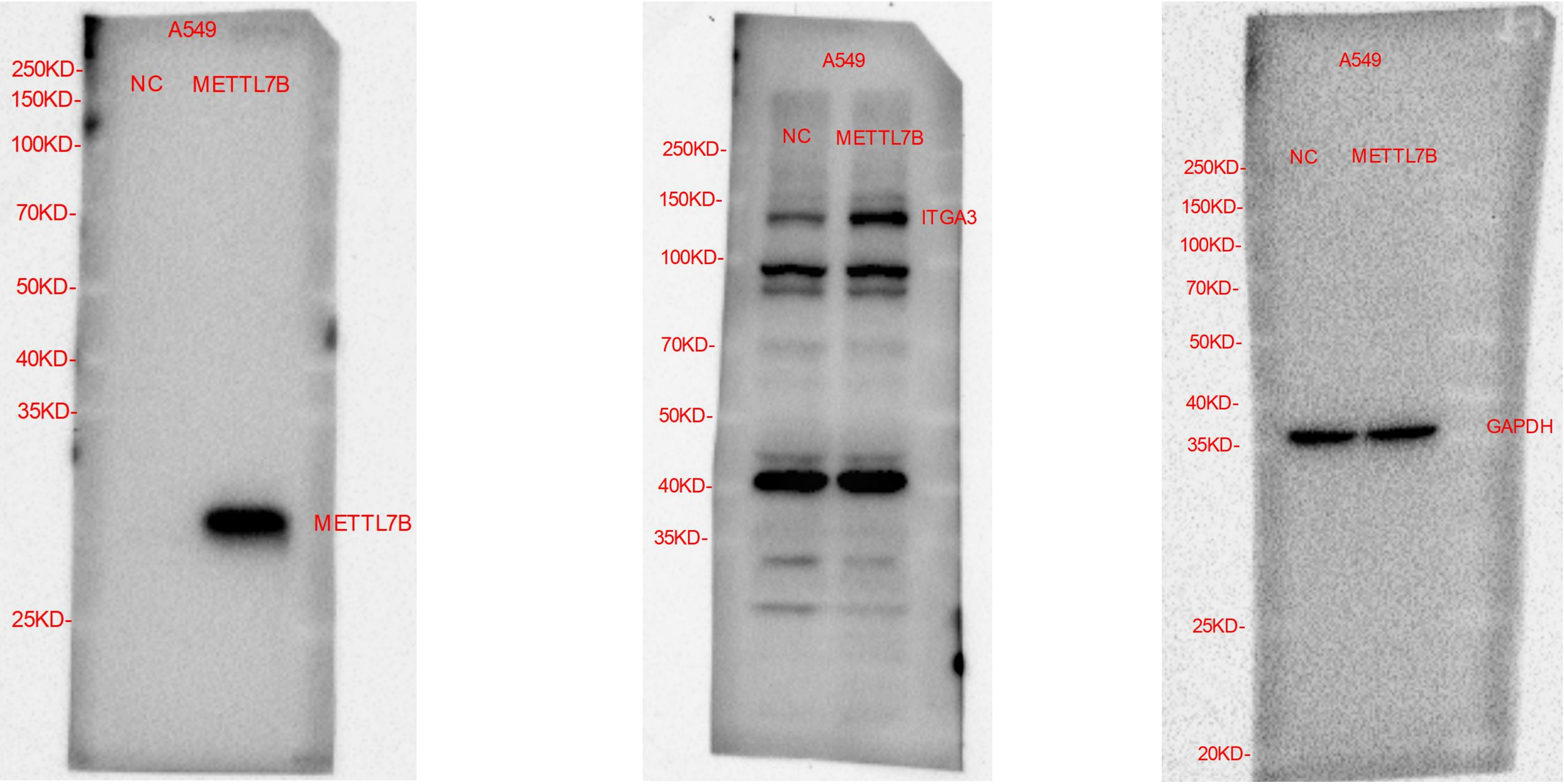

Fig 4E NC,METTL7B-Third replication

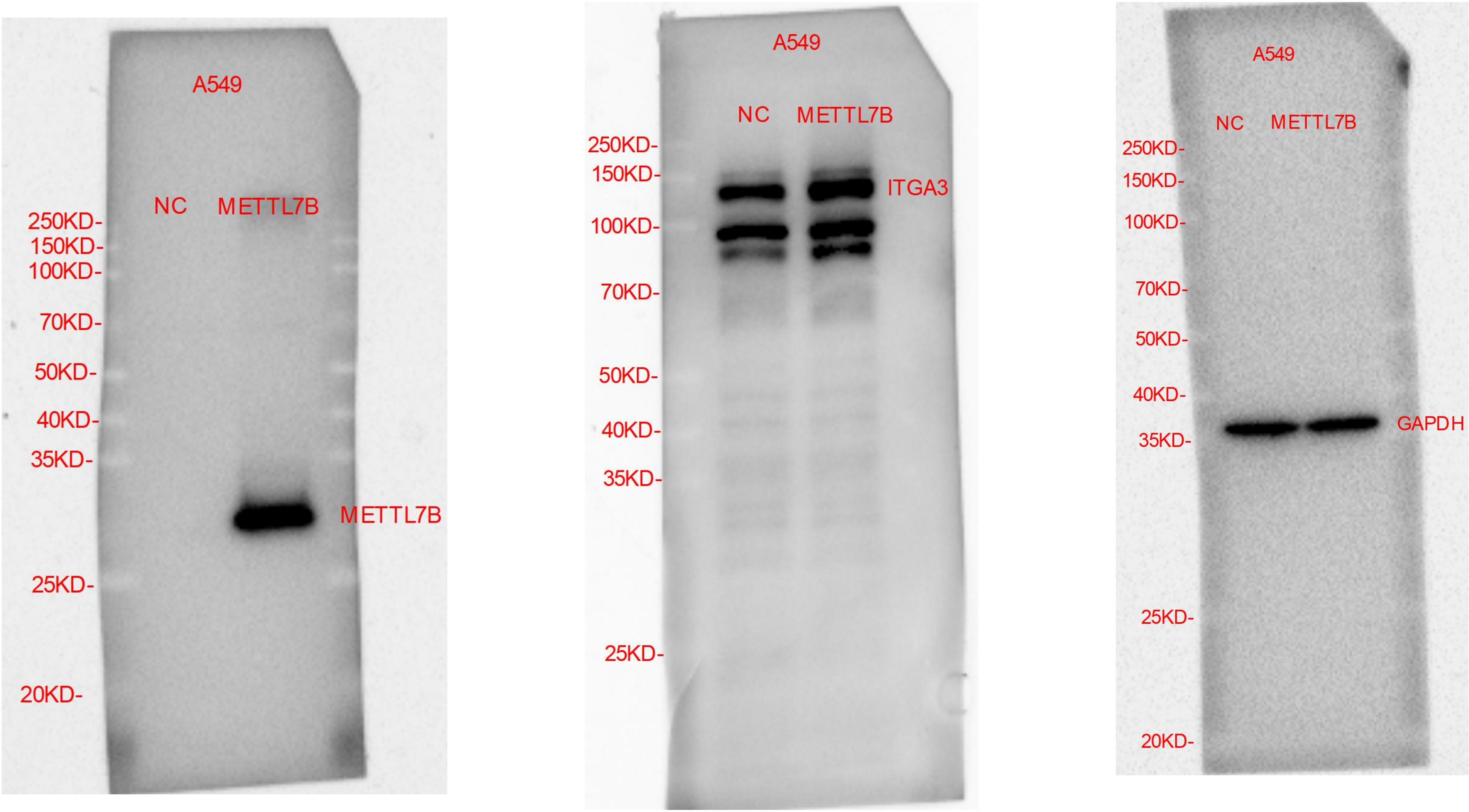

Fig 4E WT, KO1, KO2-First replication

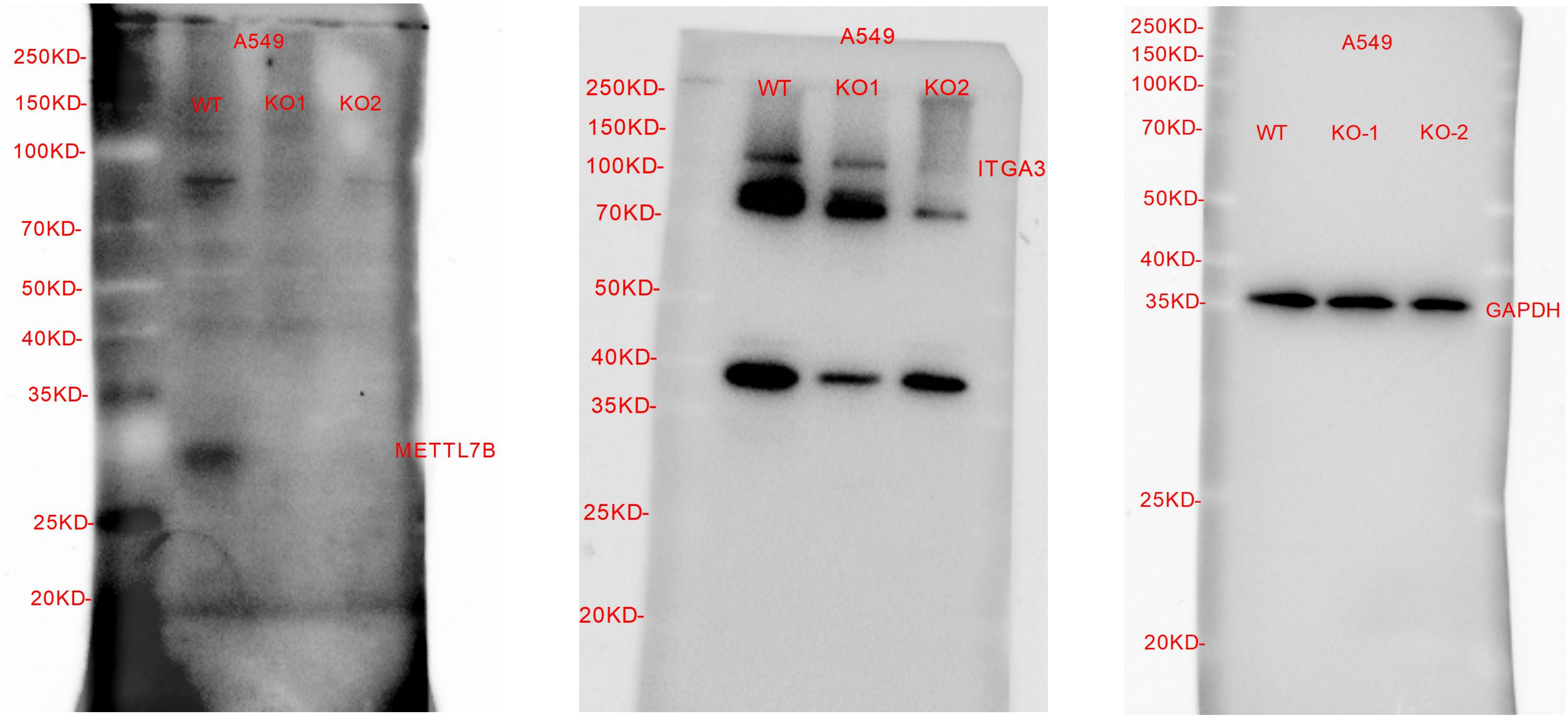

Fig 4E

WT, KO1, KO2-Second replication

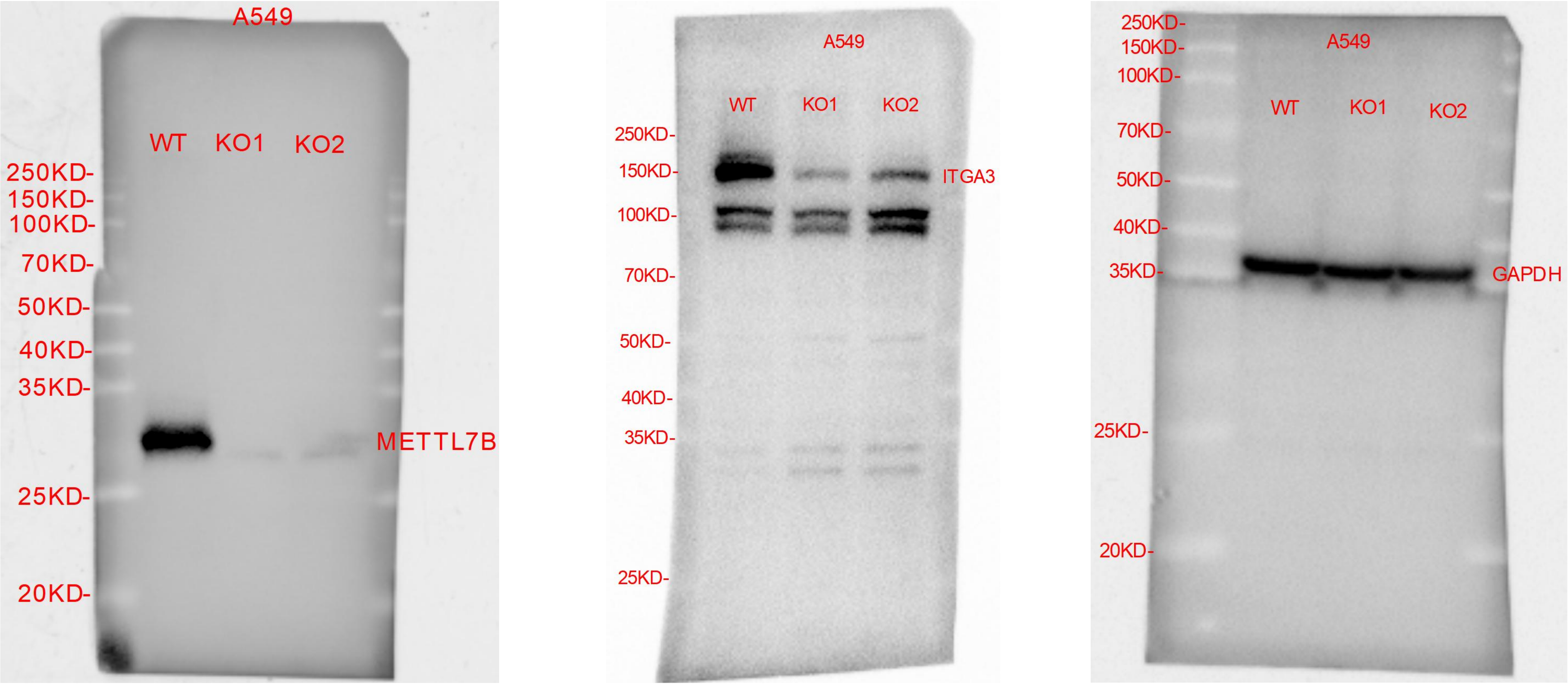

Fig 4E WT, KO1, KO2-Third replication

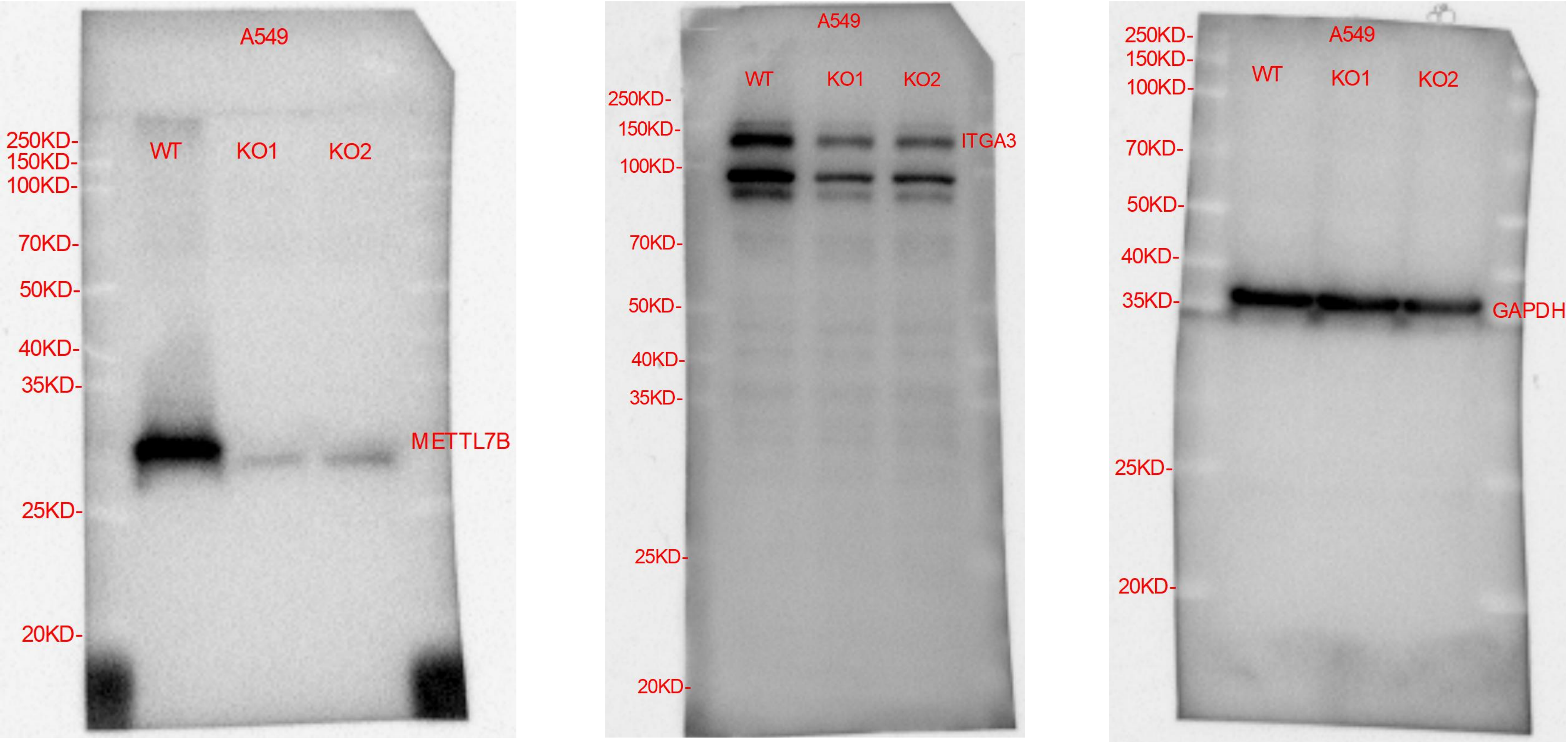

Fig 4F NC,METTL7B-First replication

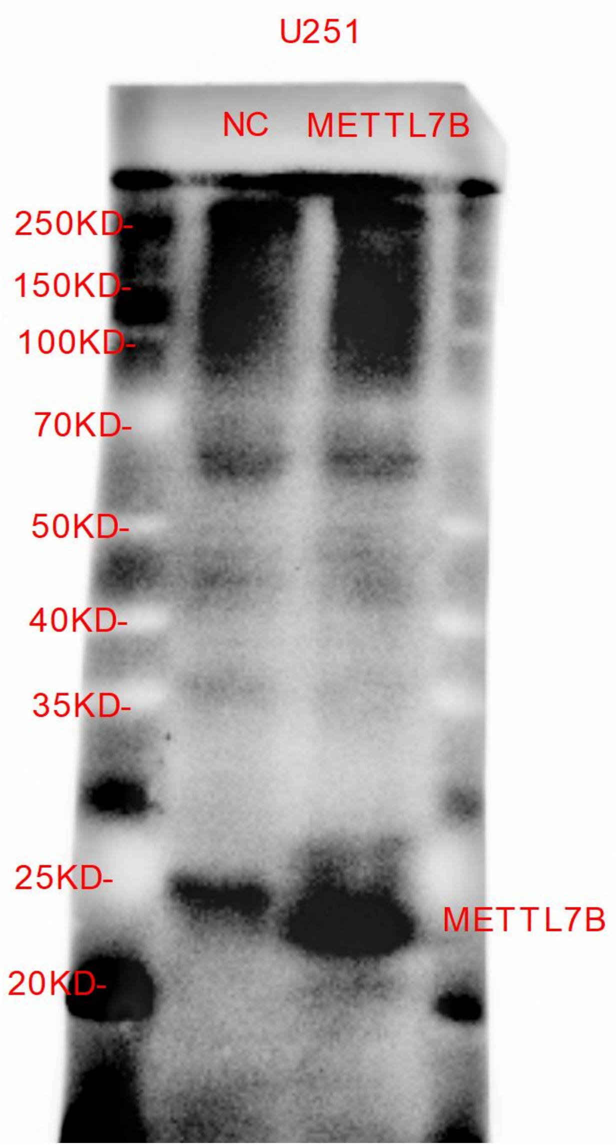

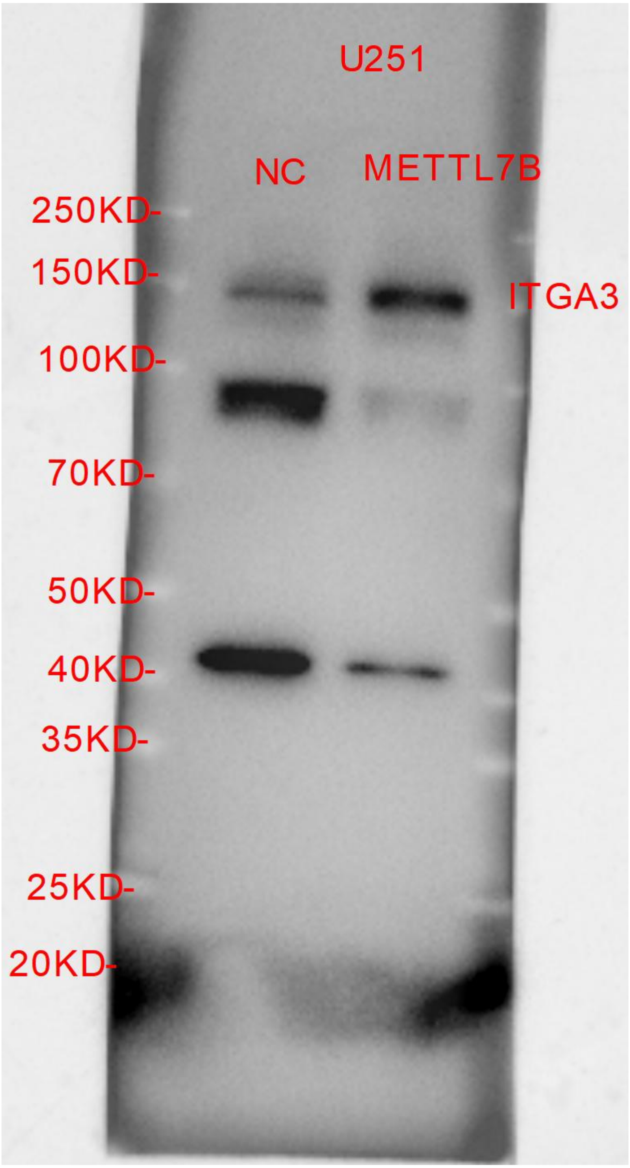

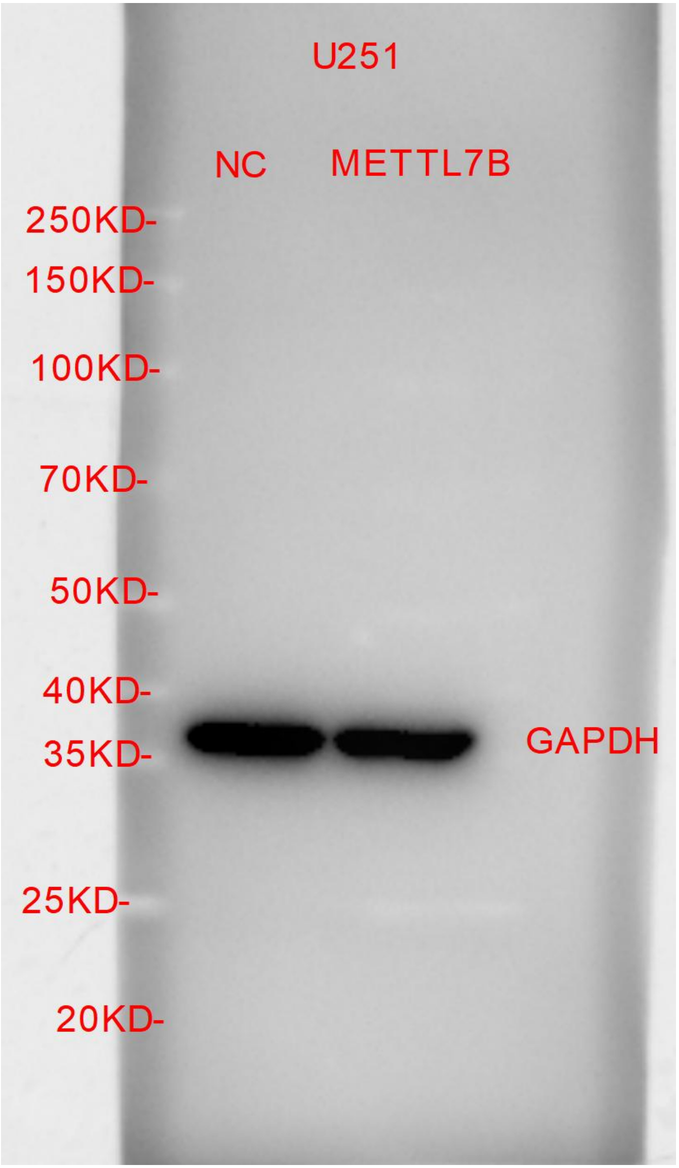

Fig 4F NC,METTL7B-Second replication

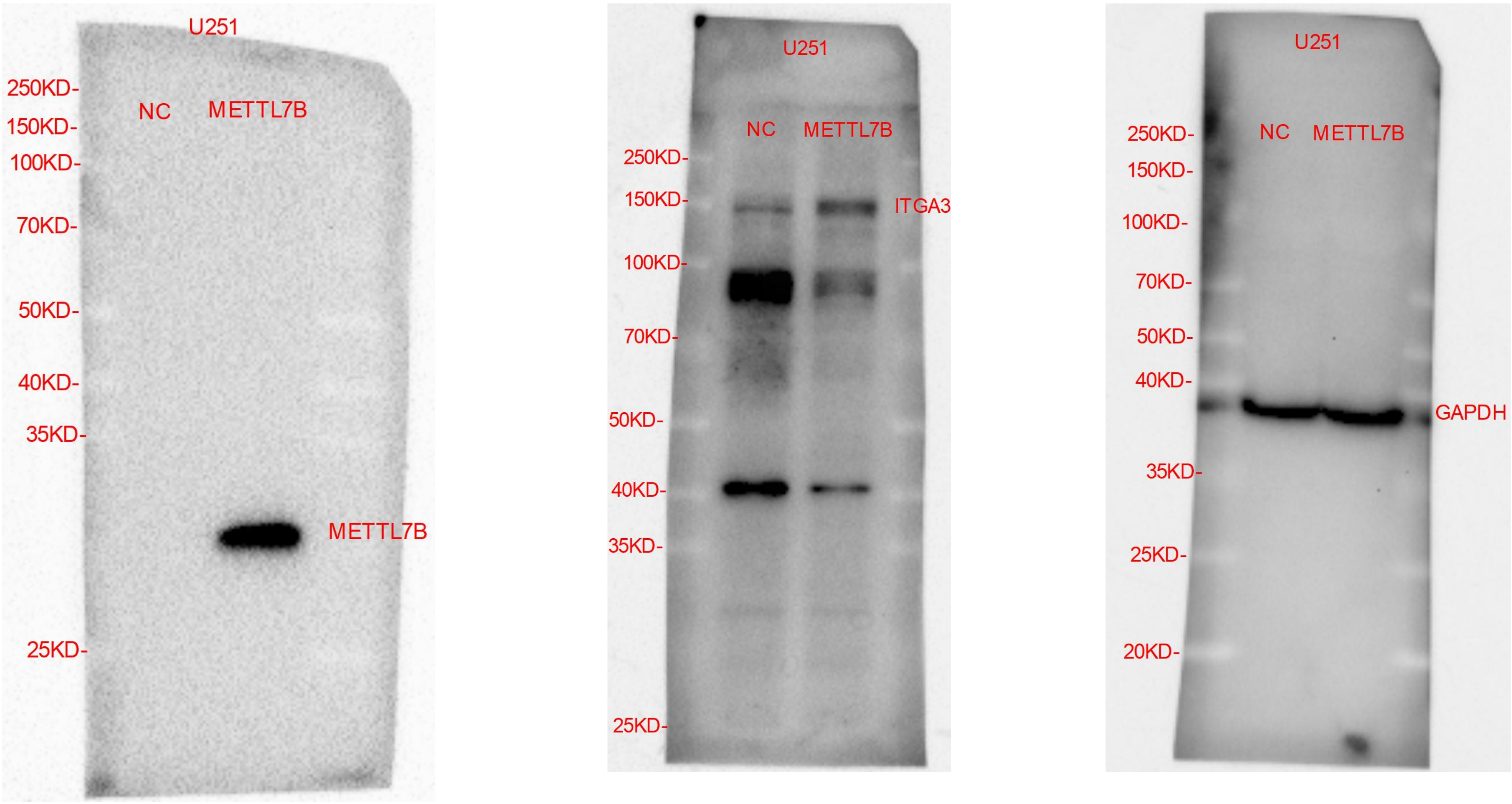

Fig 4F

NC,METTL7B-Third replication

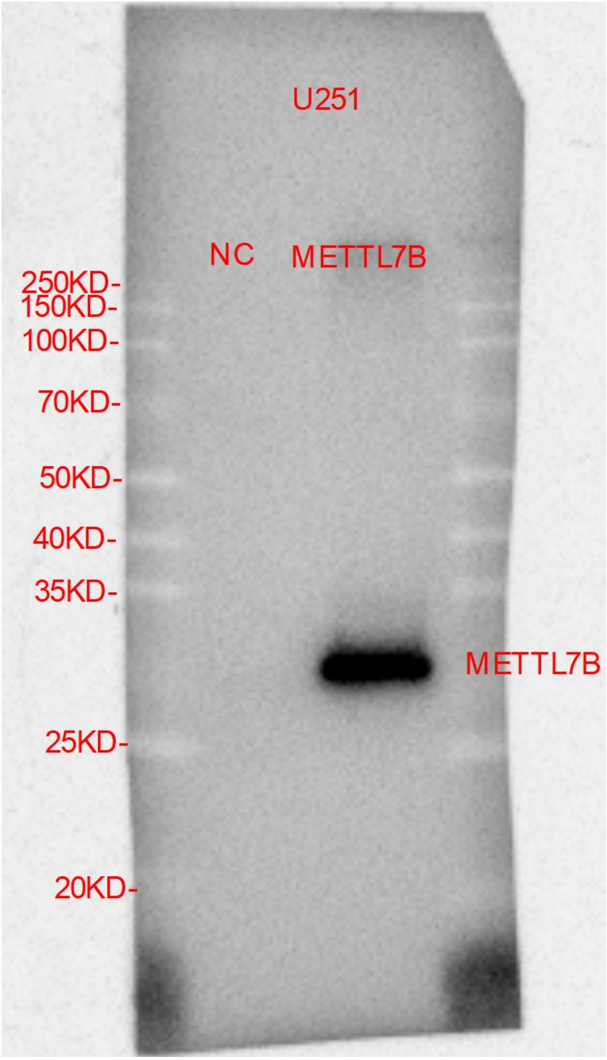

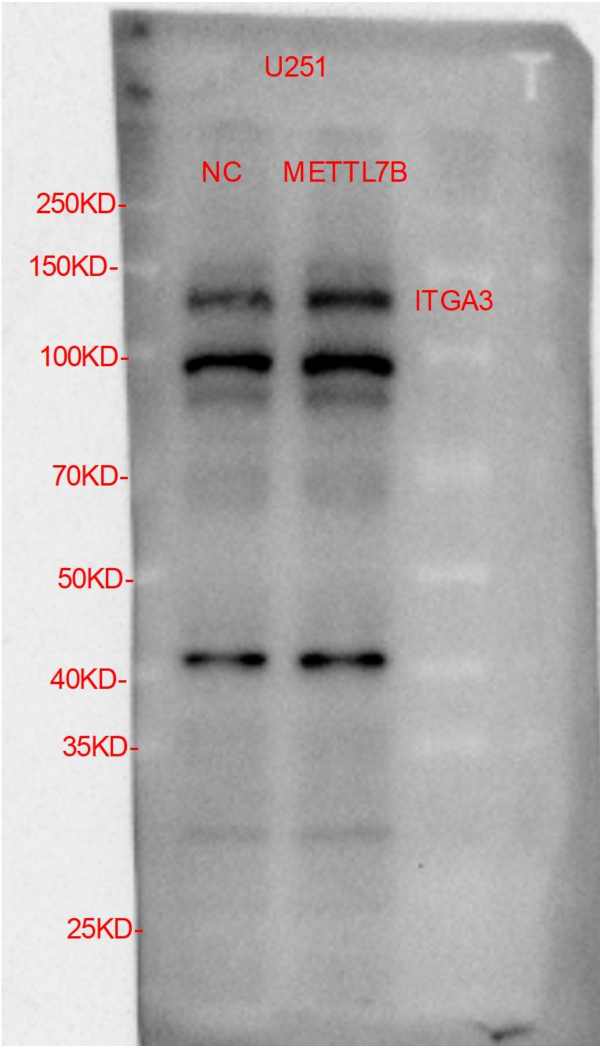

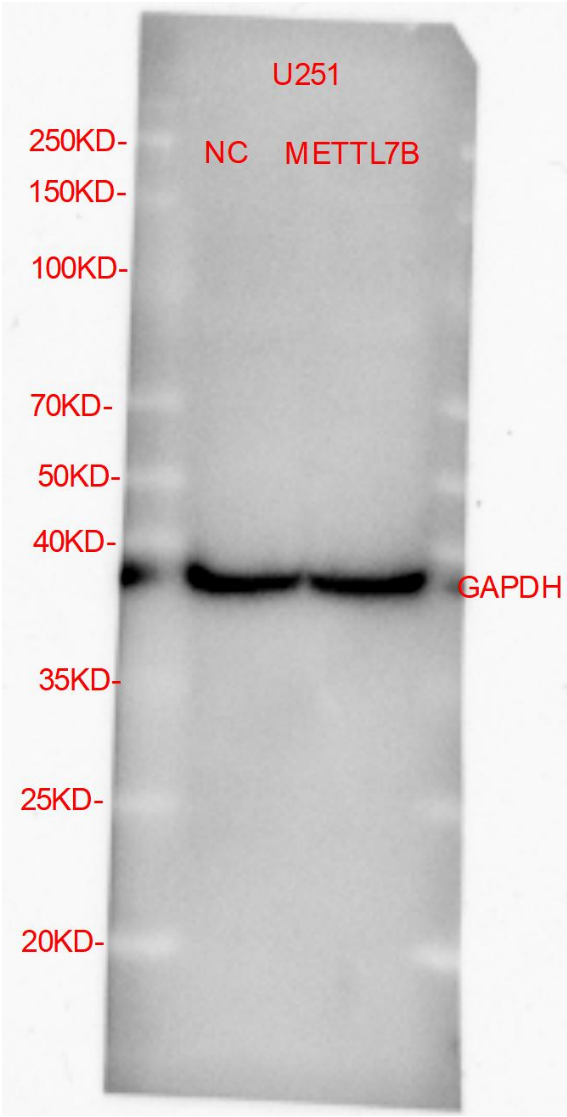

Fig 4F WT, KO1, KO2-First replication

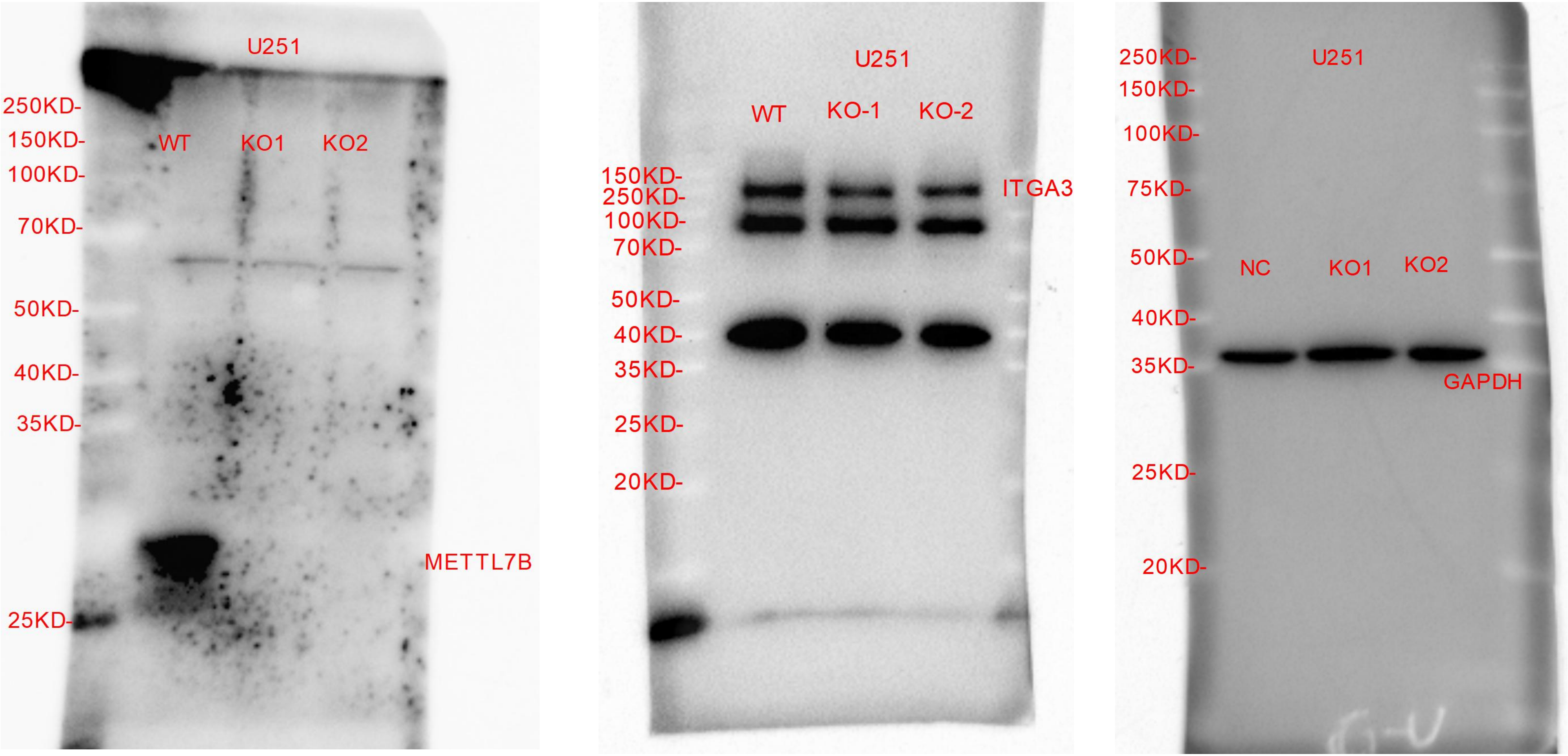

Fig 4F WT, KO1, KO2-Second replication

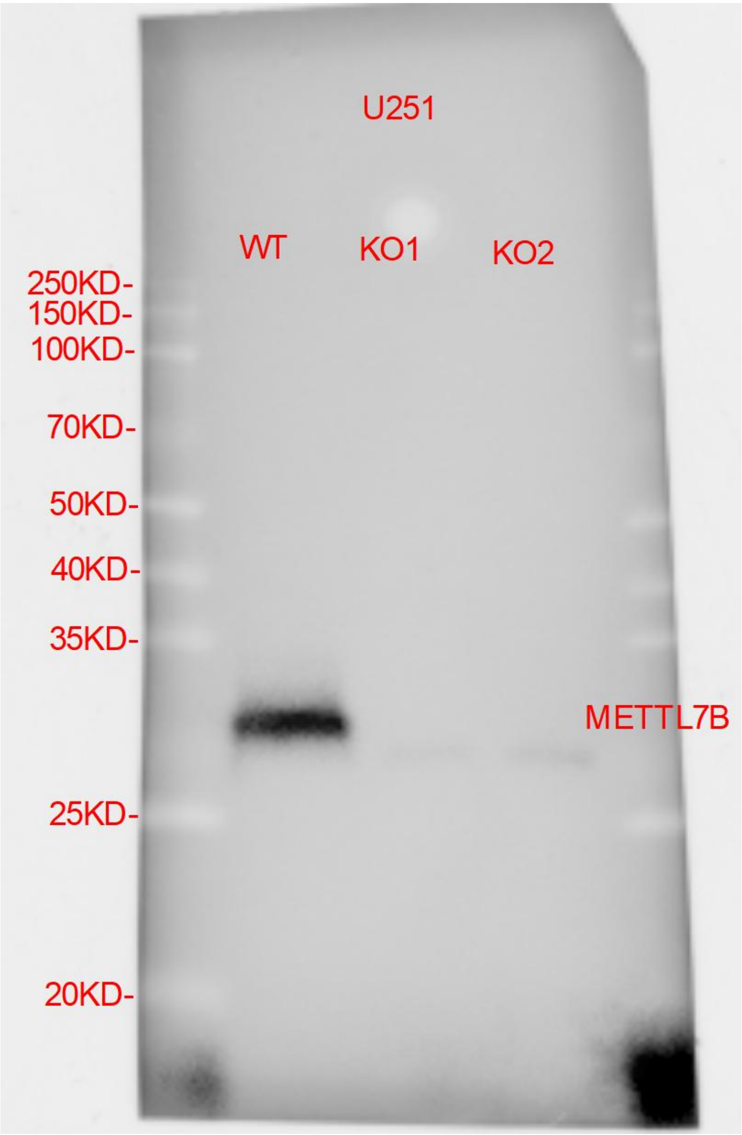

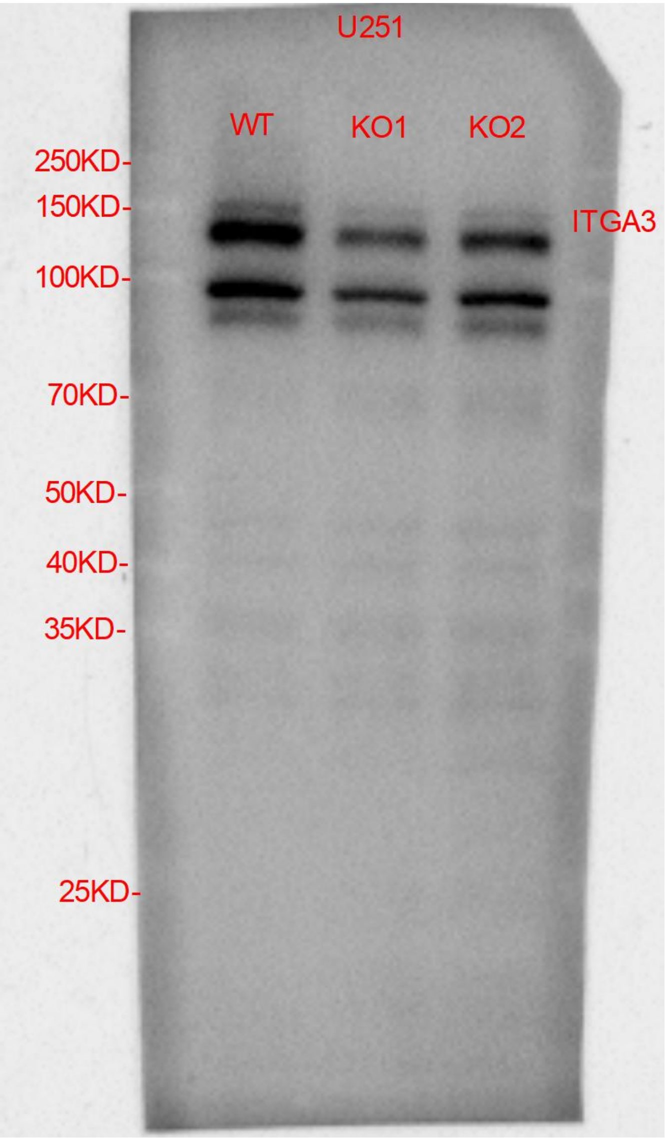

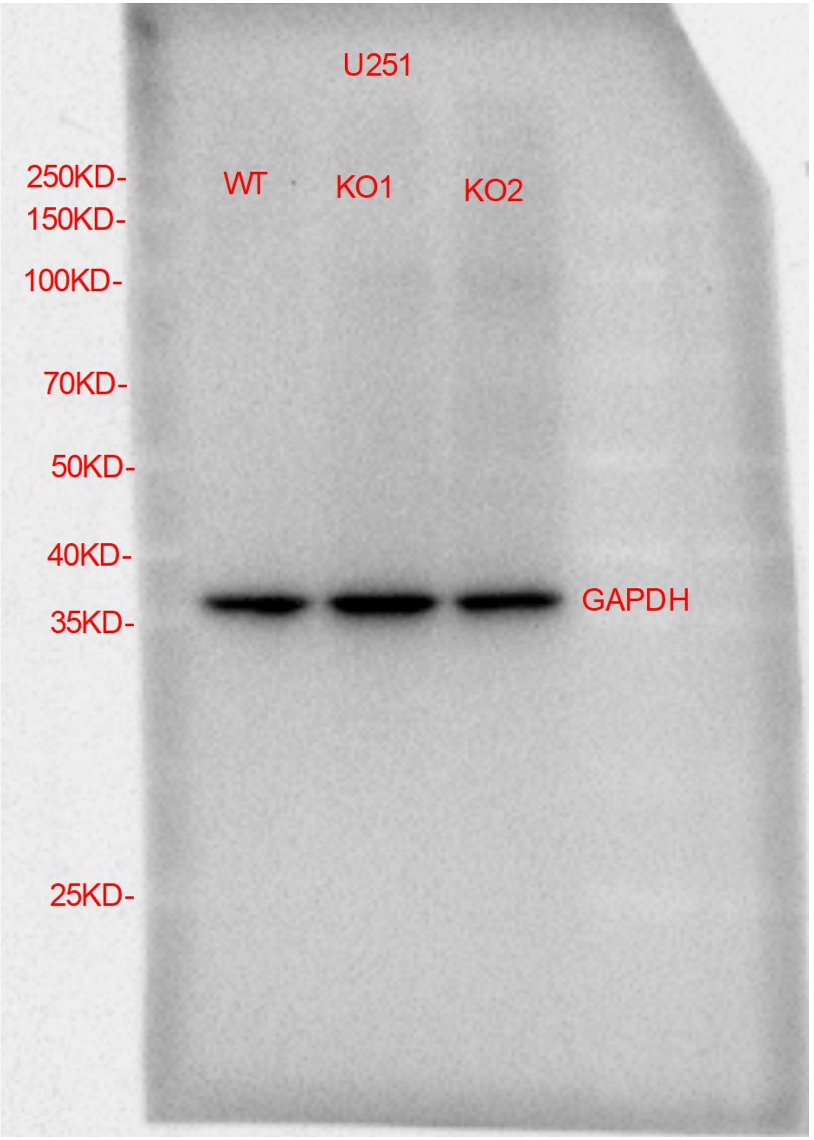

Fig 4F WT, KO1, KO2-Third replication

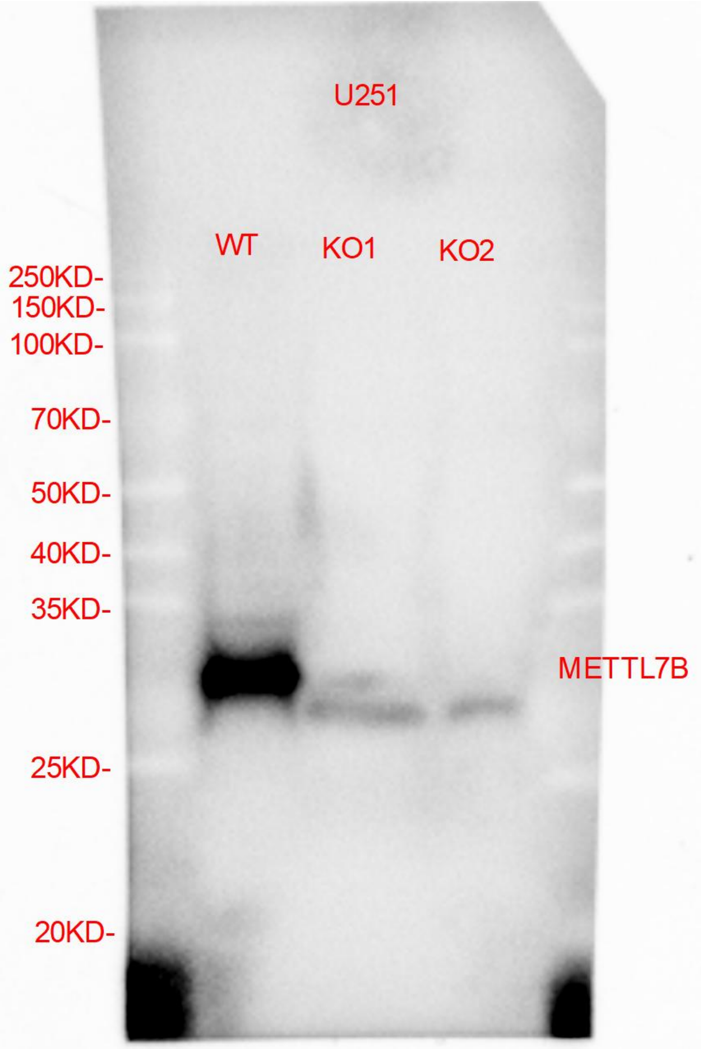

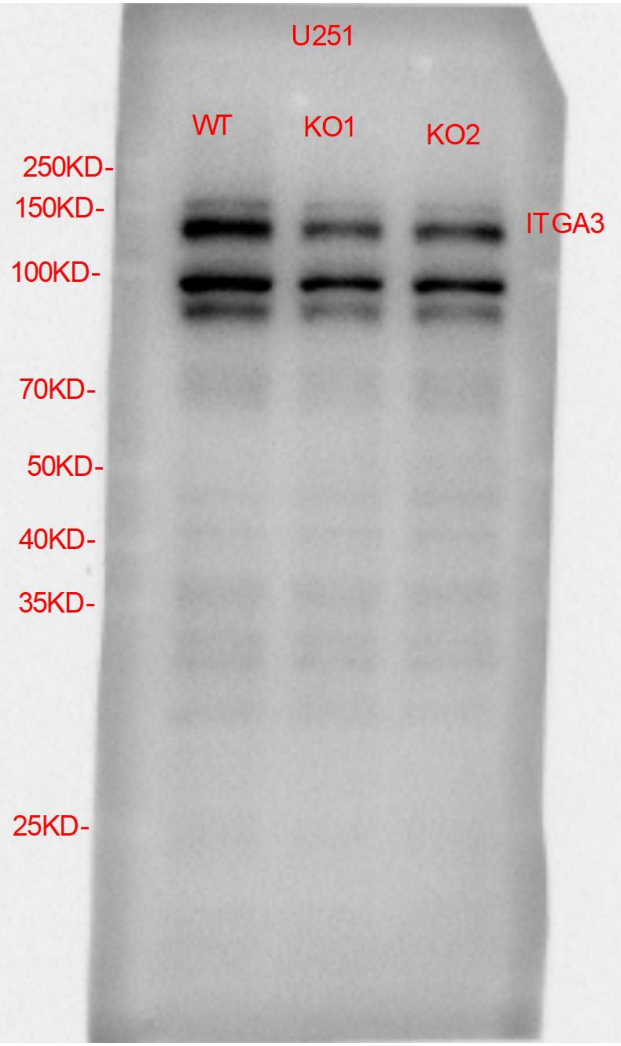

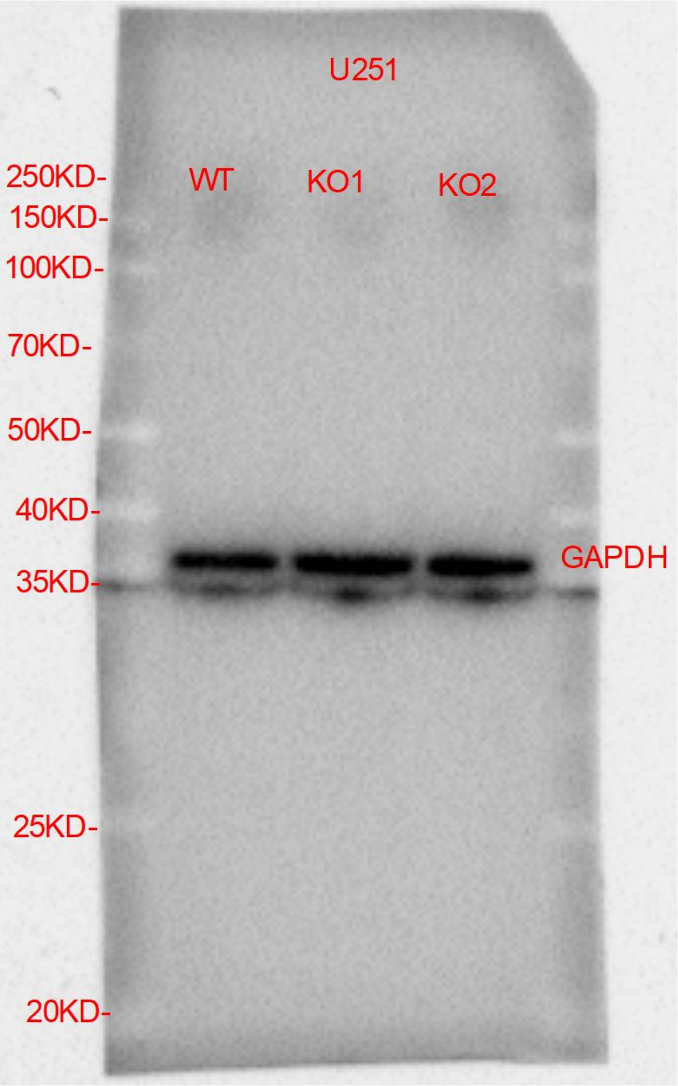

Supplement: Supplementary file 1 [file cancers-18-01384-s001.zip › original images/WB blots.pdf]
