# Supplementary material for: Single-Cell Dissection Identifies METTL7B as Associated with Cell Adhesion-Mediated Tumor Invasion in Lung Adenocarcinoma and Glioblastoma
Source: Cancers (Basel). 2026 Apr 27;18(9):1384. doi: 10.3390/cancers18091384 (PMC13163069; doi:10.3390/cancers18091384)
Supplement: Supplementary file 1 [file cancers-18-01384-s001.zip › supplementary File S1-A549 STR.pdf]

·

Report of Human Cell Line Authentication

Sample Name:	A549
Kinlogix Sales Order:	KJ0815
Receipt Date:	Apr.09th, 2026
Analysis Date:	Apr.13th, 2026

I. Method and Procedure

1. Twenty short tandem repeat (STR) loci plus the gender determining locus-Amelogenin, were amplified by PCR using a commercially available STR profiling Kit.
2. PCR products are assayed with 3730 DNA Analyzer (Applied Biosystems®).
3. Amplification of gene COI and electrophoresis are employed to survey the species of the sample.

II. Results

Number of have tri-alleles or tetra-alleles	No loci has tri-alleles or tetra-alleles.	Figure 1& Table 1
Compare STR data for the sample with the Expasy database	98.18% similarity to A549	Figure 2
Species of the sample	Human	Figure 3
Conclusion	To all above, the sample is a single cell line, and it is derived from a common ancestry with A549 cell line.	

Ps: Cell lines with ≥80% match are considered to be related; i.e., derived from a common ancestry.

Operator: Xiao Kang

Auditor: Qingtian Qin

GUANGZHOU KINLOGIX BIOTECH CO.,LTD. 2026.04.13 STR AUTHENTICATION

Guangzhou Kinlogix Biotech Co., Ltd.

Figure 1. STR profiles of the sample

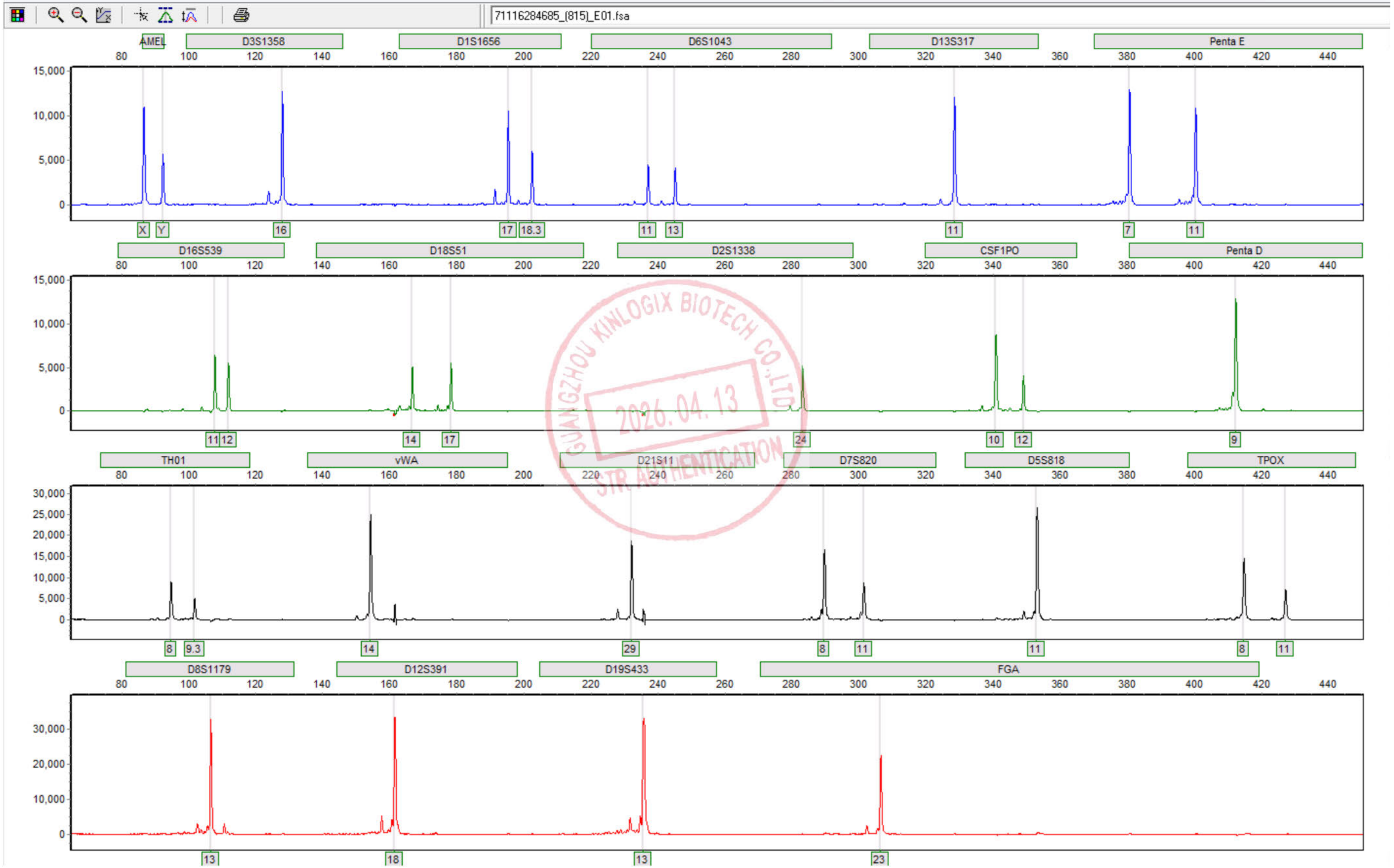

Table 1. STR profiles of the sample

21	Allele1	Allele2
AMEL	X	Y
D3S1358	16	
D1S1656	17	18.3
D6S1043	11	13
D13S317	11	
Penta E	7	11
D16S539	11	12
D18S51	14	17
D2S1338	24	
CSF1PO	10	12
Penta D	9	
TH01	8	9.3
vWA	14	
D21S11	29	
D7S820	8	11
D5S818	11	
TPOX	8	11
D8S1179	13	
D12S391	18	
D19S433	13	
FGA	23	

KINLOGIX BIOTECH CO.,LTD. 2026. 04. 13 STR AUTHENTICATION

Figure 2. Search result in ExPASy database

Accession	Name	Nº Markers	Score	Amel	CSF1PO	D1S1656	D2S1338	D3S1358	D5S818	D6S1043	D7S820	D8S1179	D12S391	D13S317	D16S539	D18S51	D19S433	D21S11	FGA	Penta D	Penta E	TH01	TPOX
NA	Query	NA	NA	X,Y	10,12	17,18.3	24	16	11	11,13	8,11	13	18	11	11,12	14,17	13	29	23	9	7,11	8,9.3	8,11
CVCL_A9MB	A549-Cas9-850	18	100.00%	X,Y	10,12		24	16	11	11,13	8,11	13	18	11	11,12	14,17	13	29	23		7,11	8,9.3	8,11
CVCL_A9ME	A549-Cas9-853	18	100.00%	X,Y	10,12		24	16	11	11,13	8,11	13	18	11	11,12	14,17	13	29	23		7,11	8,9.3	8,11
CVCL_E066	633	8	100.00%	X	10,12				11		8,11			11	11,12							8,9.3	8,11
CVCL_JK07	A549 EML4-ALK	8	100.00%	X	10,12				11		8,11			11	11,12							8,9.3	8,11
CVCL_VR66	A549 EML4-ALK-Luc2	8	100.00%	X	10,12				11		8,11			11	11,12							8,9.3	8,11
CVCL_LI35	A549 VIM RFP	8	100.00%	X,Y	10,12				11		8,11			11	11,12							8,9.3	8,11
CVCL_J242	A549-Luc [JCRB]	8	100.00%	X,Y	10,12				11		8,11			11	11,12							8,9.3	8,11
CVCL_UR31	A549-Luc2	8	100.00%	X,Y	10,12				11		8,11			11	11,12							8,9.3	8,11
CVCL_D425	ACE2plusC3	8	100.00%	X	10,12				11		8,11			11	11,12							8,9.3	8,11
CVCL_0023	A-549	19	98.18%	X,Y	10,12	17,18.3	24	16	11		8,11	13,14	18	11	11,12	14,17	13	29	23	9	7,11	8,9.3	8,11
CVCL_C0W4	A549/DDP	19	98.13%	X,Y	10,12		24	16	11	11,13	8,11	13,14	18	11	11,12	14,17	13	29	23	9	7,11	8,9.3	8,11
CVCL_XF57	A549-5Fu	18	98.11%	X,Y	10,12		24	16	11	11,13	8,11	13,14	18	11	11,12	14,17	13	29	23		7,11	8,9.3	8,11
CVCL_A9MC	A549 Cas9 851	18	98.11%	X,Y	10,12		24	16	11	11,13	8,11	13,14	18	11	11,12	14,17	13	29	23		7,11	8,9.3	8,11
CVCL_A9MD	A549-Cas9-852	18	98.11%	X,Y	10,12		24	16	11	11,13	8,11	13,14	18	11	11,12	14,17	13	29	23		7,11	8,9.3	8,11
CVCL_XE69	A549-Luc2-tdT-2	18	98.11%	X,Y	10,12		24	16	11	11,13	8,11	13,14	18	11	11,12	14,17	13	29	23		7,11	8,9.3	8,11
CVCL_XB30	A549-mCherry	18	98.11%	X,Y	10,12		24	16	11	11,13	8,11	13,14	18	11	11,12	14,17	13	29	23		7,11	8,9.3	8,11
CVCL_XB27	A549-tdT	18	98.11%	X,Y	10,12		24	16	11	11,13	8,11	13,14	18	11	11,12	14,17	13	29	23		7,11	8,9.3	8,11
CVCL_C3CC	A549 B2M KO	15	97.78%	X,Y	10,12			16	11		8,11	13,14		11	11,12	14,17		29	23	9	7,11	8,9.3	8,11
CVCL_C3DB	A549 EGFR Knockout	15	97.78%	X,Y	10,12			16	11		8,11	13,14		11	11,12	14,17		29	23	9	7,11	8,9.3	8,11
CVCL_E9XL	A549/cis	12	97.14%				24	16	11	11,13	8,11	13,14						29	23		7,11	8,9.3	8,11
CVCL_XE68	A549-Cas9-538	18	96.30%	X,Y	10,12		24	10	11	11,13	8,11	13,14	18,19	11	11,12	14,17	13	29	23		7,11	8,9.3	8,11
CVCL_VR73	A549 dCas9-KRAB	8	96.30%	X,Y	10,12				11		8,11			11	11,12,13							8,9.3	8,11
CVCL_C577	RE-25-HOM	9	96.00%	X,Y	10			10	11		8,11			11					23			8,9.3	8,11
CVCL_C585	SH-39-HOM	9	96.00%	X,Y	10			16	11		8,11			11					23			8,9.3	8,11

Figure 3. Authentication of the species of the sample

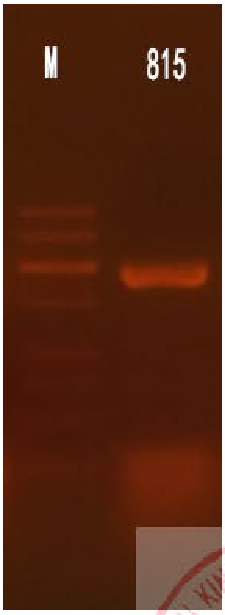

M: Marker. As the size of 600, 500, 400, 300, 200, 150, 100 and 50bp from up to down.

Eight species are checked, as follow: *Homo sapiens* 391bp, *Cricetulus griseus* 315bp, *Macaca mulatta* 287bp, *Cercopithecus aethiops* 222bp, *Rattus norvegicus* 196bp, *Canis familiaris* 172bp, *Mus musculus* 150bp, *Bos Taurus* 102bp.

KJ0815: The sample. The band size is 391bp which matches the size of human.
