# Supplementary material for: Single-Cell Dissection Identifies METTL7B as Associated with Cell Adhesion-Mediated Tumor Invasion in Lung Adenocarcinoma and Glioblastoma
Source: Cancers (Basel). 2026 Apr 27;18(9):1384. doi: 10.3390/cancers18091384 (PMC13163069; doi:10.3390/cancers18091384)
Supplement: Supplementary file 1 [file cancers-18-01384-s001.zip › supplementary File S2-U251 STR.pdf]

Report of Human Cell Line Authentication

Sample Name:	U251
Kinlogix Sales Order:	KJ0814
Receipt Date:	Apr.09th, 2026
Analysis Date:	Apr.13th, 2026

I. Method and Procedure

1. Twenty short tandem repeat (STR) loci plus the gender determining locus-Amelogenin, were amplified by PCR using a commercially available STR profiling Kit.
2. PCR products are assayed with 3730 DNA Analyzer (Applied Biosystems®).
3. Amplification of gene COI and electrophoresis are employed to survey the species of the sample.

II. Results

Number of have tri-alleles or tetra-alleles	No loci has tri-alleles or tetra-alleles.	Figure 1& Table 1
Compare STR data for the sample with the Expasy database	98.41% similarity to U-251MG	Figure 2
Species of the sample	Human	Figure 3
Conclusion	To all above, the sample is a single cell line, and it is derived from a common ancestry with U-251MG cell line.	

Ps: Cell lines with ≥80% match are considered to be related; i.e., derived from a common ancestry.

Operator: Xiao Kang

Auditor: Qingtian Qin

Guangzhou Kinlogix Biotech Co., Ltd.

Figure 1. STR profiles of the sample

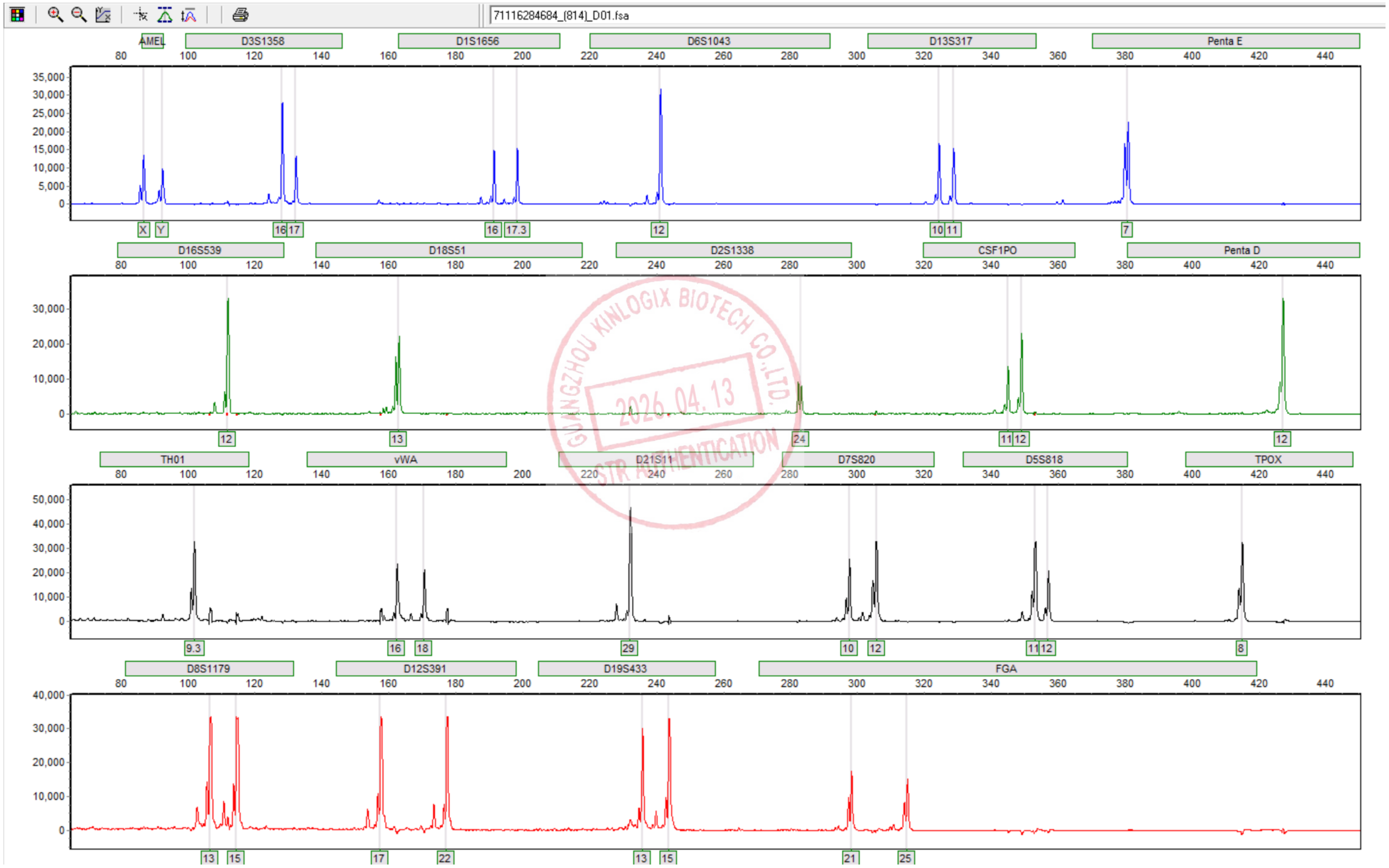

Table 1. STR profiles of the sample

21	Allele1	Allele2
AMEL	X	Y
D3S1358	16	17
D1S1656	16	17.3
D6S1043	12	
D13S317	10	11
Penta E	7	
D16S539	12	
D18S51	13	
D2S1338	24	
CSF1PO	11	12
Penta D	12	
TH01	9.3	
vWA	16	18
D21S11	29	
D7S820	10	12
D5S818	11	12
TPOX	8	
D8S1179	13	15
D12S391	17	22
D19S433	13	15
FGA	21	25

GUANGZHOU KINLOGIX BIOTECH CO.,LTD.
2026.04.13
STR AUTHENTICATION

Figure 2. Search result in ExPASy database

Accession	Name	№ Markers	Score	Amel	CSF1PO	D1S1656	D2S1338	D3S1358	D5S818	D6S1043	D7S820	D8S1179	D12S391	D13S317	D16S539	D18S51	D19S433	D21S11	FGA	Penta D	Penta E	TH01	TPOX
NA	Query	NA	NA	X,Y	11,12	16,17.3	24	16,17	11,12	12	10,12	13,15	17,22	10,11	12	13	13,15	29	21,25	12	7	9.3	8
CVCL_2864	B2-17	8	100.00%	X	11,12				11,12		10,12			10,11	12							9.3	8
CVCL_2800	KNS-89	8	100.00%	X	11,12				11,12		10,12			10,11	12							9.3	8
CVCL_B325	TK-1 [Human astrocyt	8	100.00%	X,Y	11,12				11,12		10,12			10,11	12							9.3	8
CVCL_J269	U-251MG-Luc	8	100.00%	X,Y	11,12				11,12		10,12			10,11	12							9.3	8
CVCL_0021 *Best*	U-251MG	20	98.41%	X	11,12	16,17.3	22,24	16,17	11,12	12	10,12	13,15	17,22	10,11	12	13	13,15	29	21,25	12	7	9.3	8
CVCL_0021 *Worst*	U-251MG	20	87.88%	X,Y	12,13	16,17.3	22,24	16,17	11	12	10,12	13,15	17,22	10,11	12	13	13,15	29,30	21,25	10,12	7,10	9.3	8,9
CVCL_0535 *Best*	SNB-19	17	96.30%	X	11,12		22,24	16,17	11,12		10,12	13,15		10,11	12	13	13,15	29	21,25	12	7,10	9.3	8
CVCL_0535 *Worst*	SNB-19	17	90.20%	X,Y	12		22,24	16,17	11,12		10	13,15		10,11	12	13	13,15	29	21	12	7,10	9.3	8
CVCL_2809	U-251MG (KO)	8	96.00%	X	11,12				11		10,12			10,11	12							9.3	8
CVCL_2219 *Best*	U-373MG ATCC	15	93.88%	X,Y	11,12			16,17	11,12		10,12	13,15		10,11	12	13		29,30	21,25	10,12	7,10	9.3	8
CVCL_2219 *Worst*	U-373MG ATCC	15	92.00%	X,Y	11,12			16,17	11,12		10,12	13,15		10,11	12	13		29,30	21,25	10,12	7,10	9.3,11	8

Figure 3. Authentication of the species of the sample

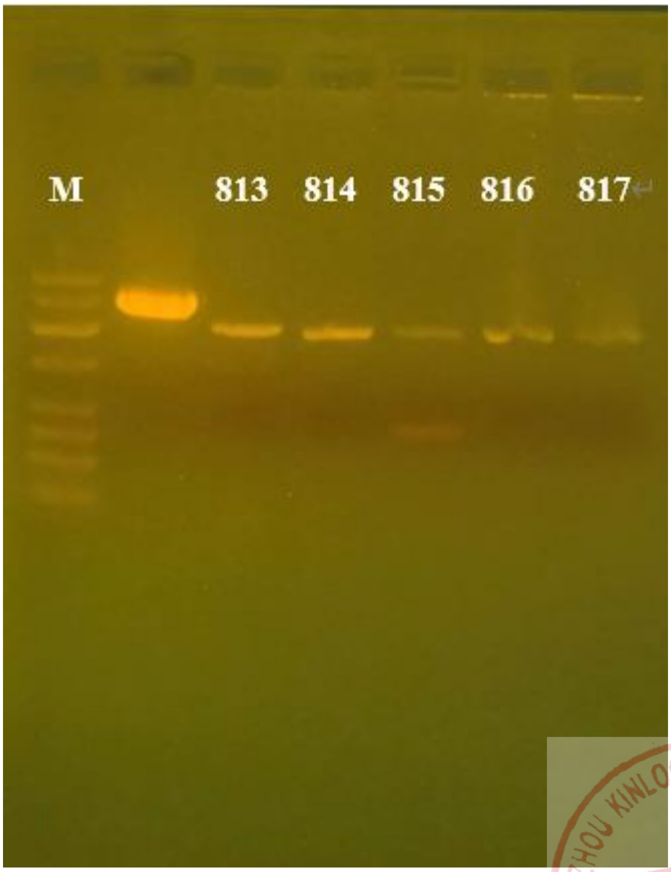

M: Marker. As the size of 600, 500, 400, 300, 200, 150, 100 and 50bp from up to down.

Eight species are checked, as follow: *Homo sapiens* 391bp, *Cricetulus griseus* 315bp, *Macaca mulatta* 287bp, *Cercopithecus aethiops* 222bp, *Rattus norvegicus* 196bp, *Canis familiaris* 172bp, *Mus musculus* 150bp, *Bos Taurus* 102bp.

KJ0814: The sample. The band size is 391bp which matches the size of human.

GUANGZHOU KINLOGIX BIOTECH CO.,LTD
2026.04.13
STR AUTHENTICATION
