# Supplementary figures and images for: Single-Cell Dissection Identifies METTL7B as Associated with Cell Adhesion-Mediated Tumor Invasion in Lung Adenocarcinoma and Glioblastoma
Source: Cancers (Basel). 2026 Apr 27;18(9):1384. doi: 10.3390/cancers18091384 (PMC13163069; doi:10.3390/cancers18091384)

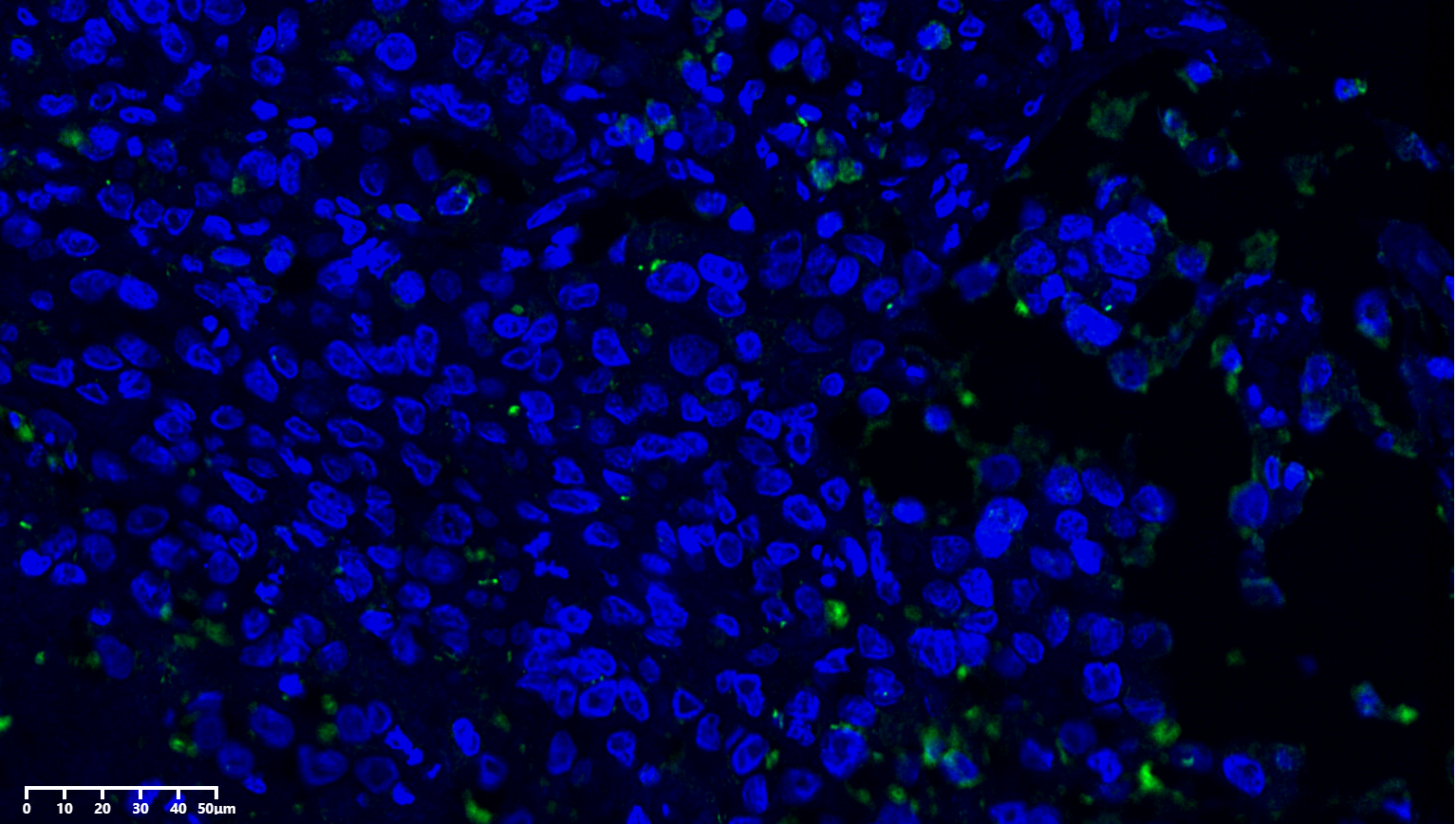

Supplement: Supplementary file 1 [file cancers-18-01384-s001.zip › original images/Microscopy/glioblastoma-1/HGG/7B.tif]

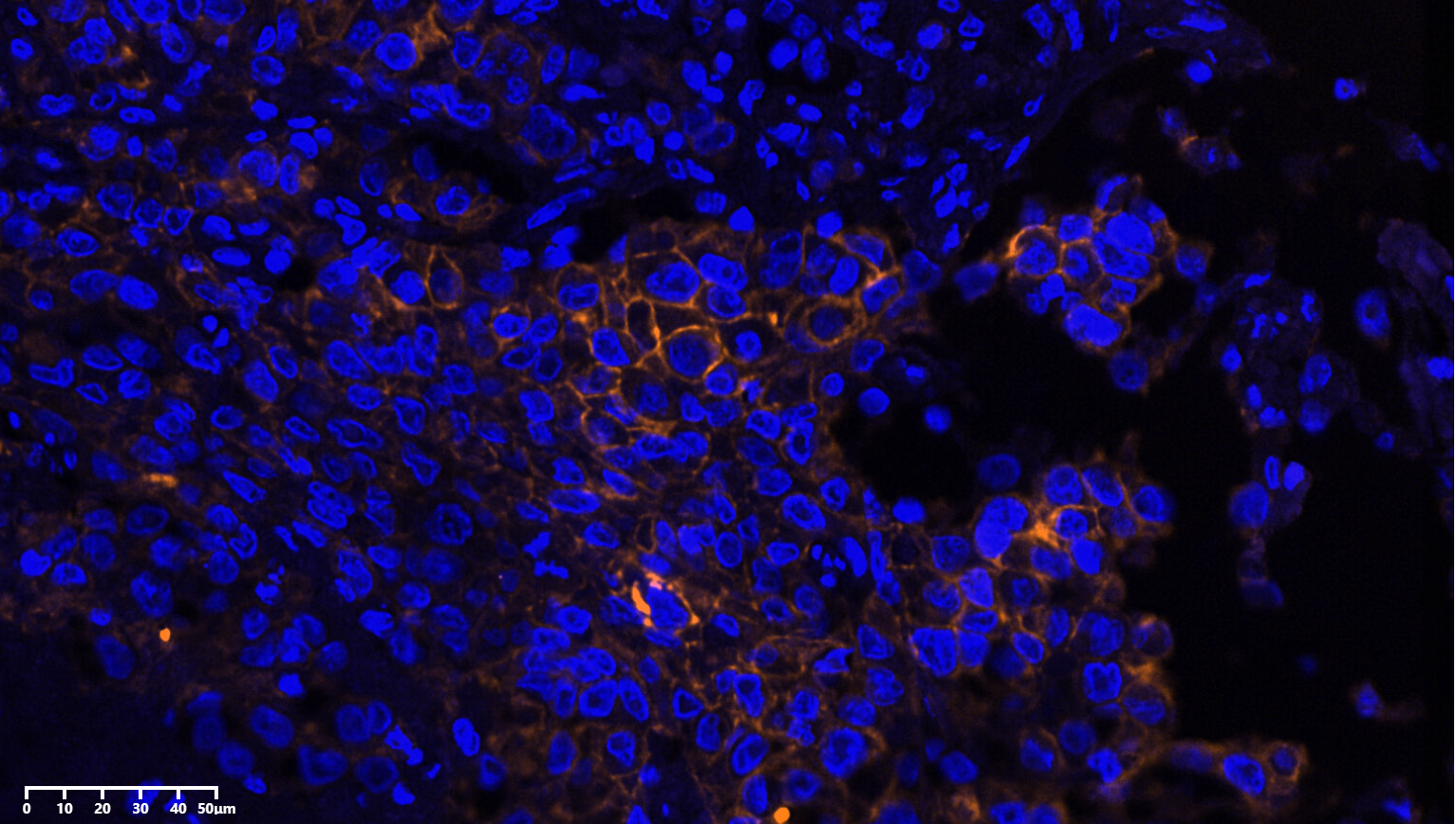

Supplement: Supplementary file 1 [file cancers-18-01384-s001.zip › original images/Microscopy/glioblastoma-1/HGG/EPCAM.tif]

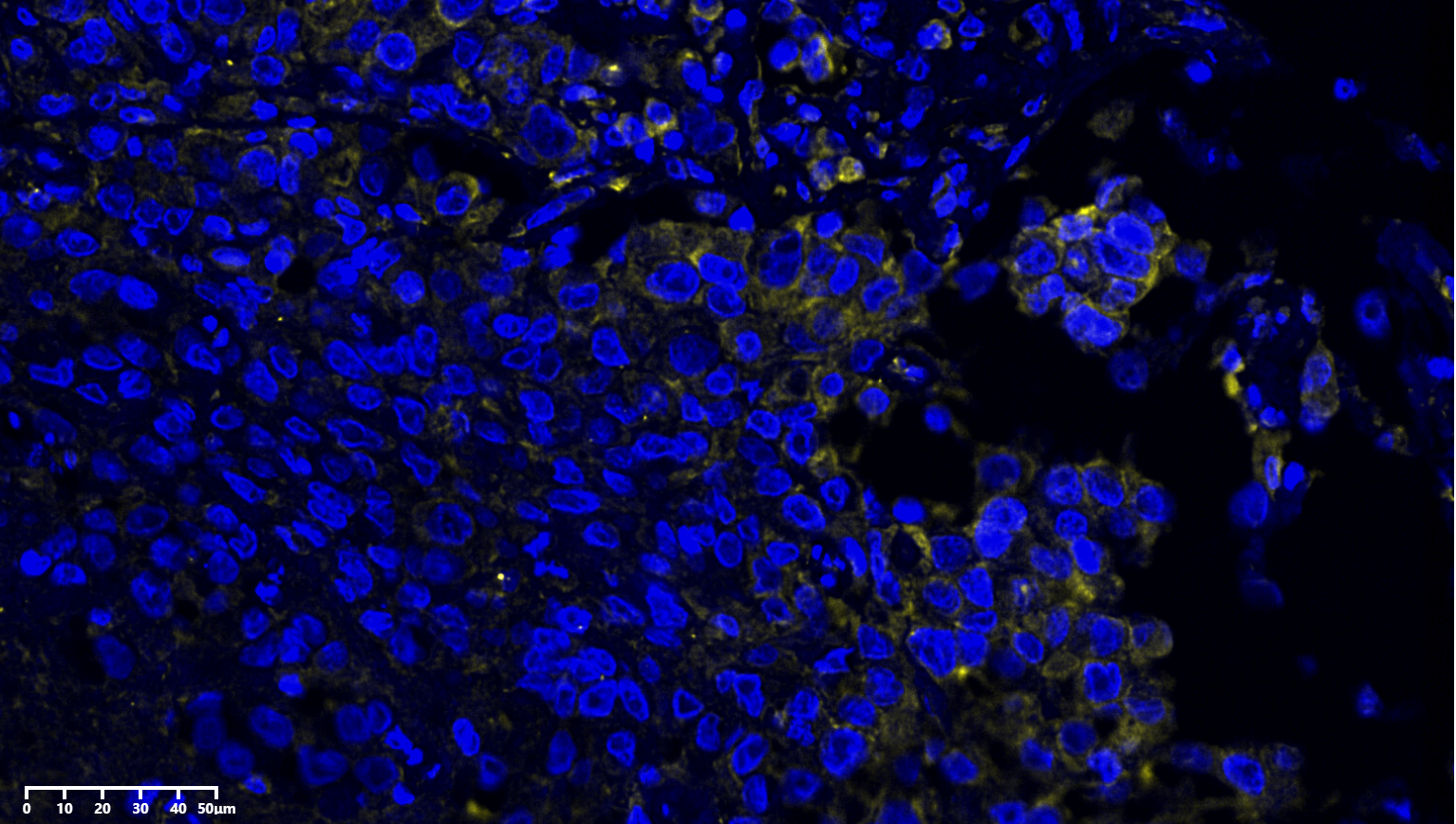

Supplement: Supplementary file 1 [file cancers-18-01384-s001.zip › original images/Microscopy/glioblastoma-1/HGG/ITGA3.tif]

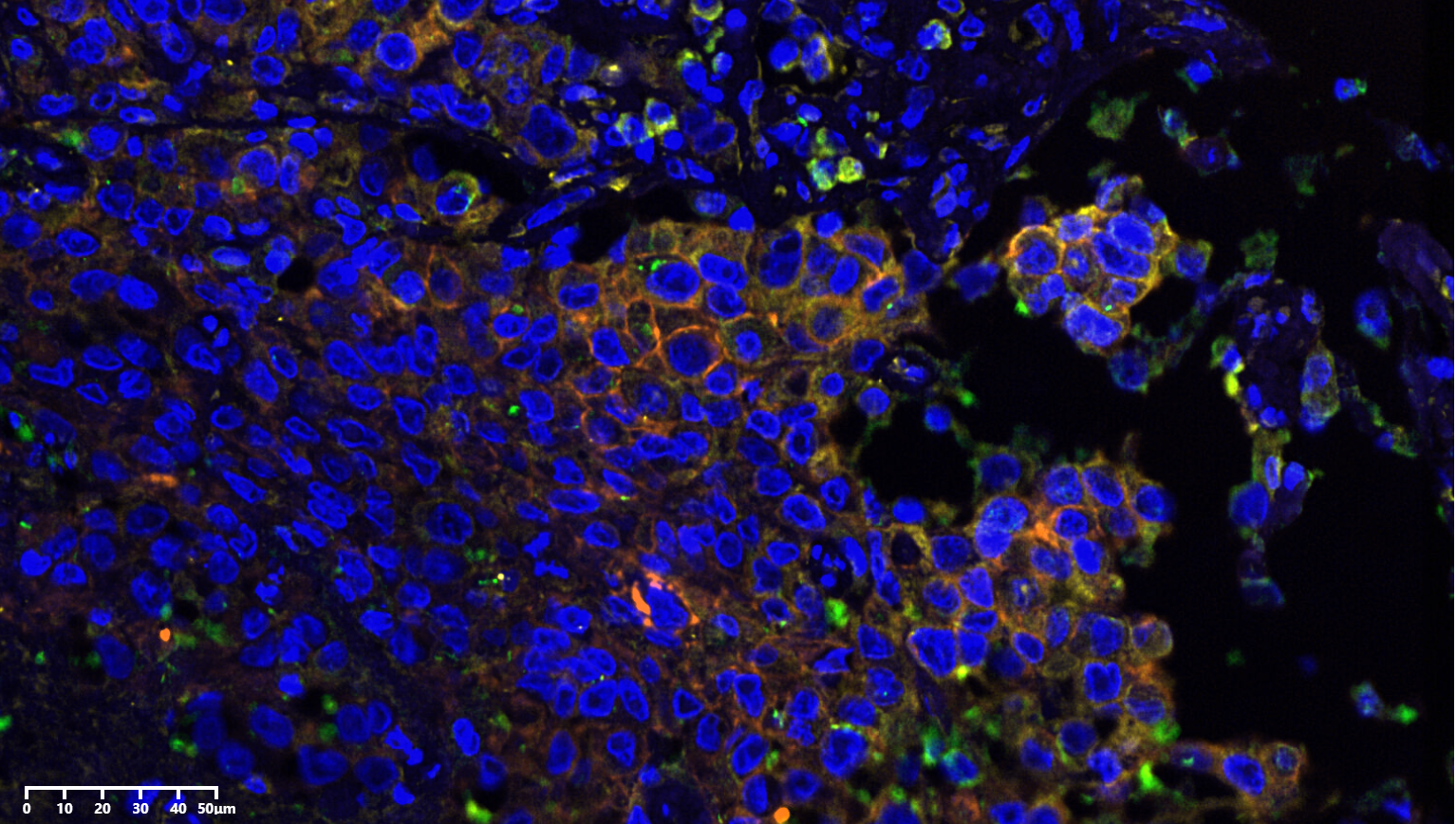

Supplement: Supplementary file 1 [file cancers-18-01384-s001.zip › original images/Microscopy/glioblastoma-1/HGG/MERGE.tif]

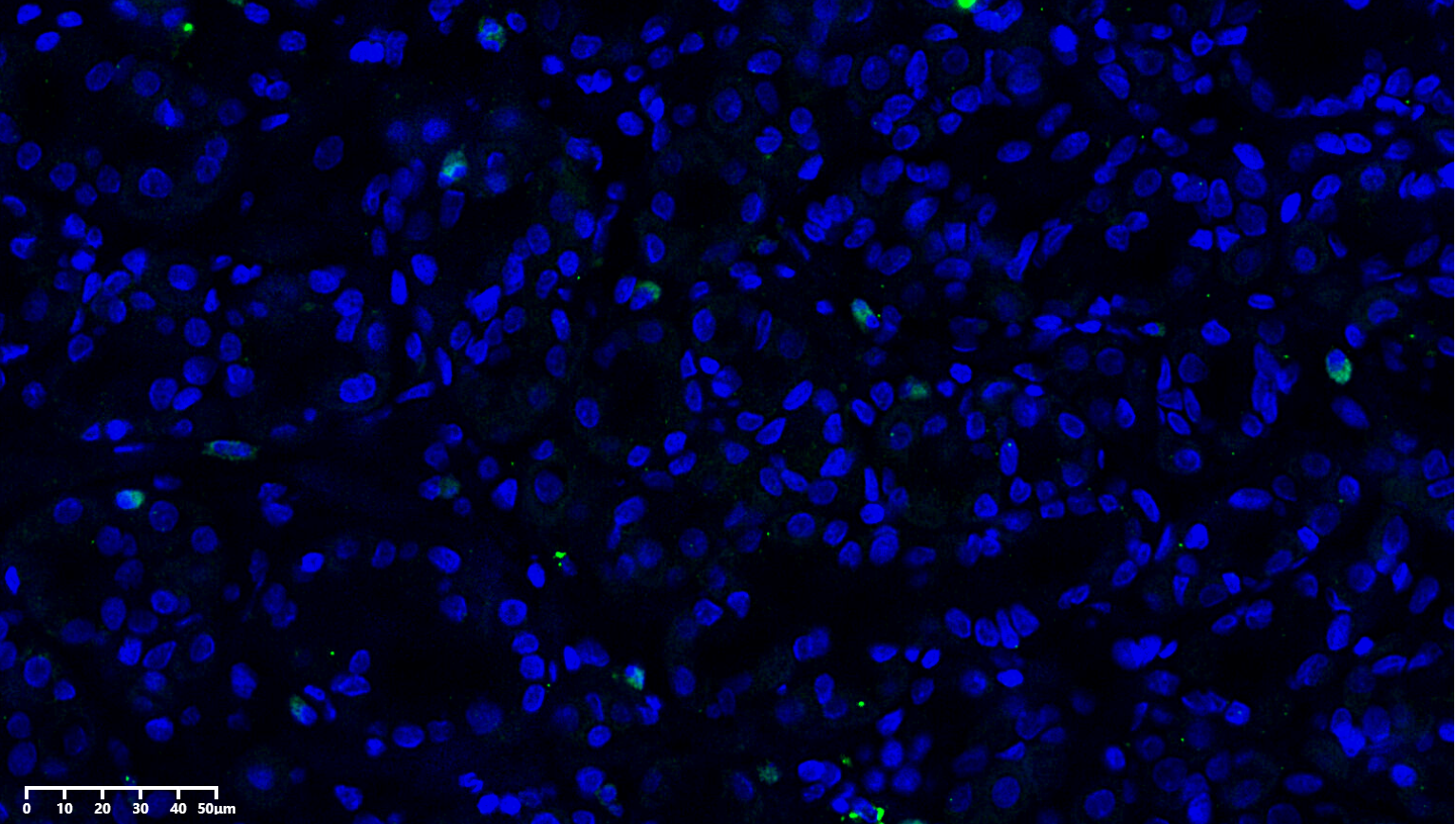

Supplement: Supplementary file 1 [file cancers-18-01384-s001.zip › original images/Microscopy/glioblastoma-1/LGG/7B-01-01.tif]

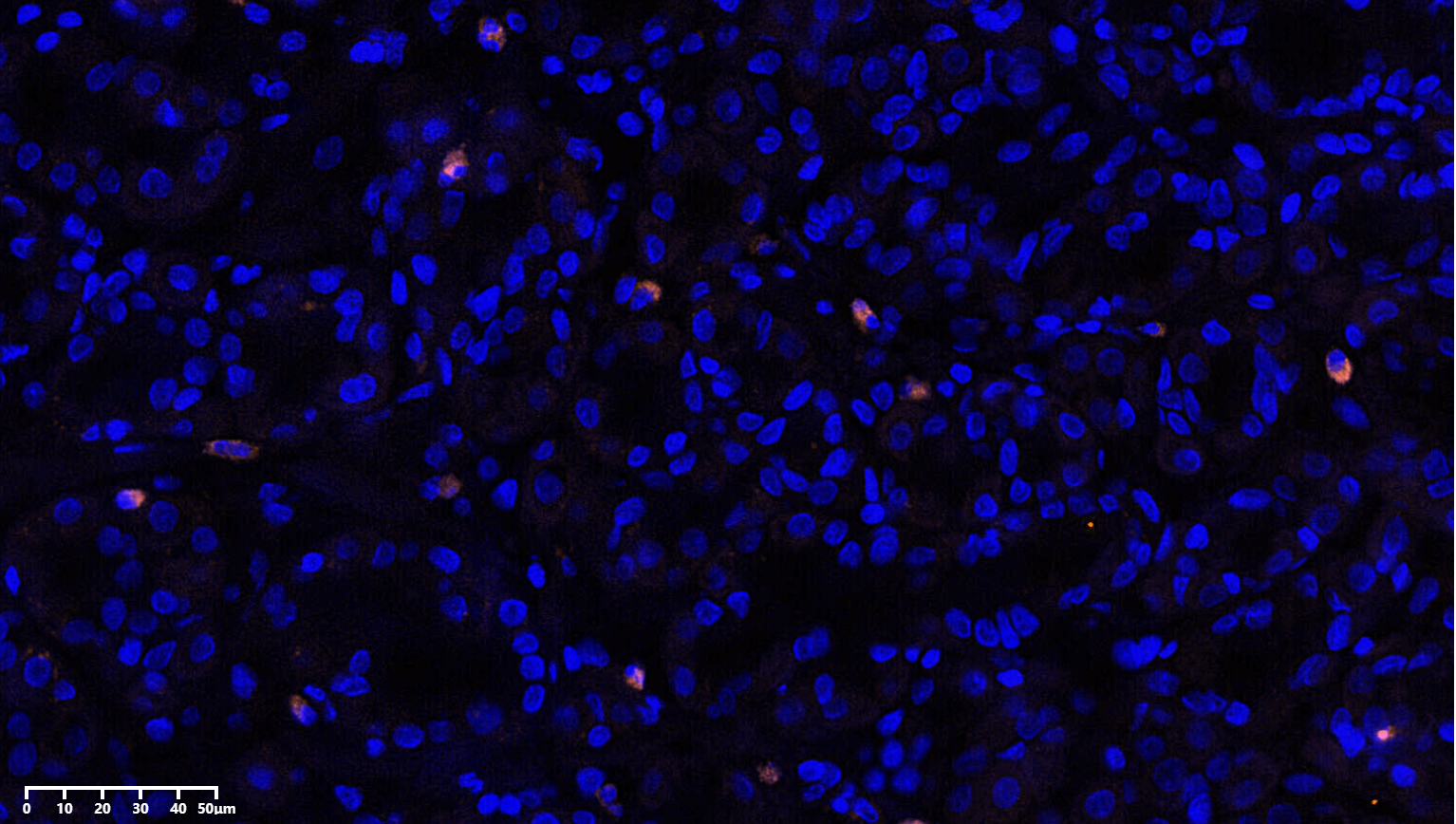

Supplement: Supplementary file 1 [file cancers-18-01384-s001.zip › original images/Microscopy/glioblastoma-1/LGG/EPCAM-01-01-01.tif]

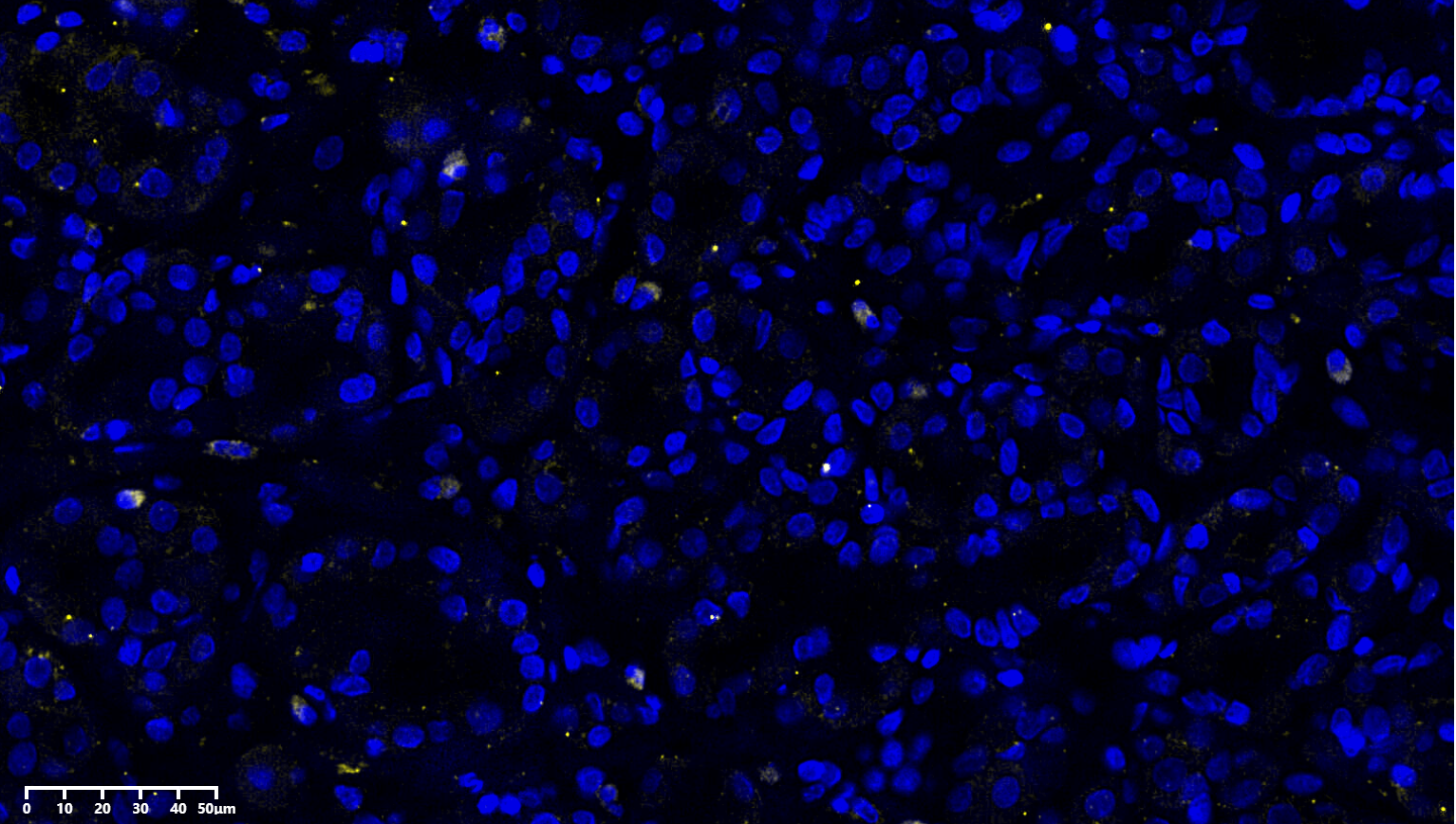

Supplement: Supplementary file 1 [file cancers-18-01384-s001.zip › original images/Microscopy/glioblastoma-1/LGG/ITGA3-01-01.tif]

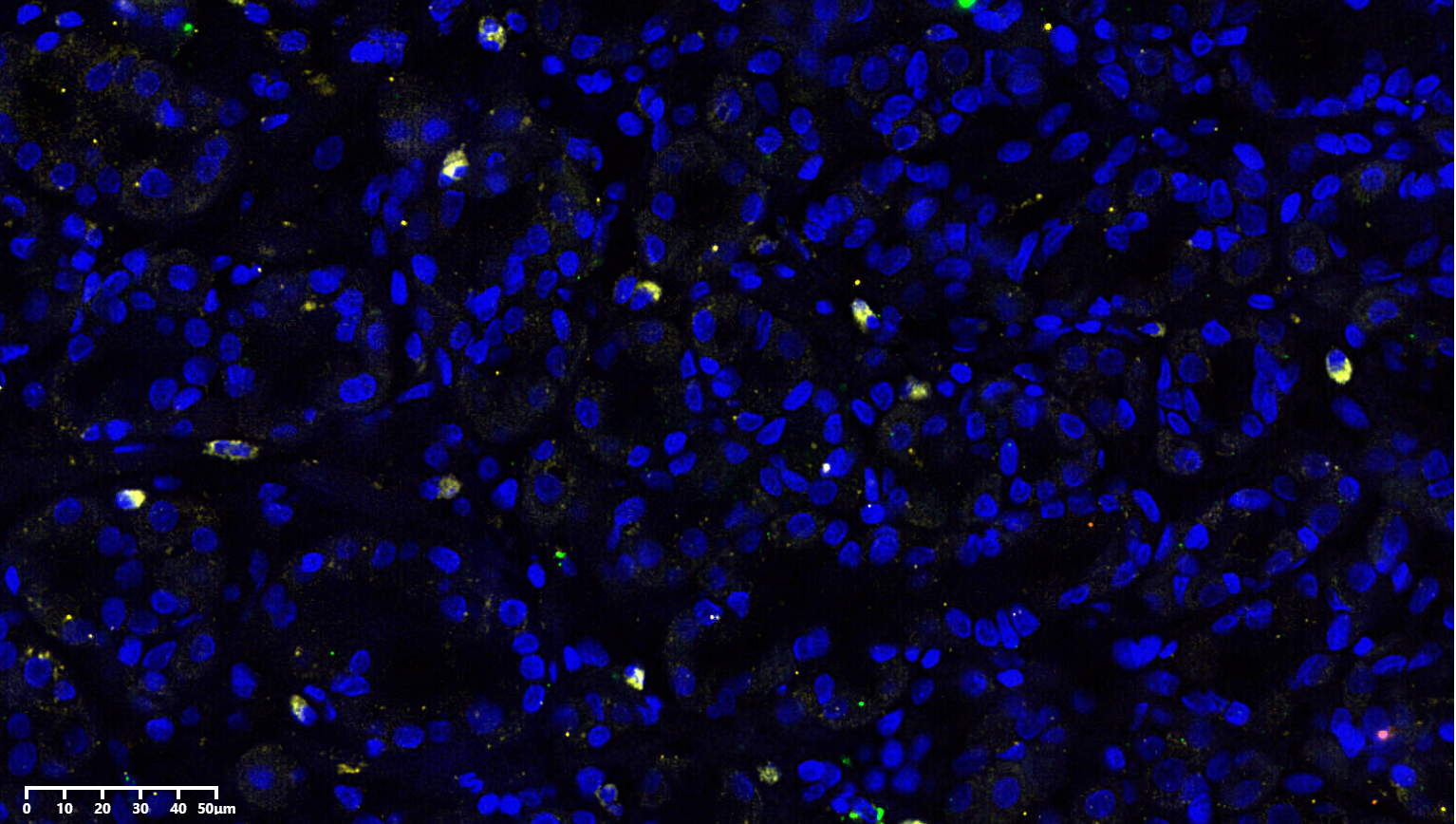

Supplement: Supplementary file 1 [file cancers-18-01384-s001.zip › original images/Microscopy/glioblastoma-1/LGG/MERGE-01-01.tif]

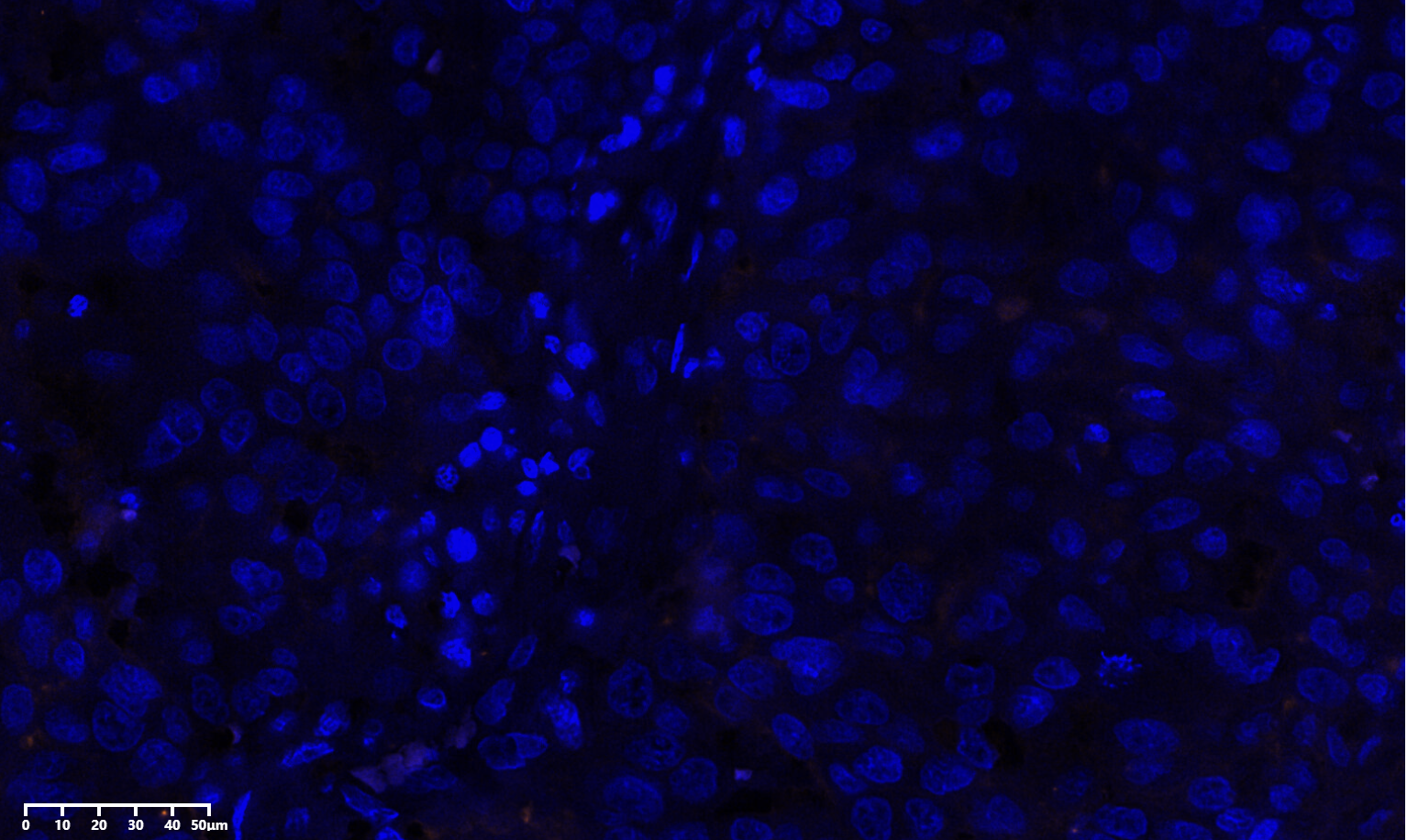

Supplement: Supplementary file 1 [file cancers-18-01384-s001.zip › original images/Microscopy/glioblastoma-1/Paracancerous/EPCAM.tif]

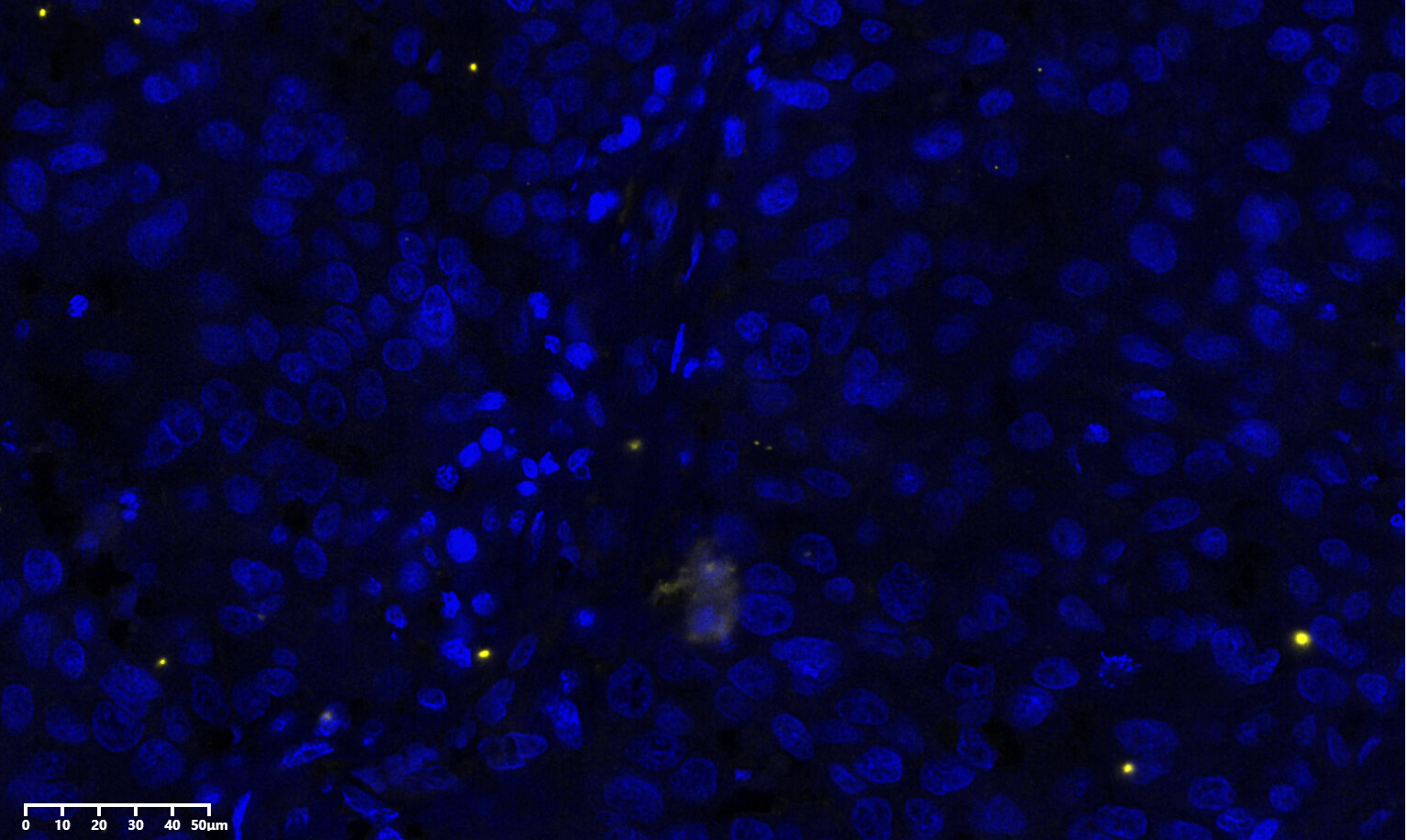

Supplement: Supplementary file 1 [file cancers-18-01384-s001.zip › original images/Microscopy/glioblastoma-1/Paracancerous/ITGA3.tif]

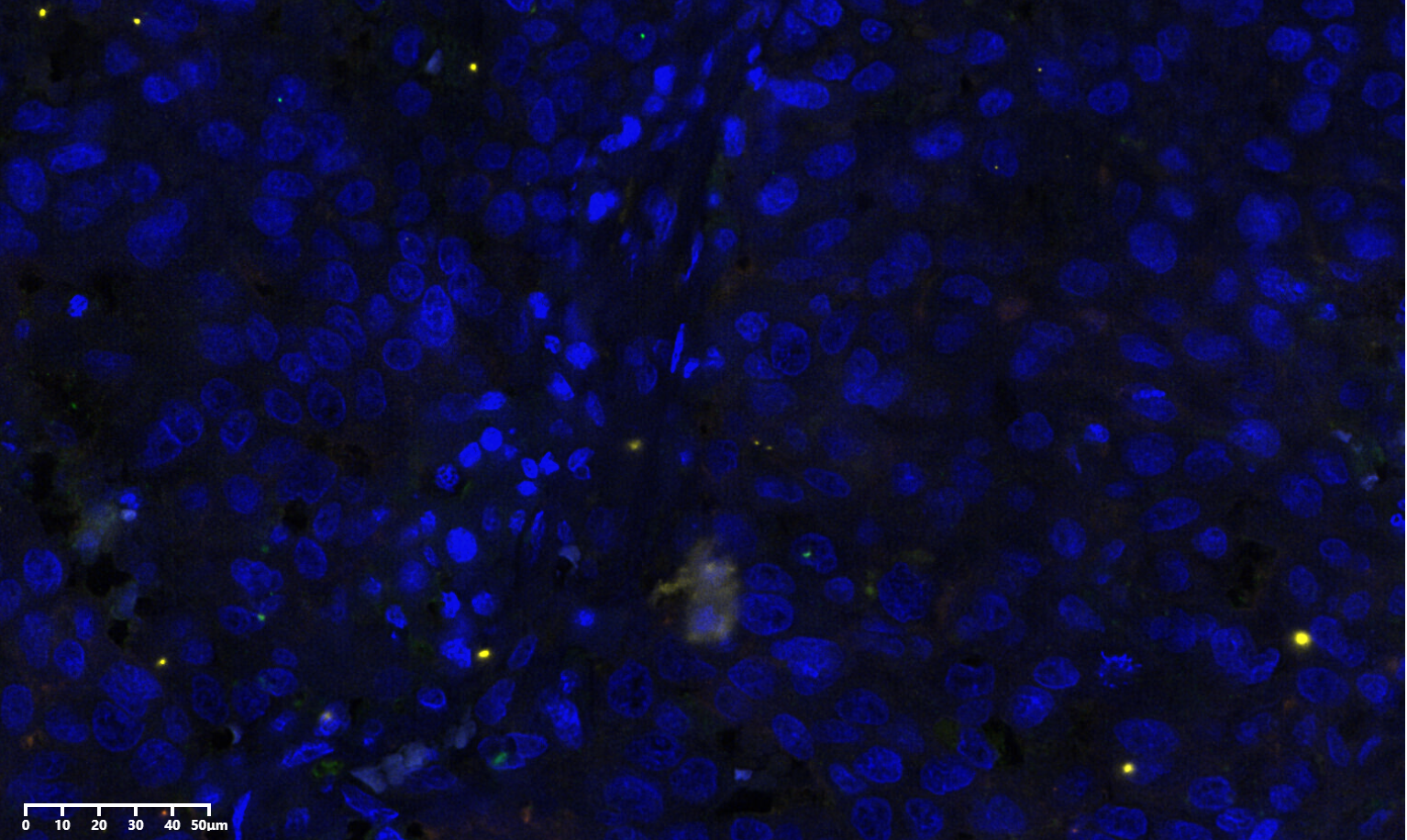

Supplement: Supplementary file 1 [file cancers-18-01384-s001.zip › original images/Microscopy/glioblastoma-1/Paracancerous/MERGE.tif]

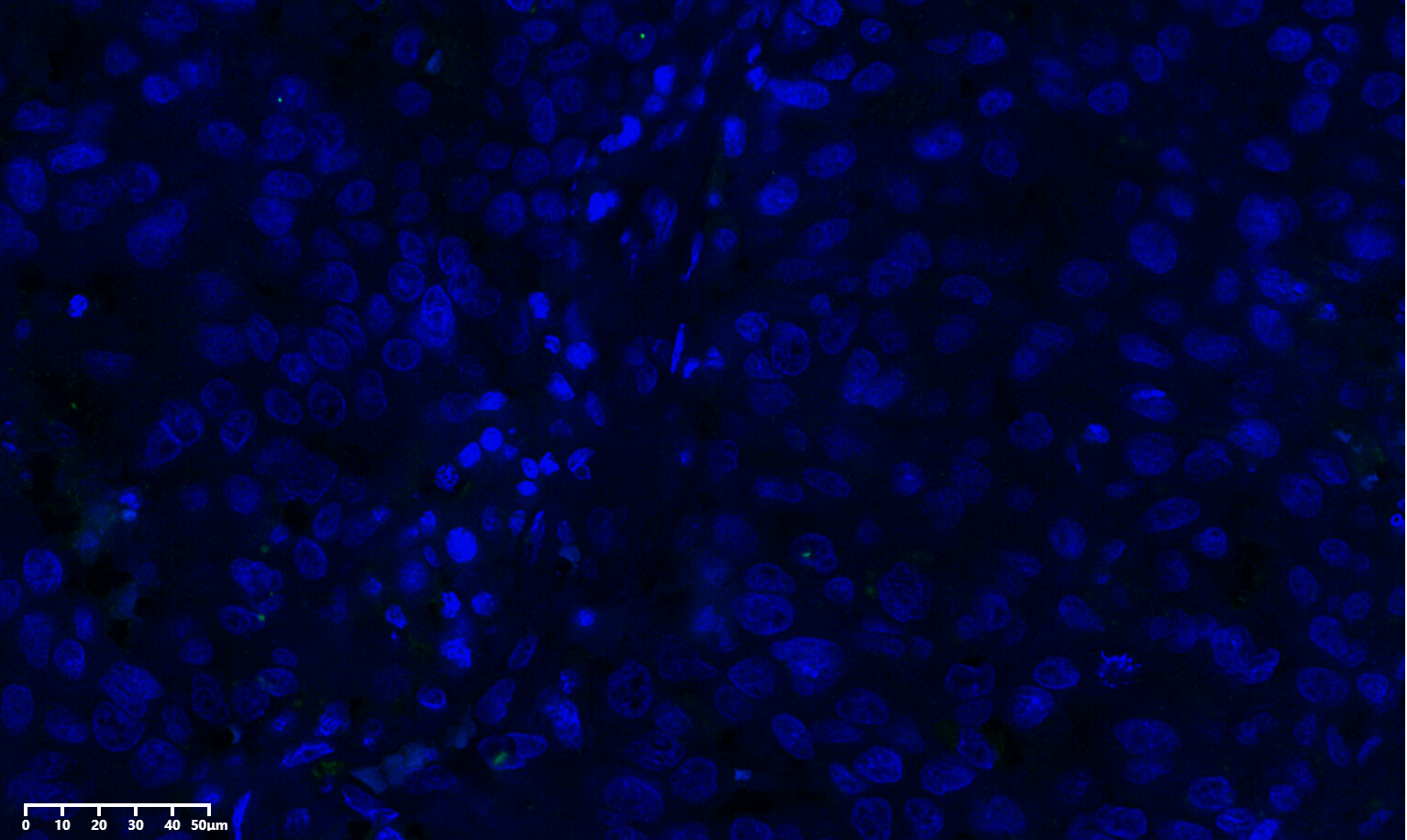

Supplement: Supplementary file 1 [file cancers-18-01384-s001.zip › original images/Microscopy/glioblastoma-1/Paracancerous/METTL7B.tif]

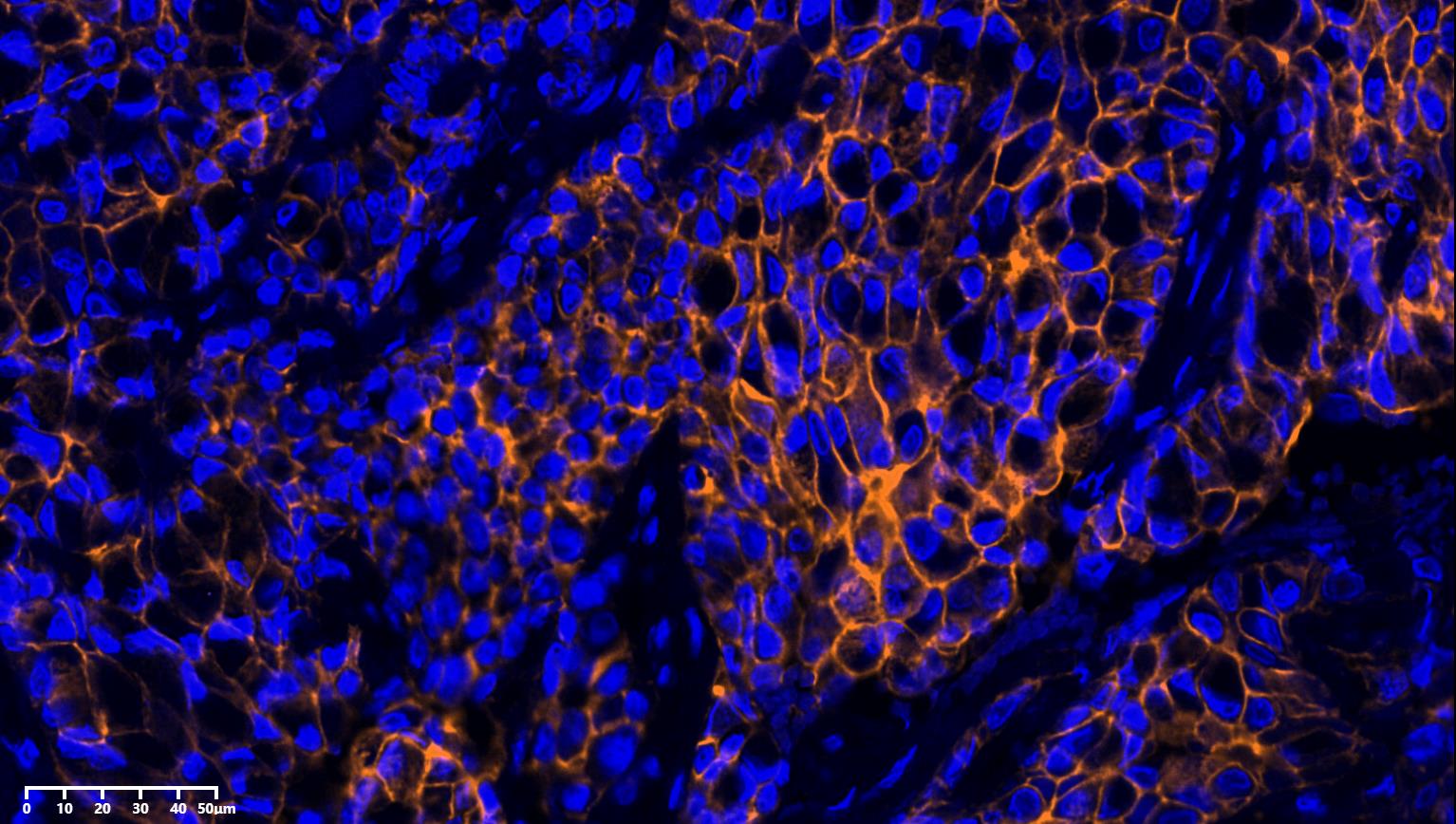

Supplement: Supplementary file 1 [file cancers-18-01384-s001.zip › original images/Microscopy/glioblastoma-2/HGG/EPCAM.jpg]

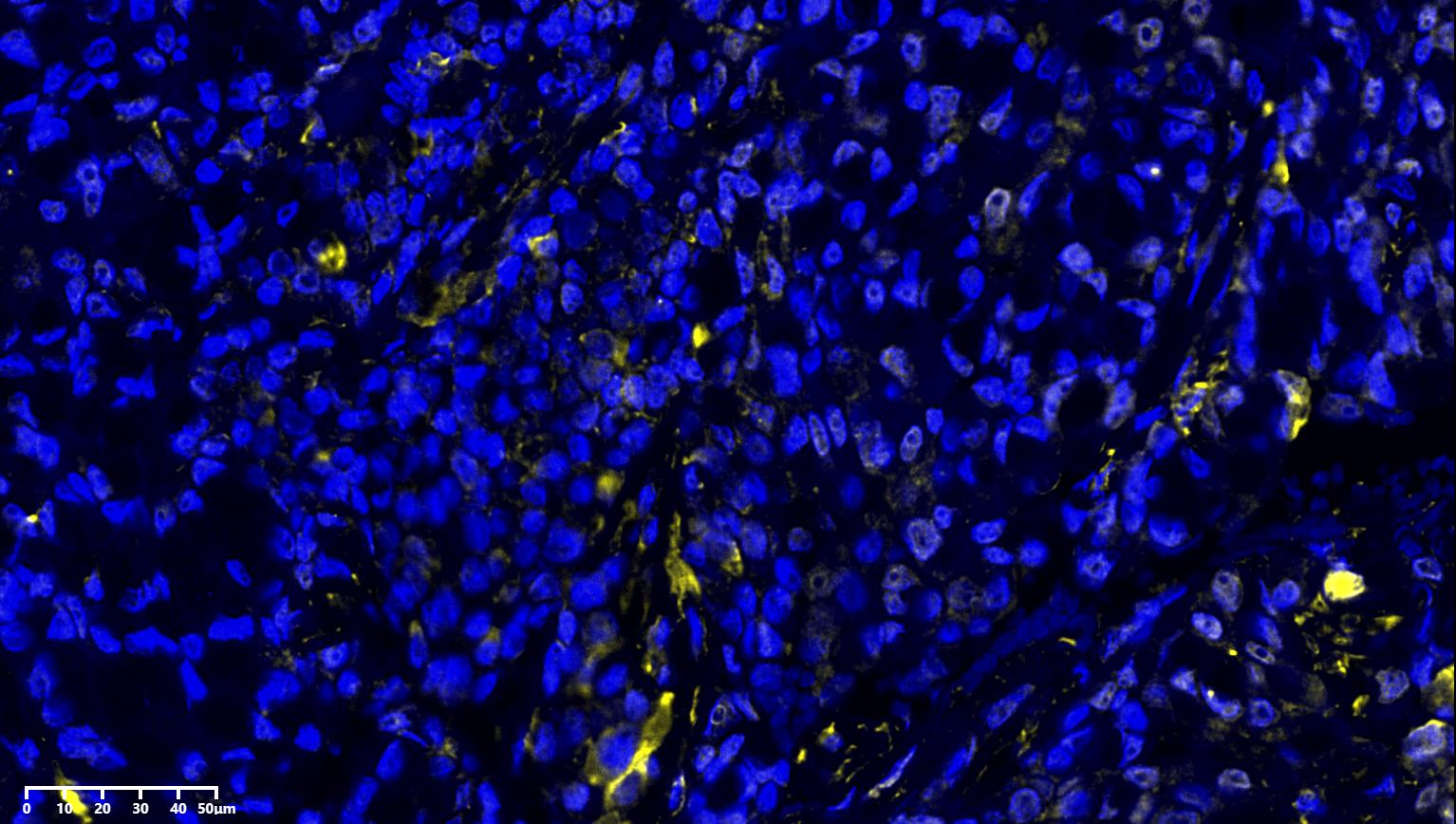

Supplement: Supplementary file 1 [file cancers-18-01384-s001.zip › original images/Microscopy/glioblastoma-2/HGG/ITGA3.jpg]

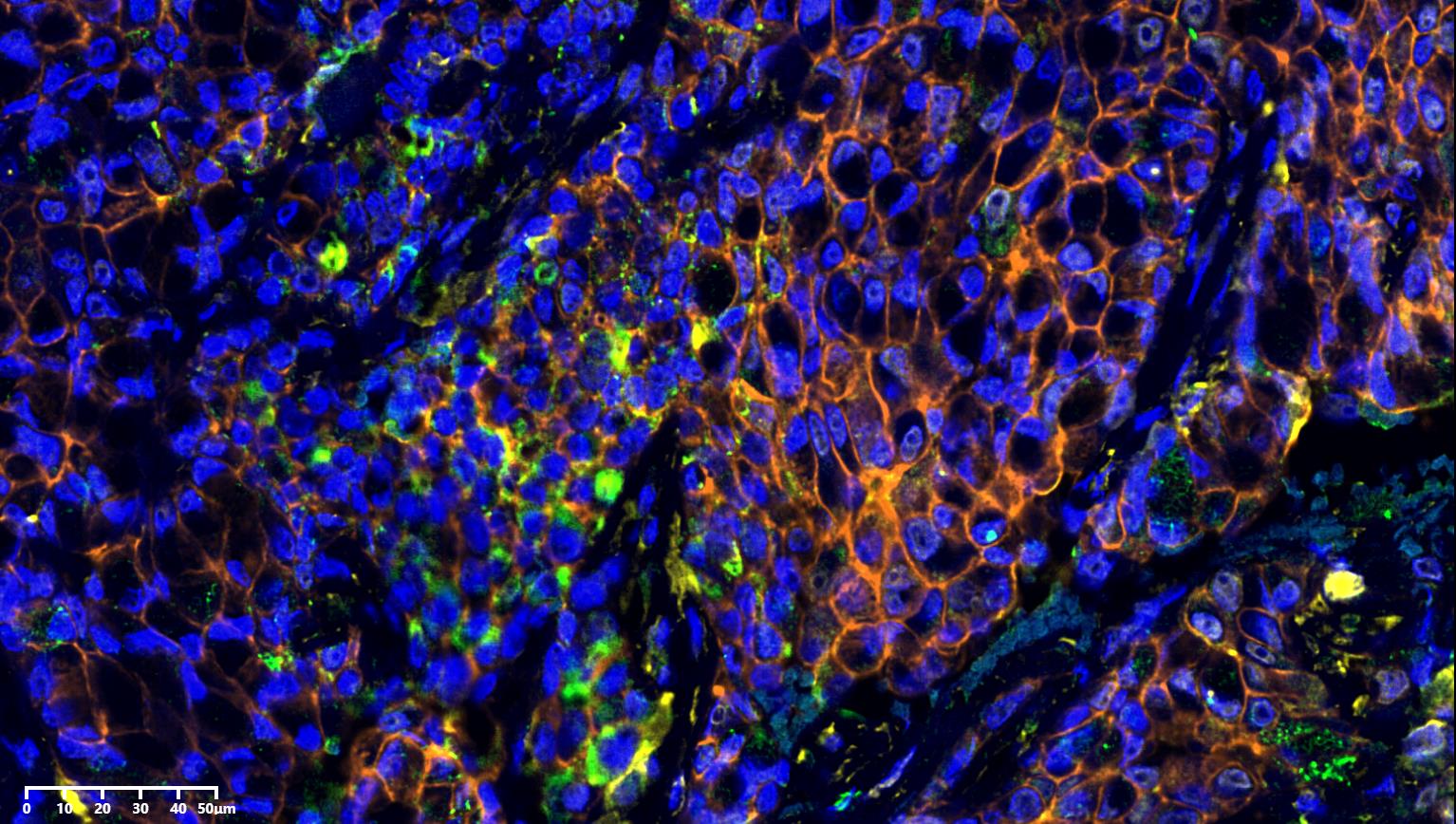

Supplement: Supplementary file 1 [file cancers-18-01384-s001.zip › original images/Microscopy/glioblastoma-2/HGG/MERGE.jpg]

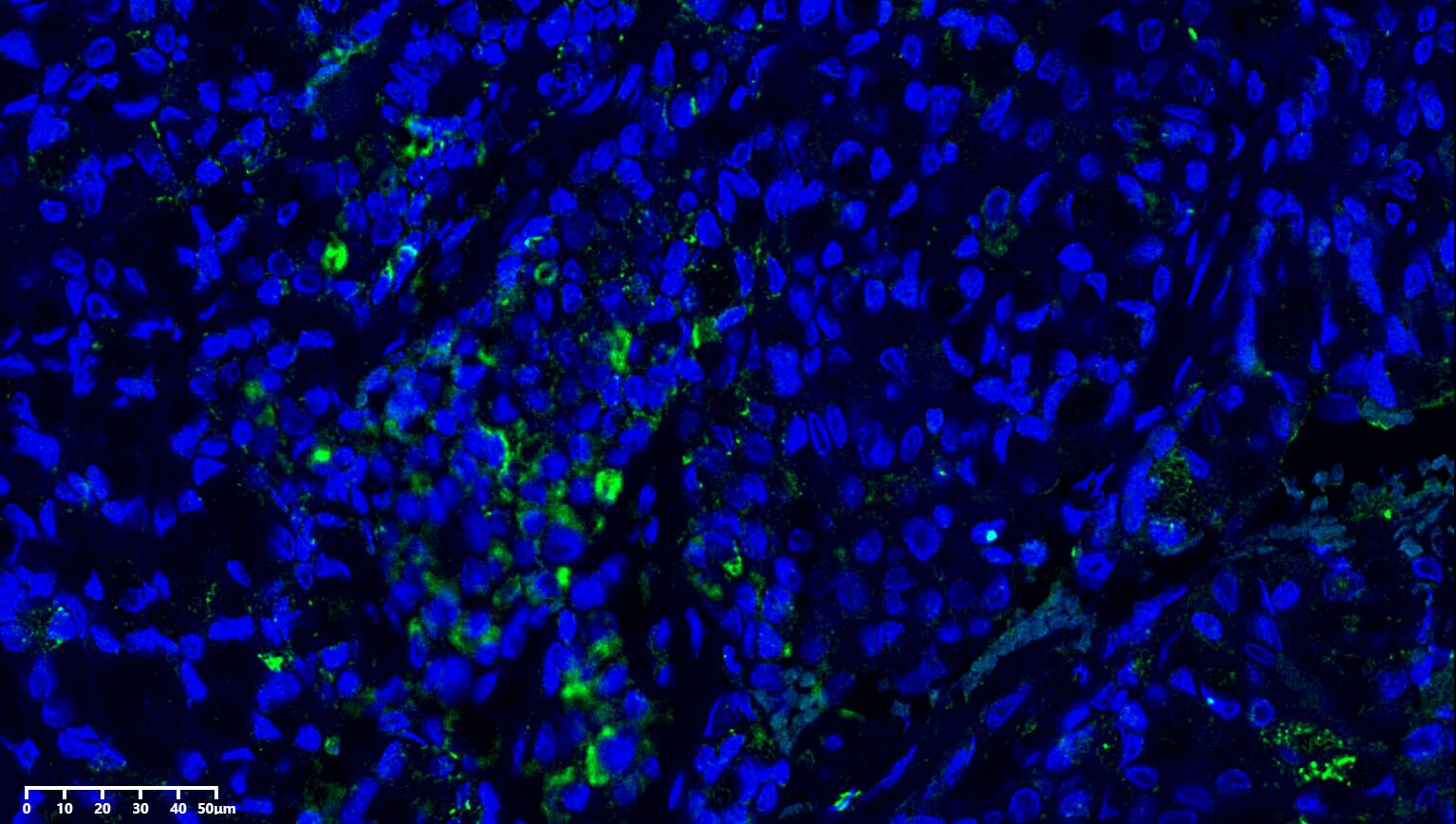

Supplement: Supplementary file 1 [file cancers-18-01384-s001.zip › original images/Microscopy/glioblastoma-2/HGG/METTL7B.jpg]

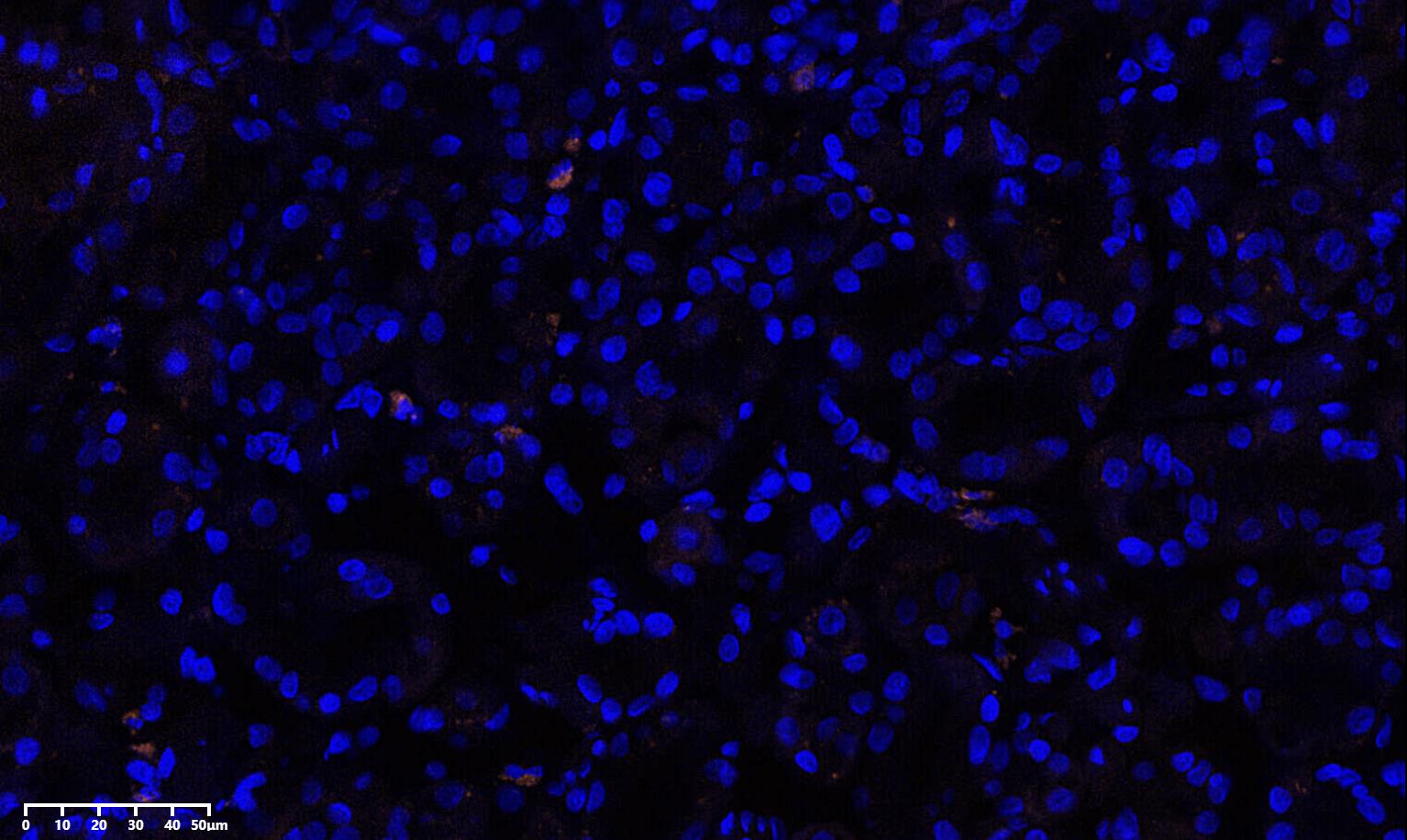

Supplement: Supplementary file 1 [file cancers-18-01384-s001.zip › original images/Microscopy/glioblastoma-2/LGG/EPCAM.jpg]

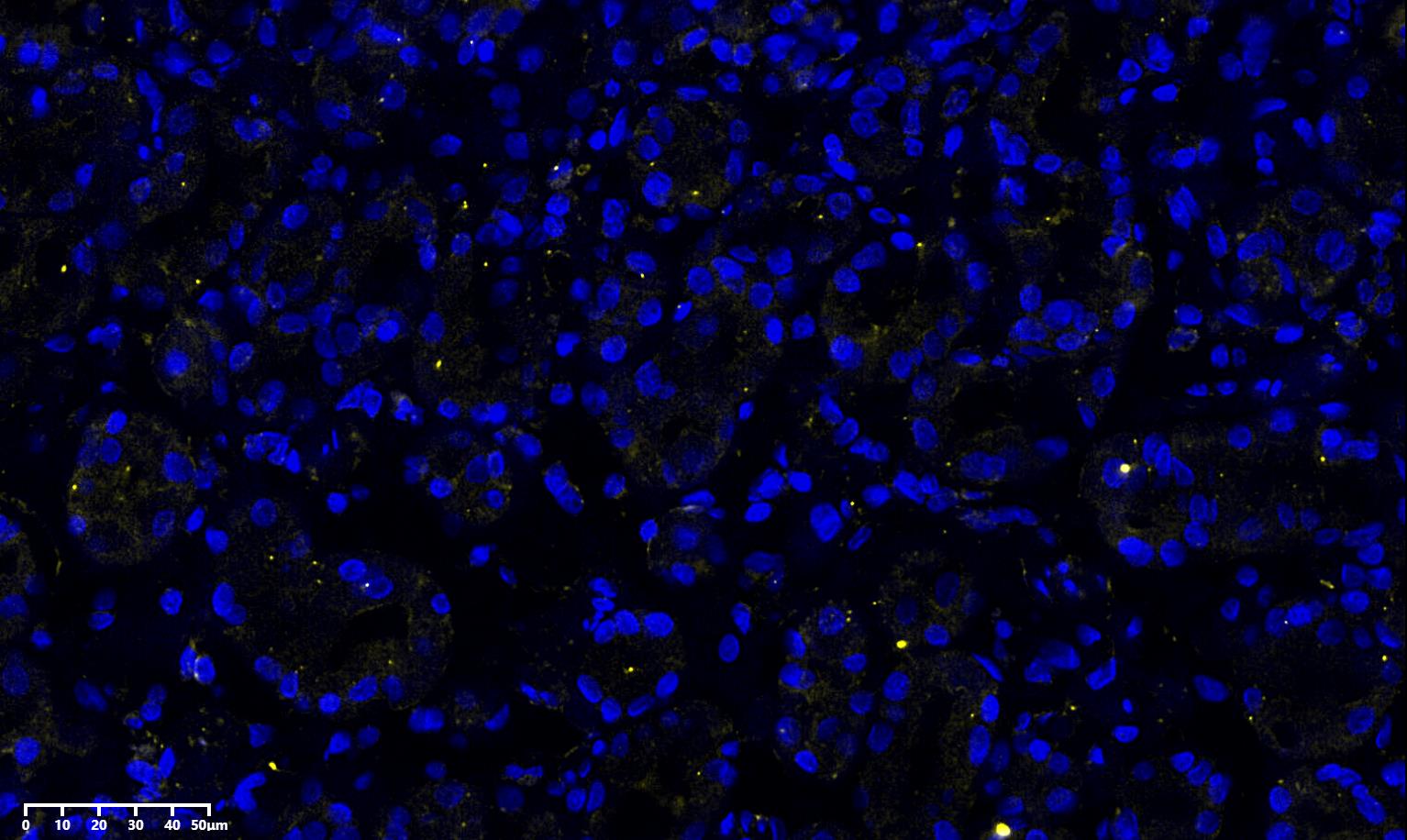

Supplement: Supplementary file 1 [file cancers-18-01384-s001.zip › original images/Microscopy/glioblastoma-2/LGG/ITGA3-01.jpg]

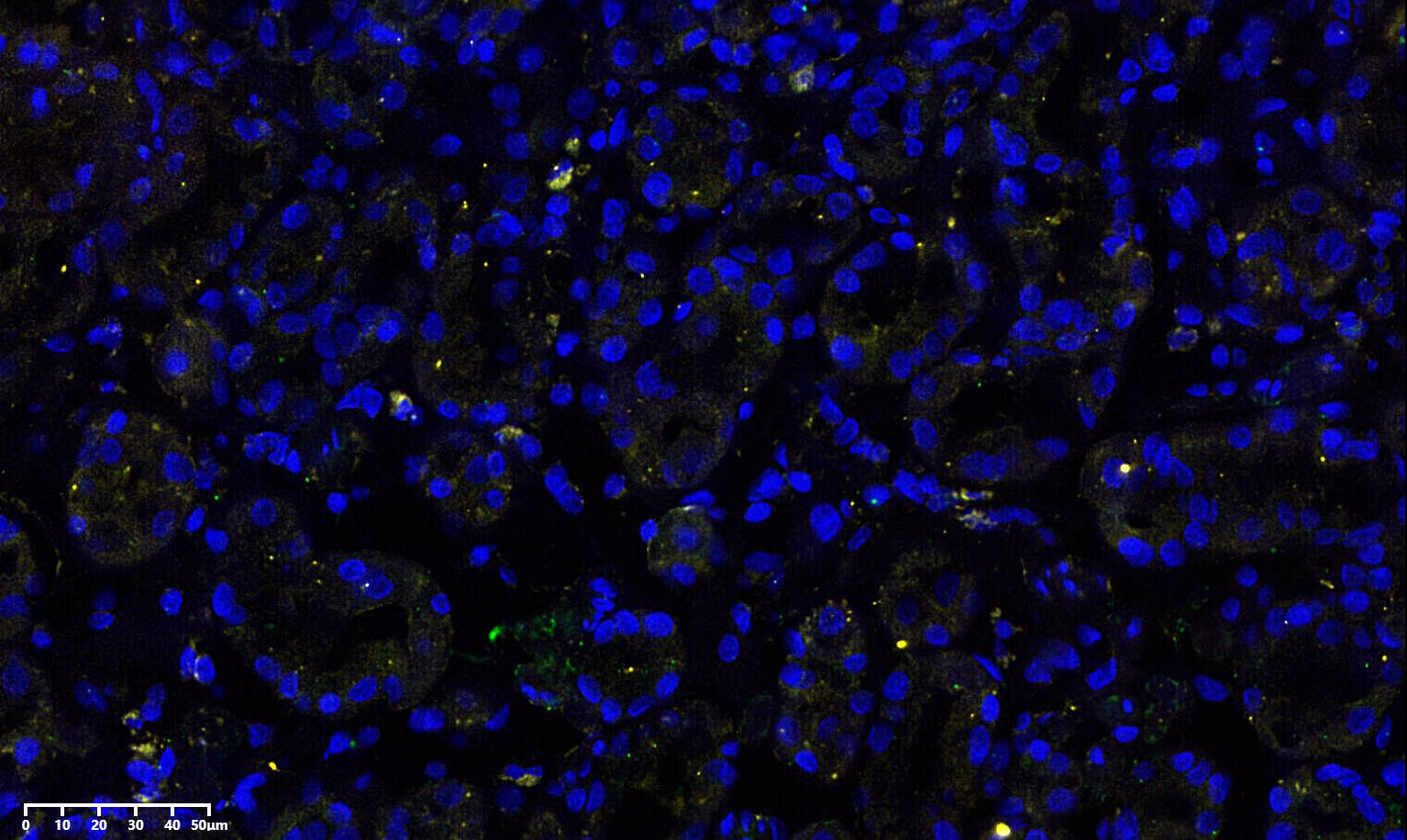

Supplement: Supplementary file 1 [file cancers-18-01384-s001.zip › original images/Microscopy/glioblastoma-2/LGG/MERGE.jpg]

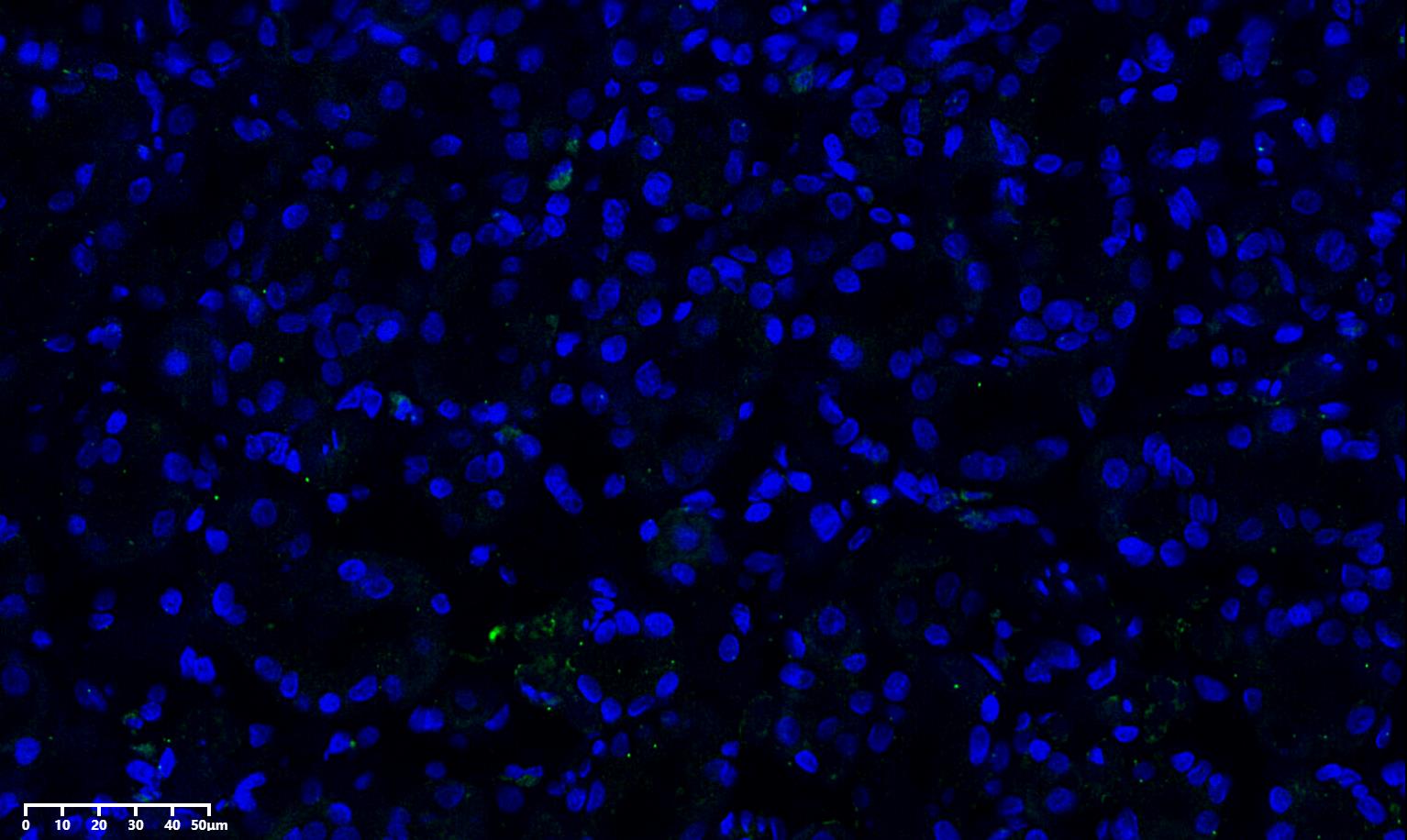

Supplement: Supplementary file 1 [file cancers-18-01384-s001.zip › original images/Microscopy/glioblastoma-2/LGG/METTL7B.jpg]

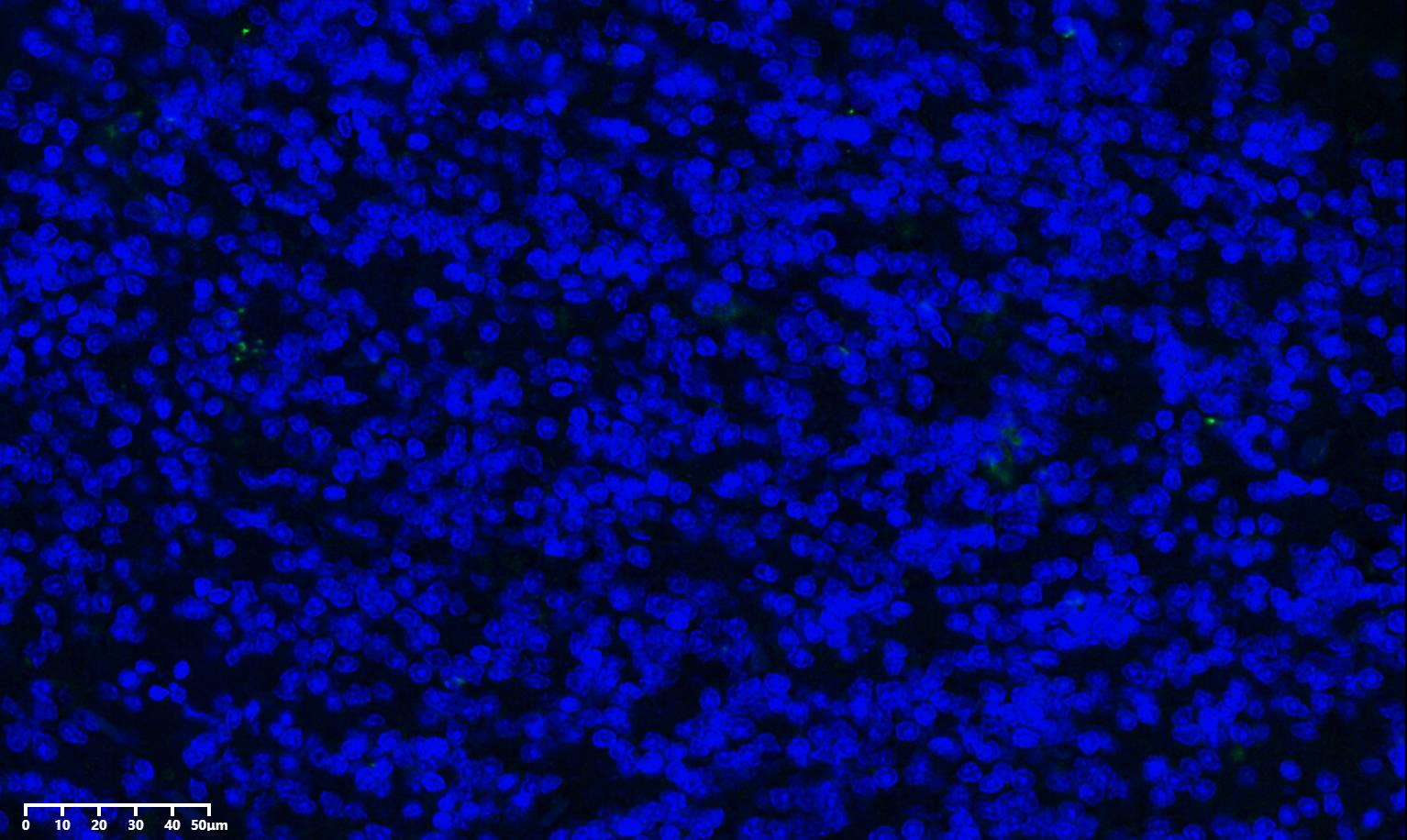

Supplement: Supplementary file 1 [file cancers-18-01384-s001.zip › original images/Microscopy/glioblastoma-2/Paracancerous/EPCAM.jpg]

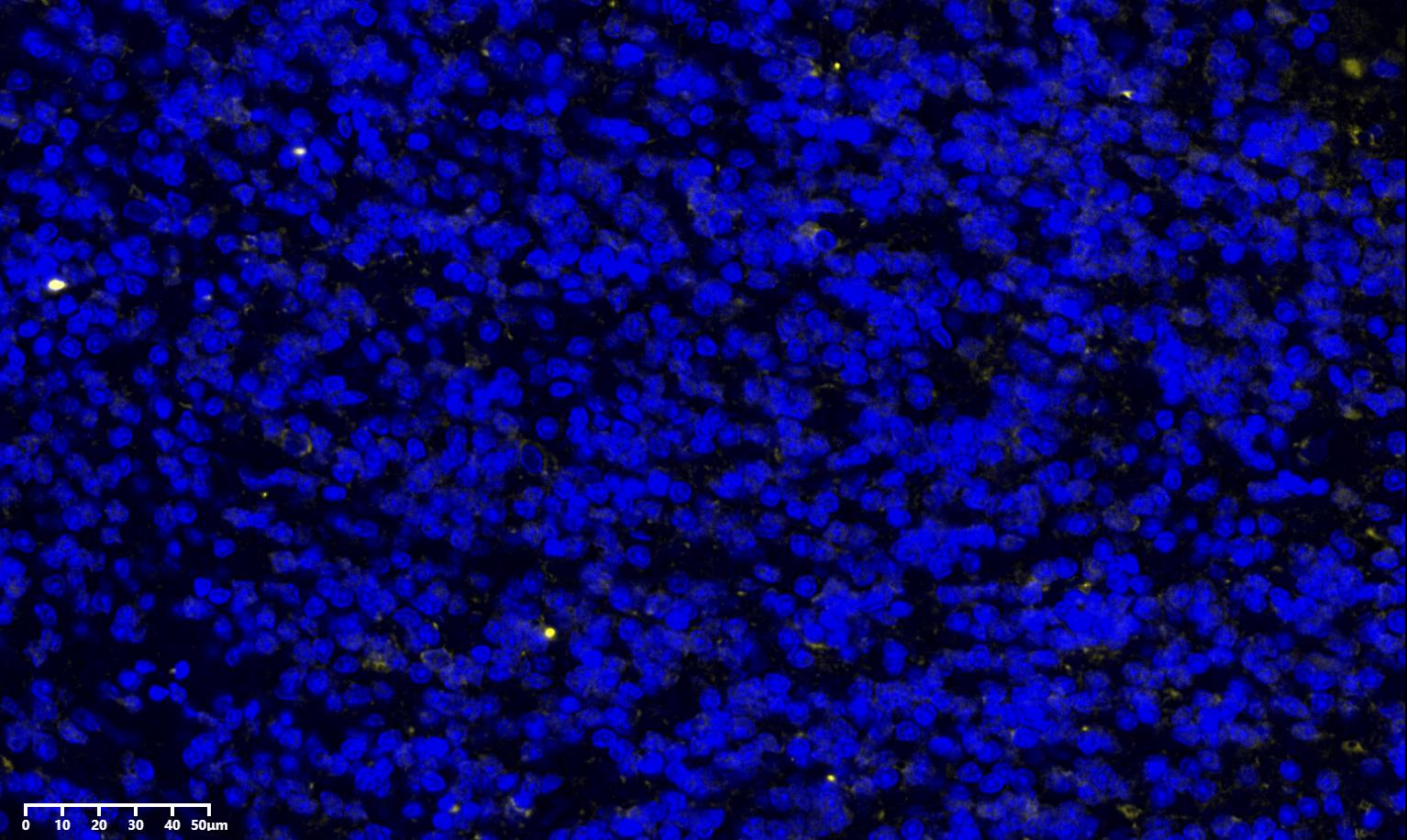

Supplement: Supplementary file 1 [file cancers-18-01384-s001.zip › original images/Microscopy/glioblastoma-2/Paracancerous/ITGA3.jpg]

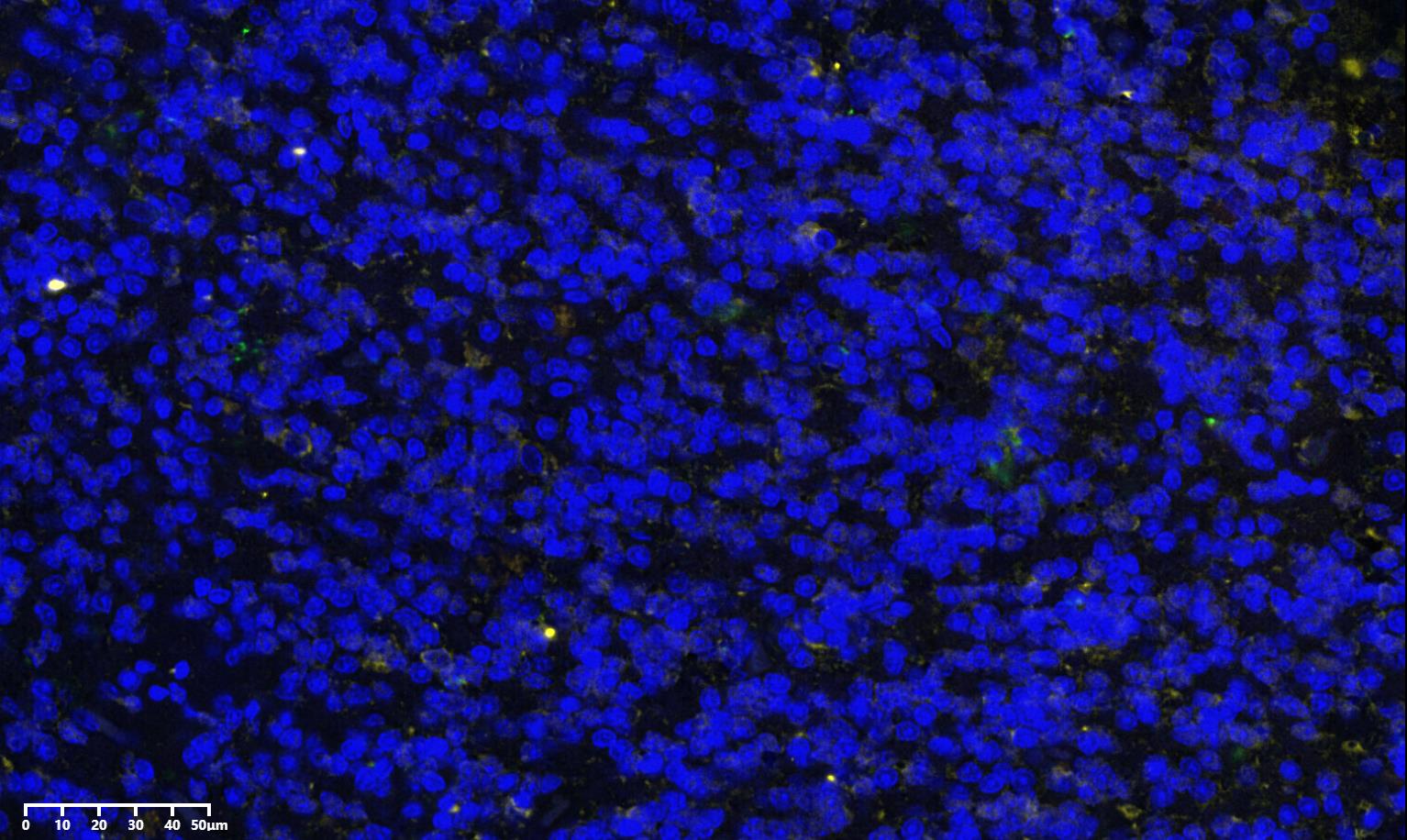

Supplement: Supplementary file 1 [file cancers-18-01384-s001.zip › original images/Microscopy/glioblastoma-2/Paracancerous/MERGE.jpg]

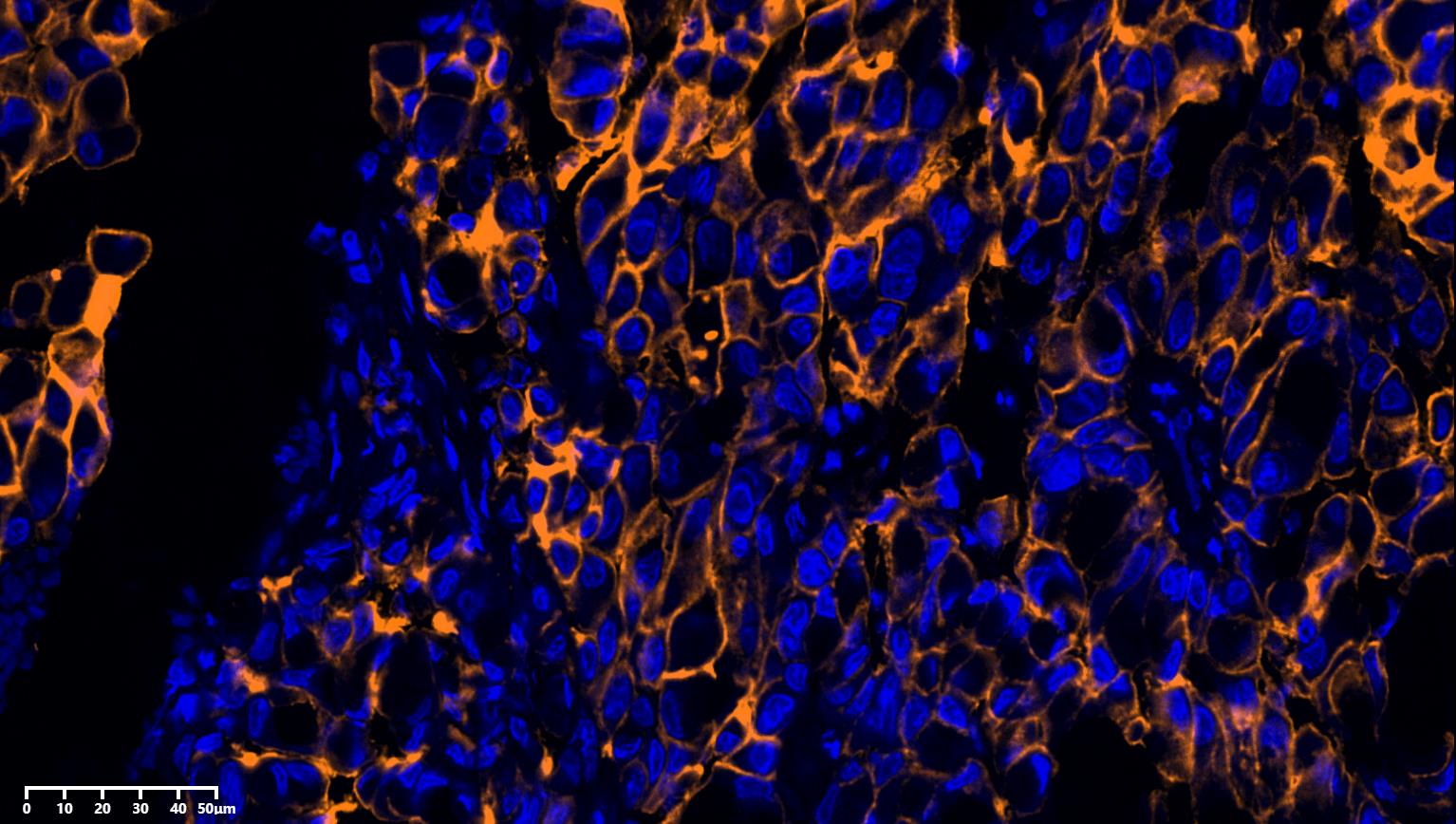

Supplement: Supplementary file 1 [file cancers-18-01384-s001.zip › original images/Microscopy/glioblastoma-3/HGG/EPCAM.jpg]

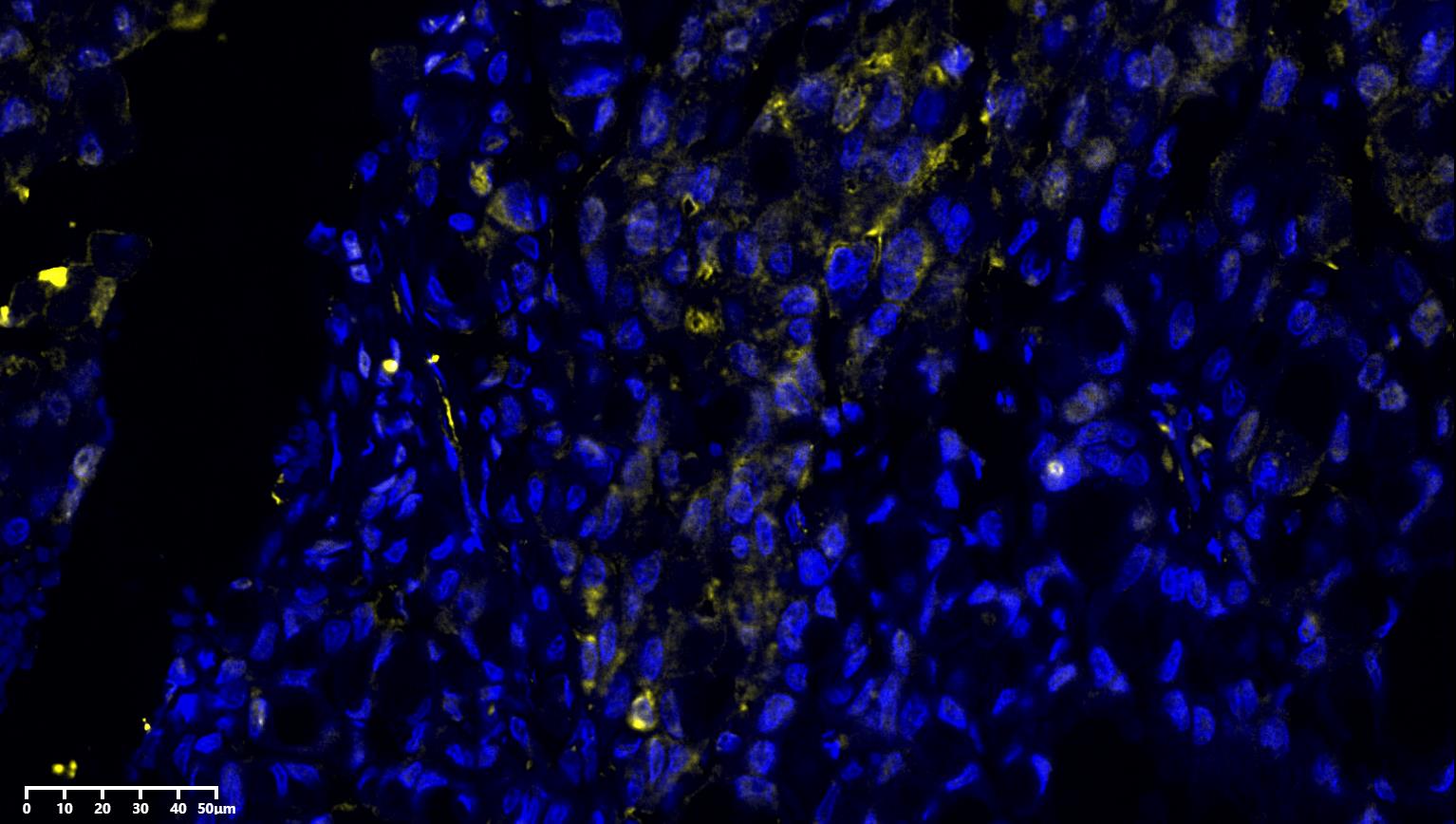

Supplement: Supplementary file 1 [file cancers-18-01384-s001.zip › original images/Microscopy/glioblastoma-3/HGG/ITGA3.jpg]

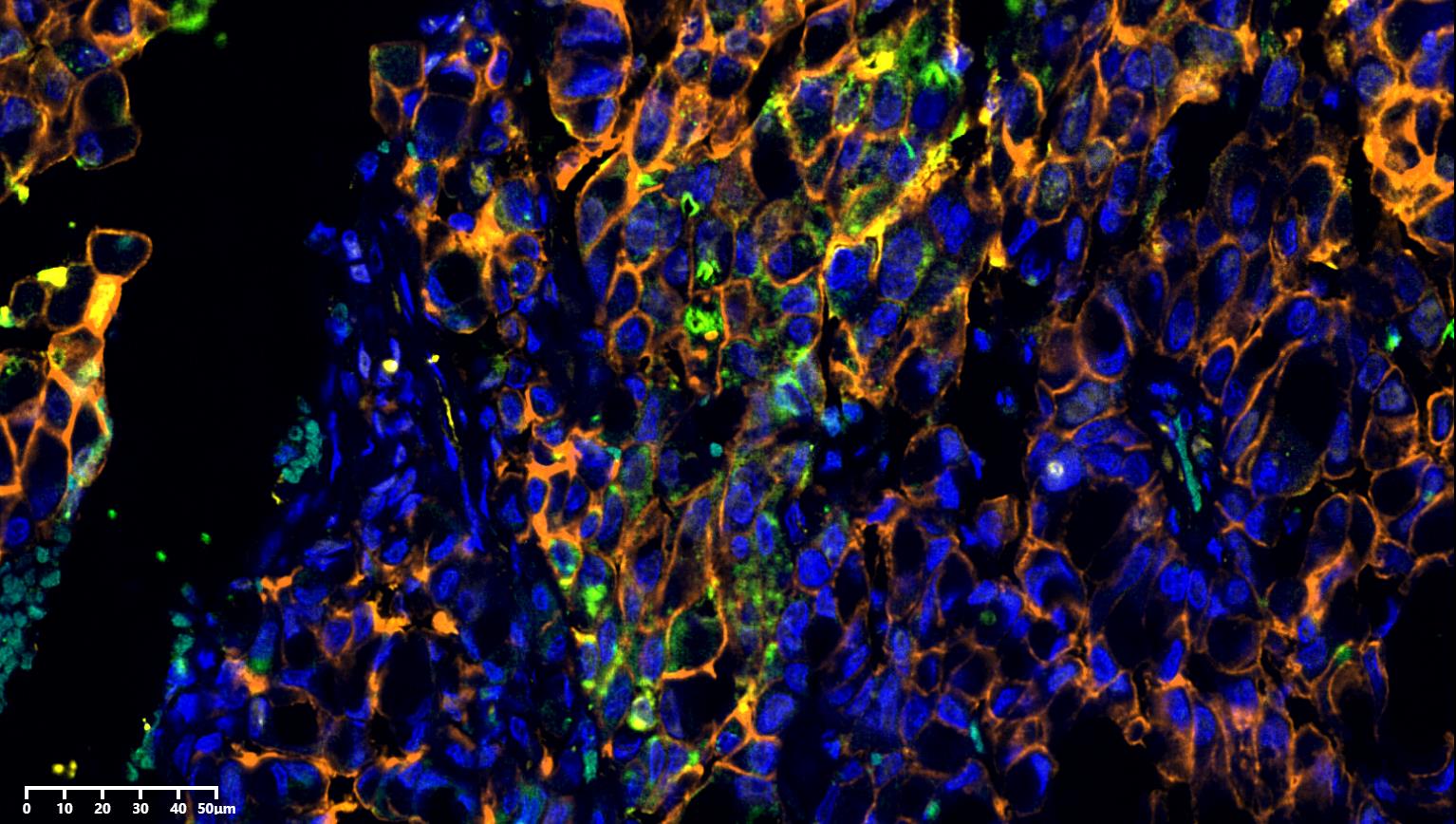

Supplement: Supplementary file 1 [file cancers-18-01384-s001.zip › original images/Microscopy/glioblastoma-3/HGG/MERGE.jpg]

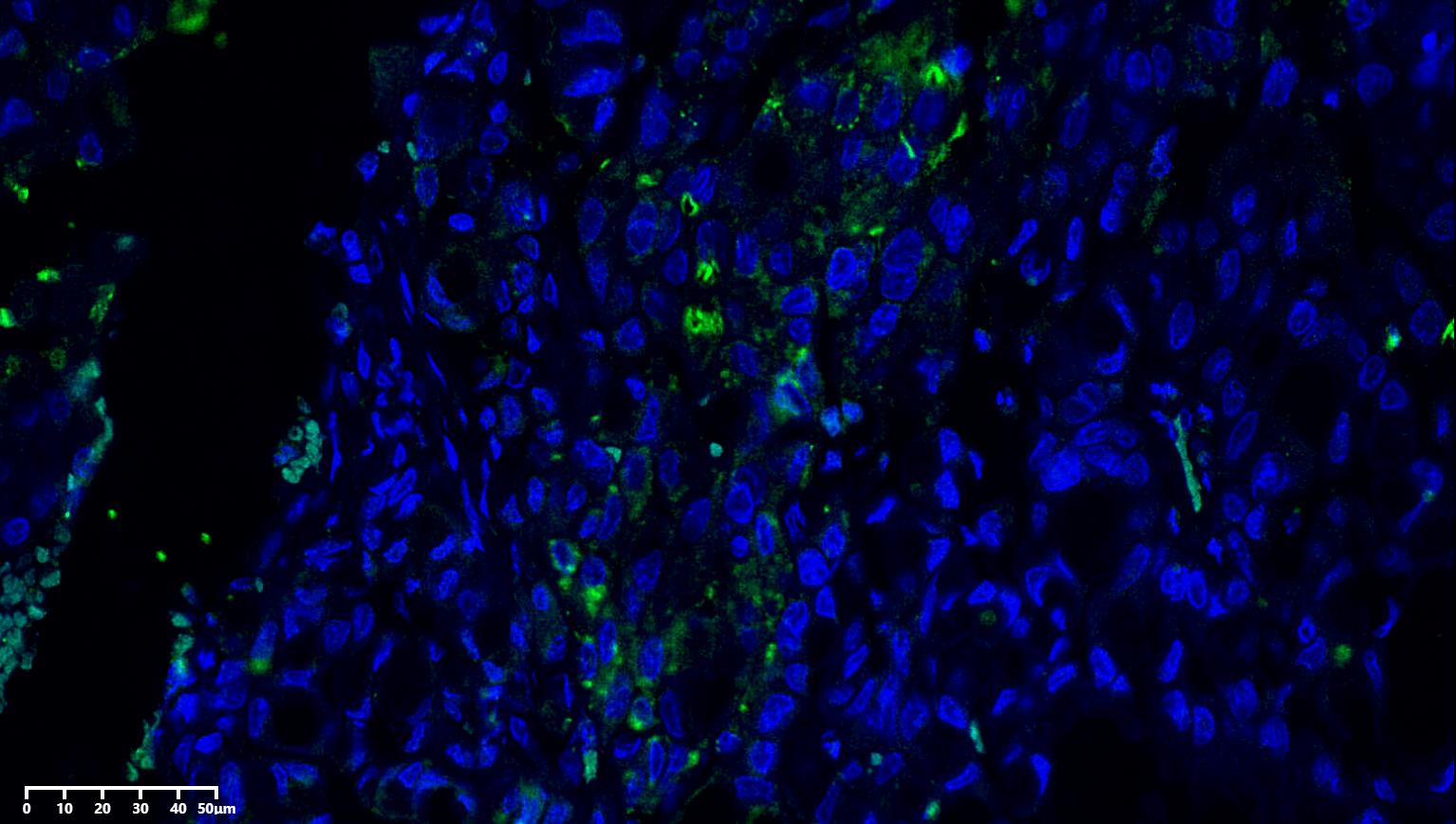

Supplement: Supplementary file 1 [file cancers-18-01384-s001.zip › original images/Microscopy/glioblastoma-3/HGG/METTL7B.jpg]

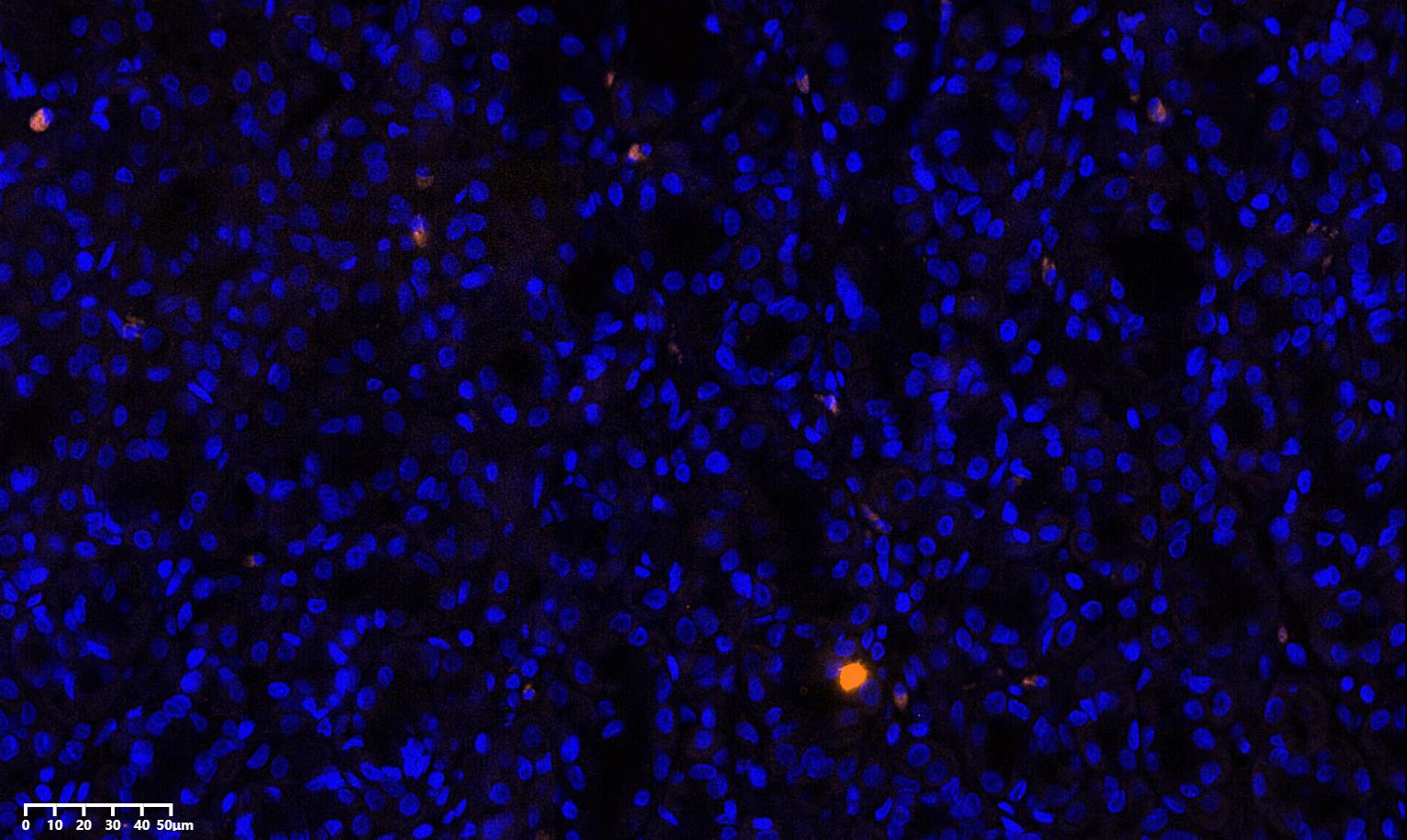

Supplement: Supplementary file 1 [file cancers-18-01384-s001.zip › original images/Microscopy/glioblastoma-3/LGG/EPCAM.jpg]

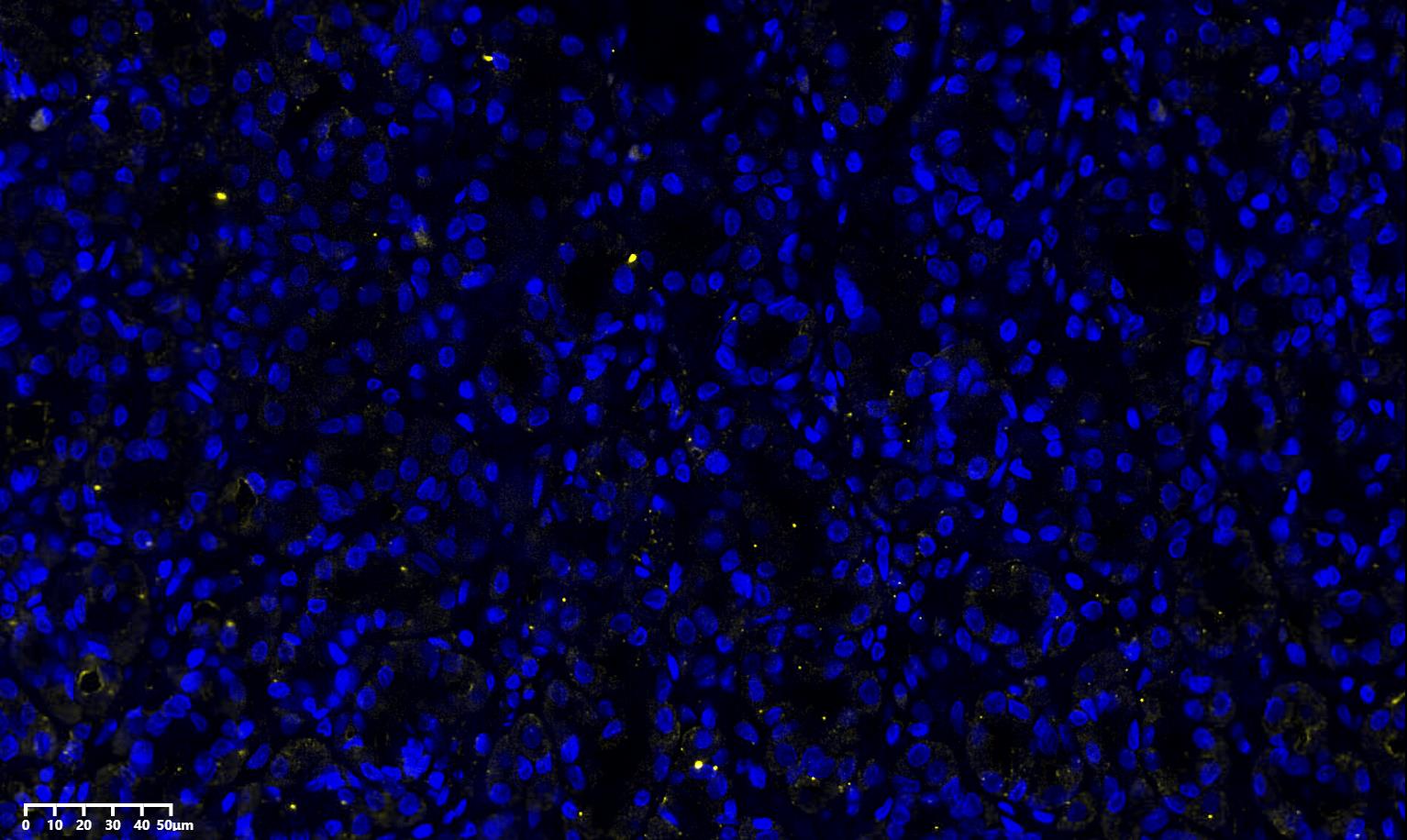

Supplement: Supplementary file 1 [file cancers-18-01384-s001.zip › original images/Microscopy/glioblastoma-3/LGG/ITGA3.jpg]

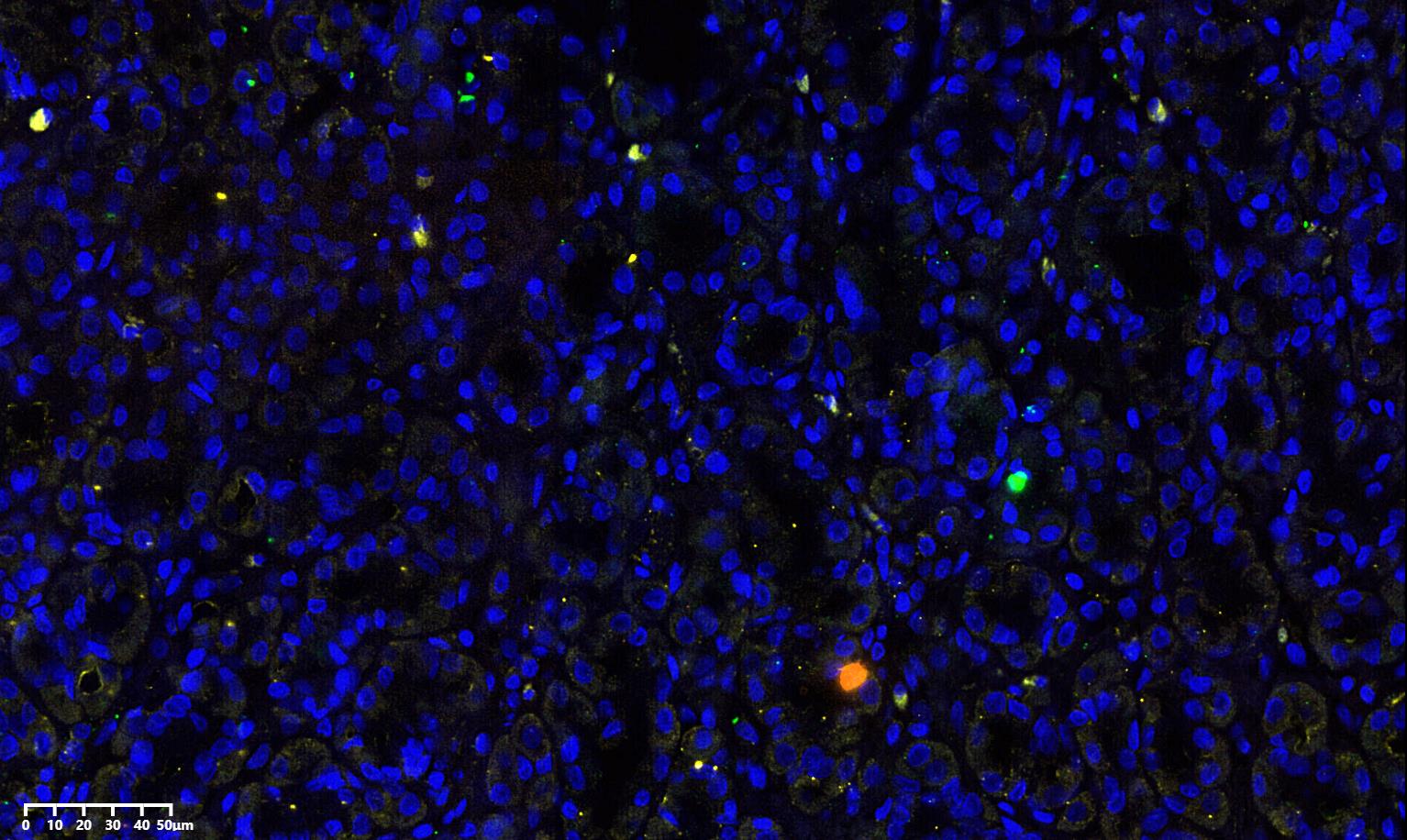

Supplement: Supplementary file 1 [file cancers-18-01384-s001.zip › original images/Microscopy/glioblastoma-3/LGG/MERGE.jpg]

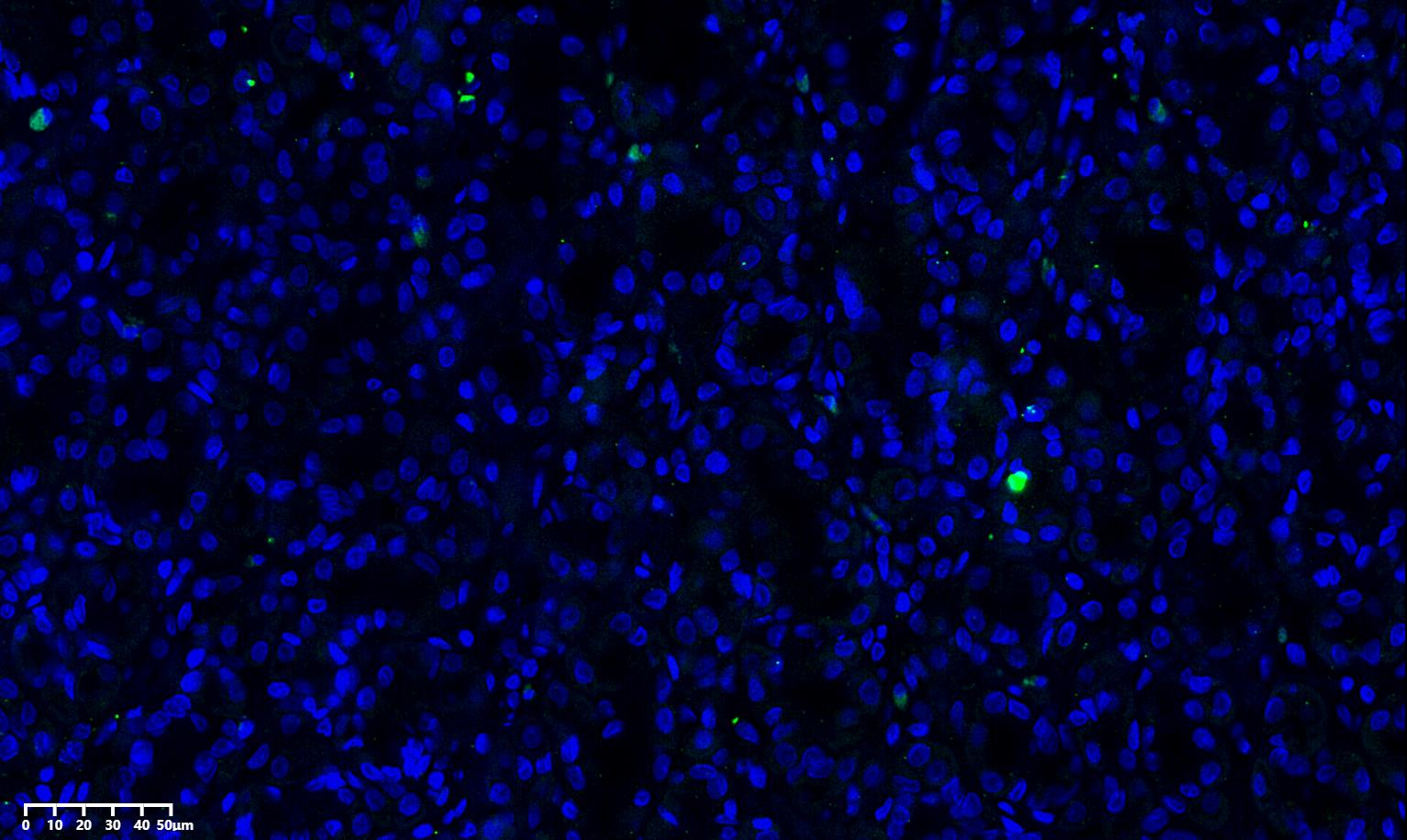

Supplement: Supplementary file 1 [file cancers-18-01384-s001.zip › original images/Microscopy/glioblastoma-3/LGG/METTL7B.jpg]

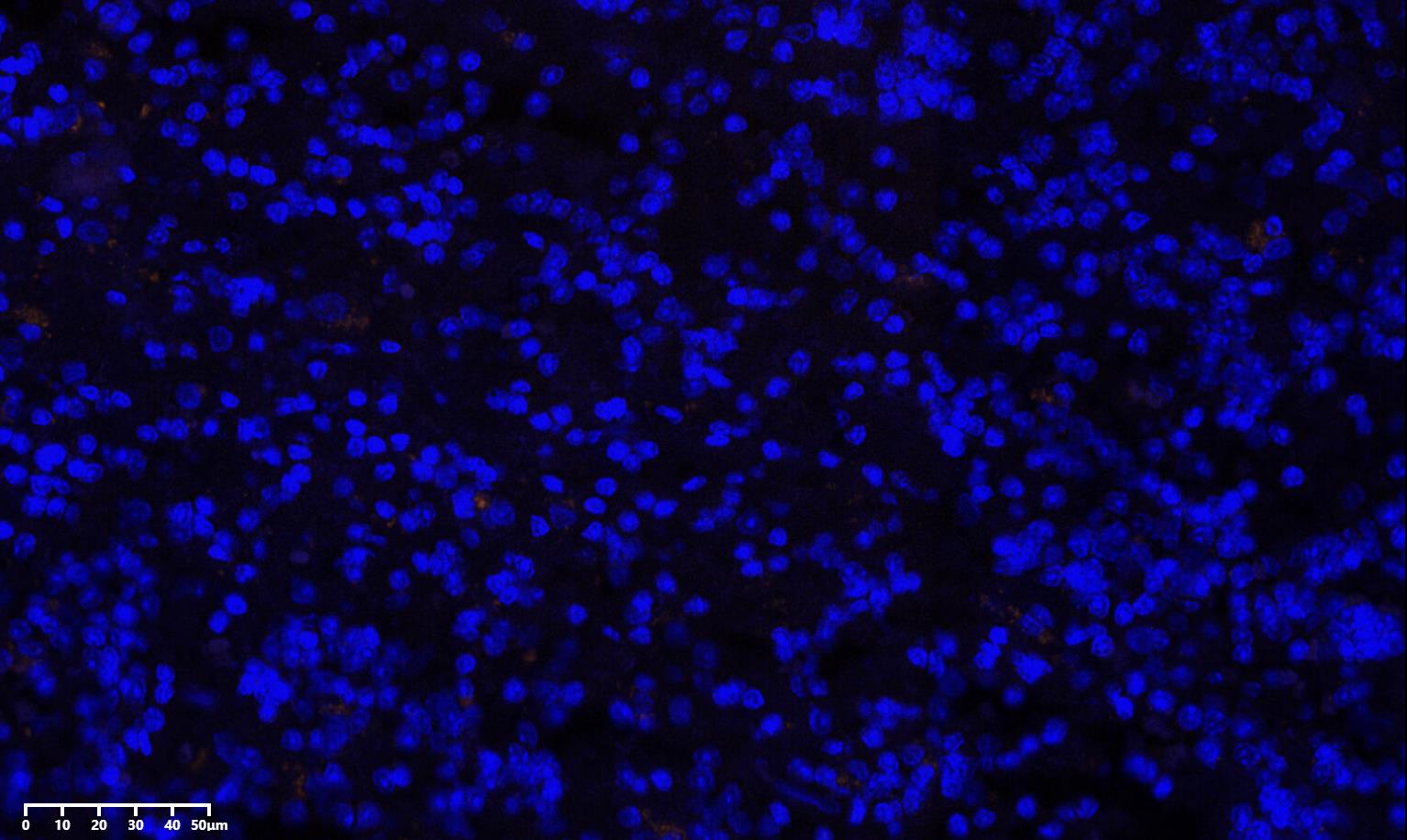

Supplement: Supplementary file 1 [file cancers-18-01384-s001.zip › original images/Microscopy/glioblastoma-3/Paracancerous/EPCAM.jpg]

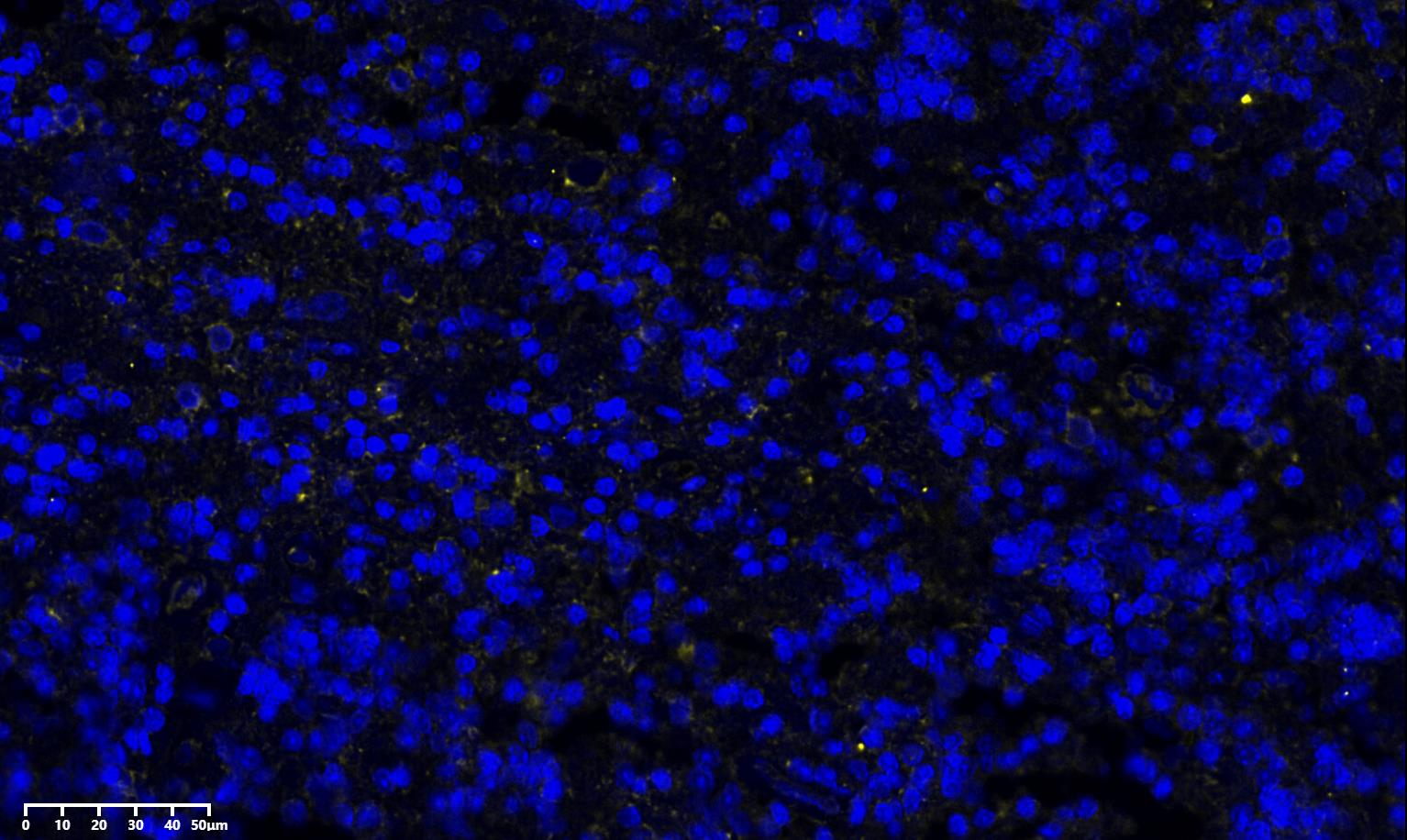

Supplement: Supplementary file 1 [file cancers-18-01384-s001.zip › original images/Microscopy/glioblastoma-3/Paracancerous/ITGA3.jpg]

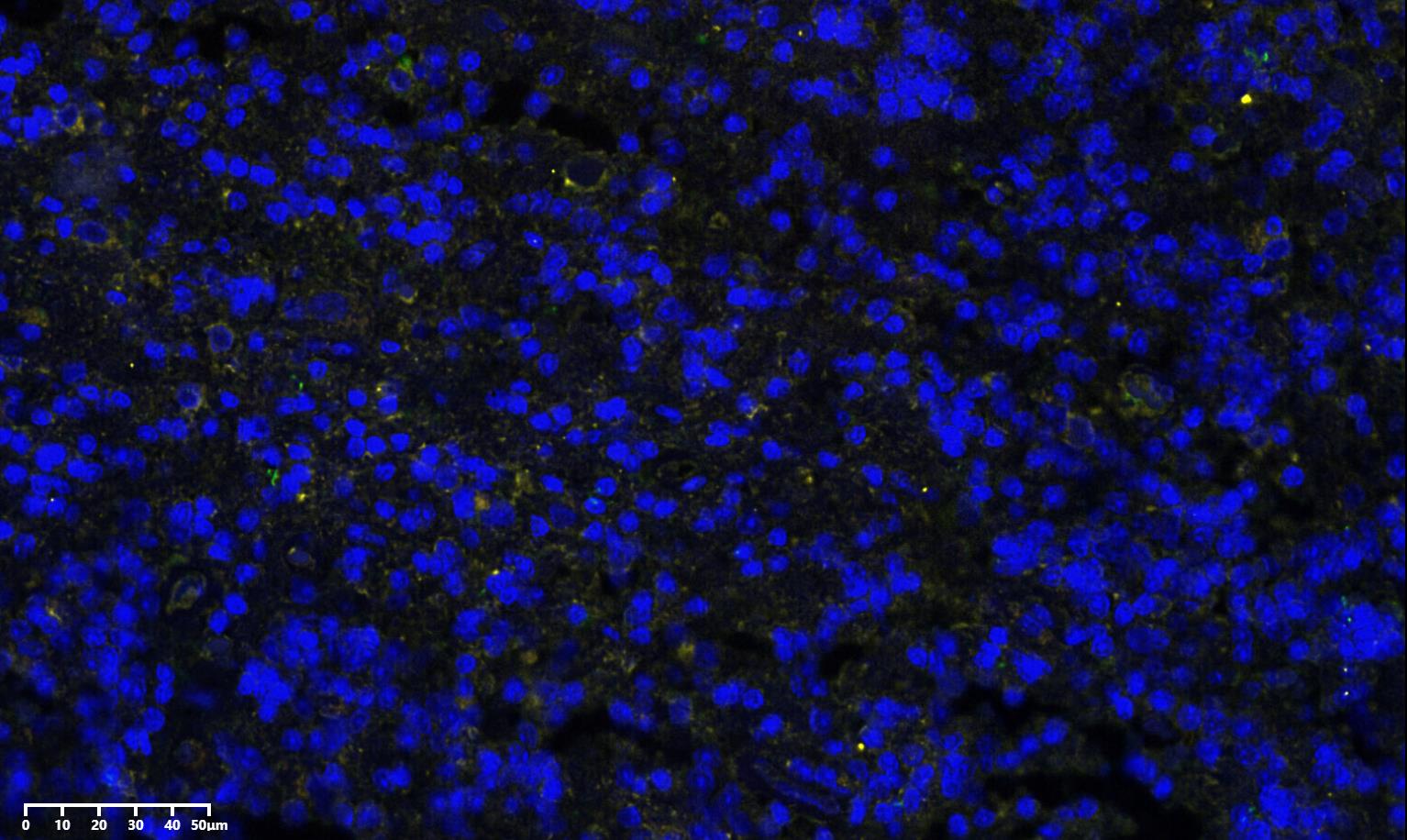

Supplement: Supplementary file 1 [file cancers-18-01384-s001.zip › original images/Microscopy/glioblastoma-3/Paracancerous/MERGE.jpg]

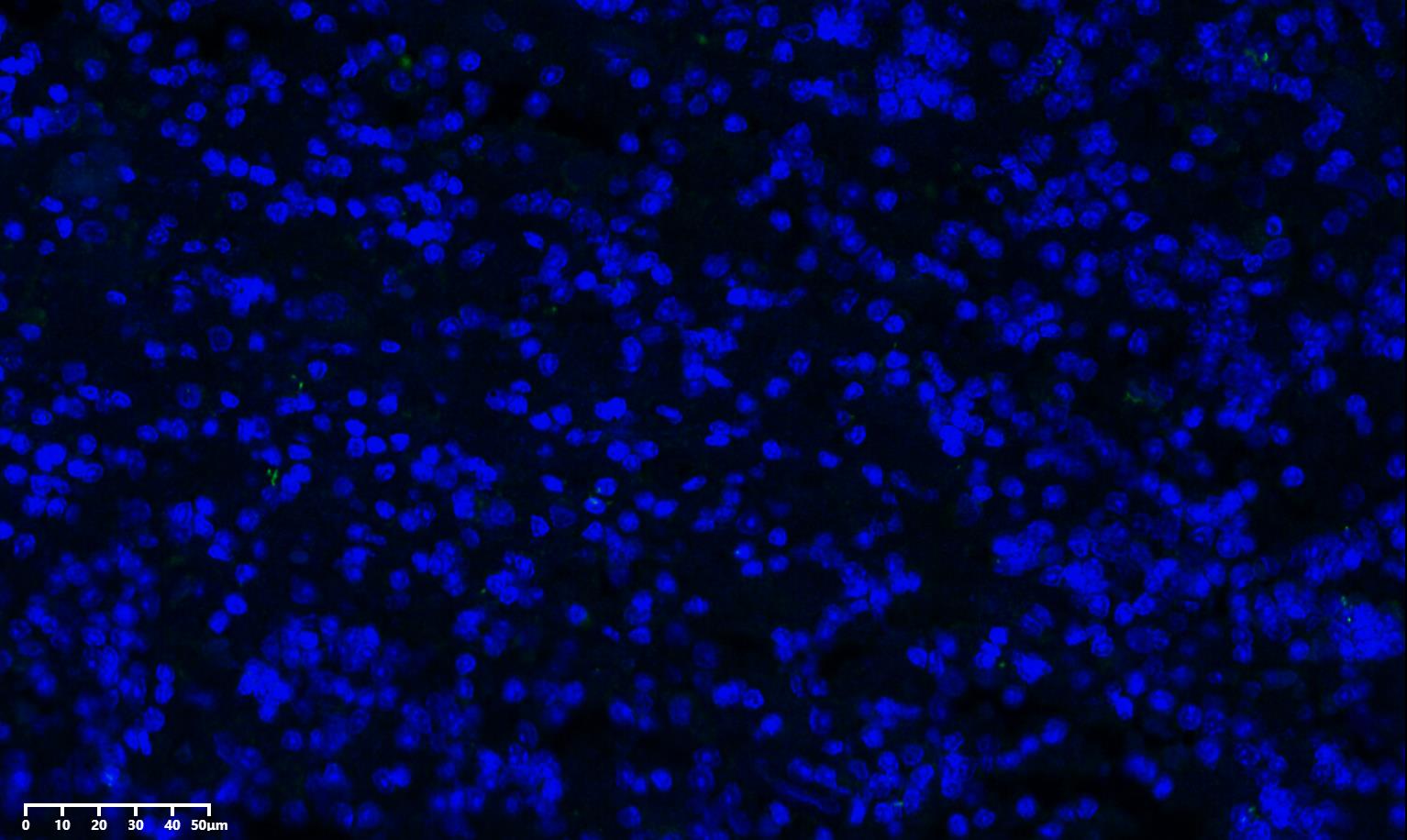

Supplement: Supplementary file 1 [file cancers-18-01384-s001.zip › original images/Microscopy/glioblastoma-3/Paracancerous/METTL7B.jpg]

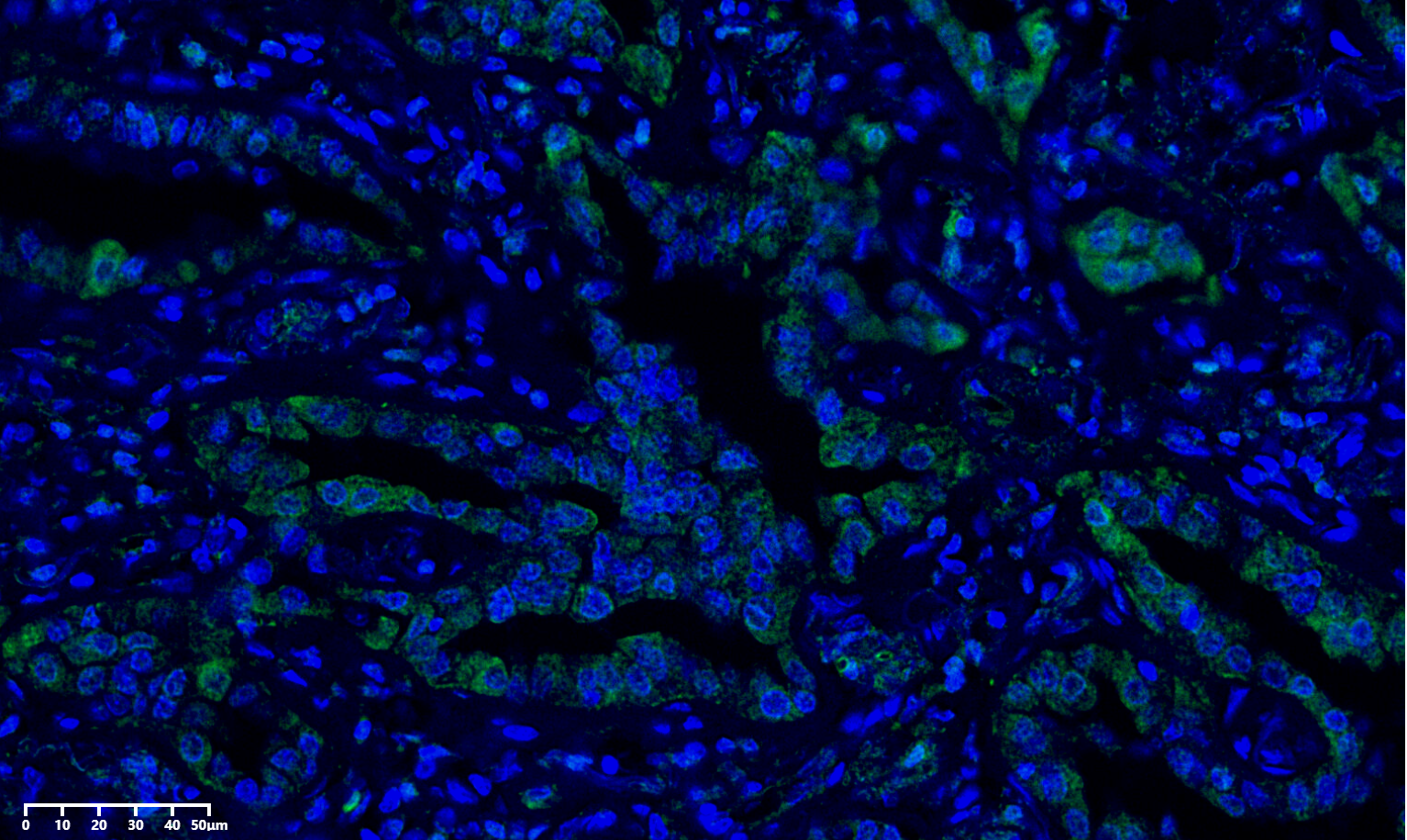

Supplement: Supplementary file 1 [file cancers-18-01384-s001.zip › original images/Microscopy/LUAD-1/IA/7B.tif]

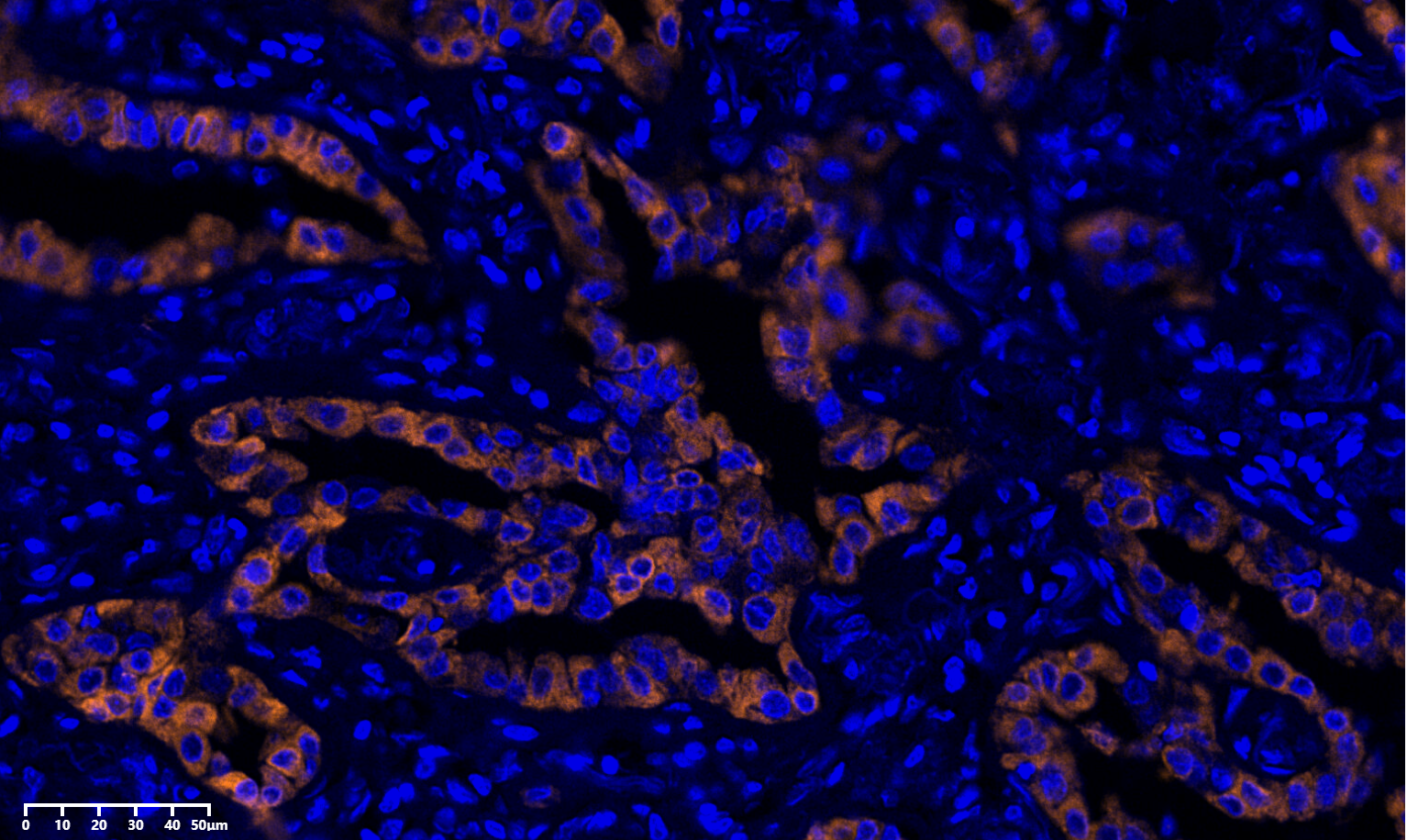

Supplement: Supplementary file 1 [file cancers-18-01384-s001.zip › original images/Microscopy/LUAD-1/IA/EPCAM.tif]

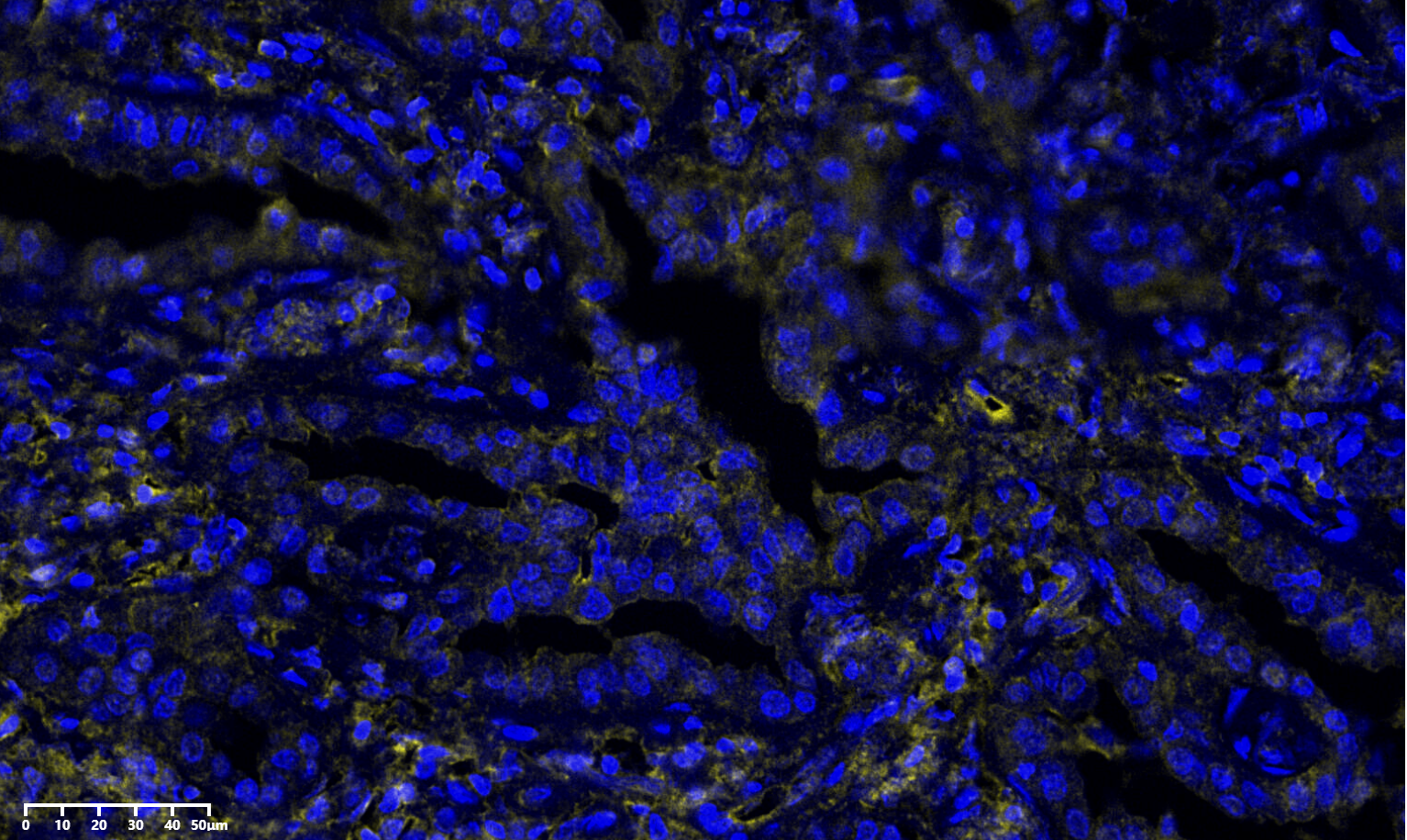

Supplement: Supplementary file 1 [file cancers-18-01384-s001.zip › original images/Microscopy/LUAD-1/IA/ITGA3.tif]

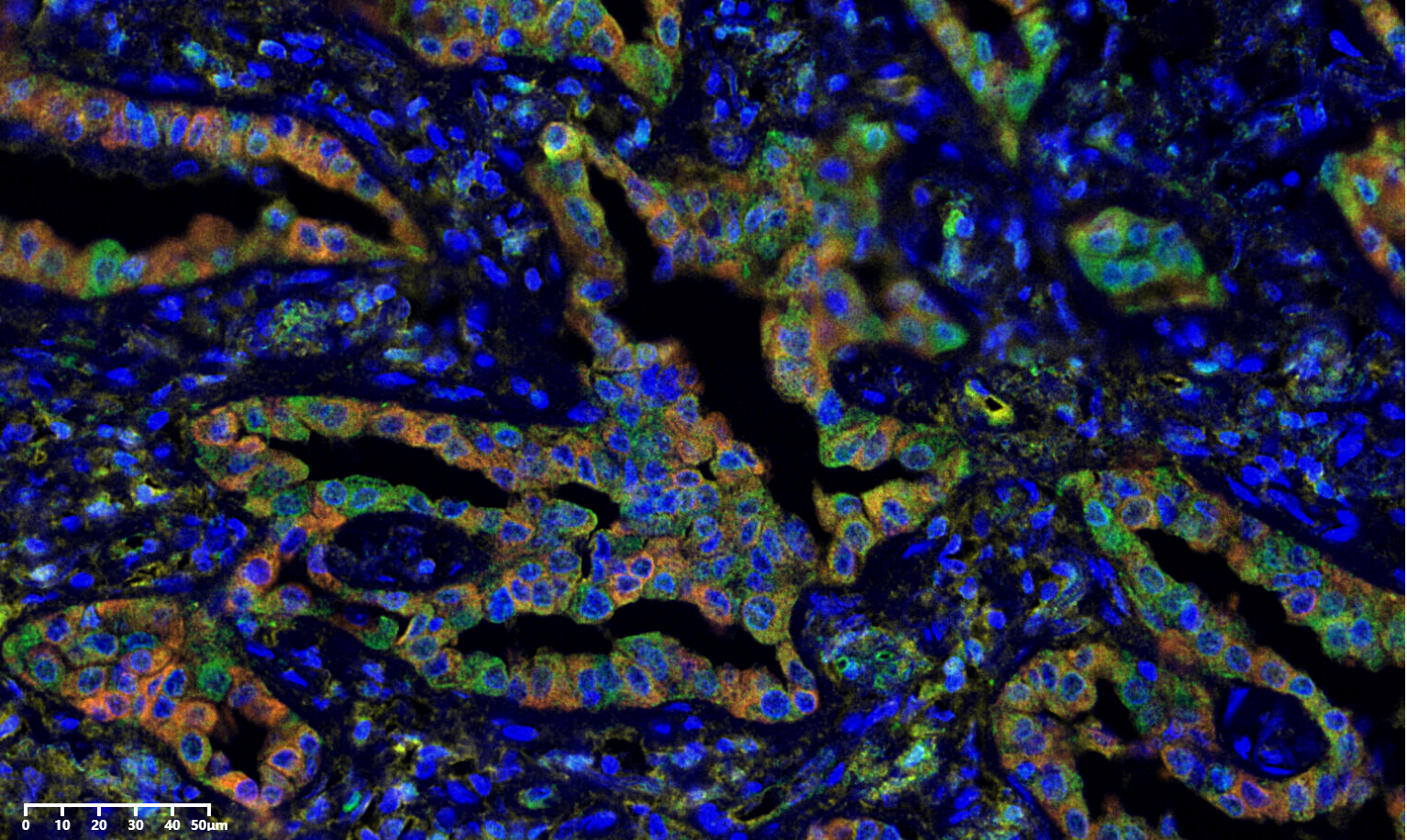

Supplement: Supplementary file 1 [file cancers-18-01384-s001.zip › original images/Microscopy/LUAD-1/IA/MERGE-01.tif]

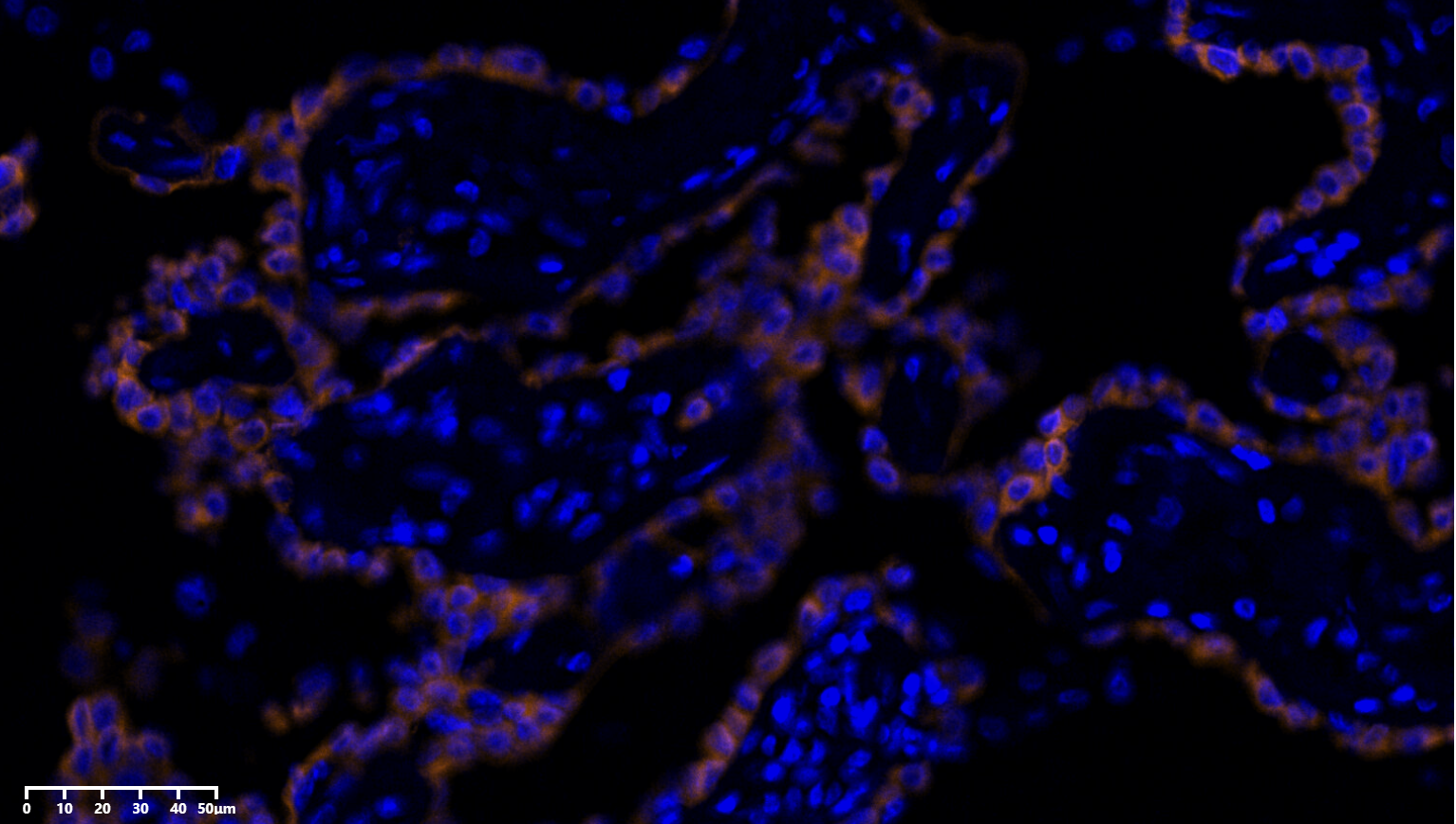

Supplement: Supplementary file 1 [file cancers-18-01384-s001.zip › original images/Microscopy/LUAD-1/MIA/EPCAM.tif]

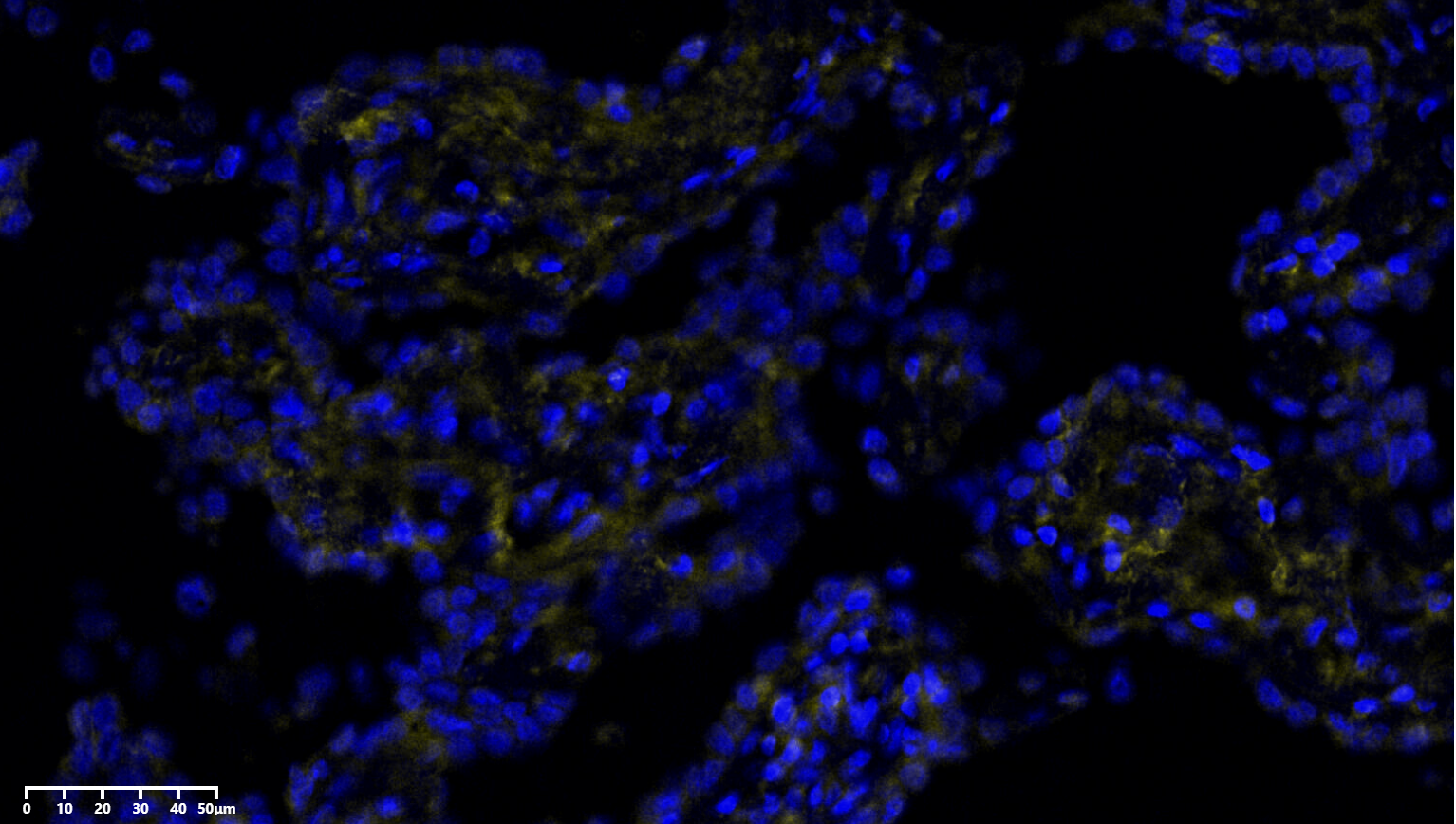

Supplement: Supplementary file 1 [file cancers-18-01384-s001.zip › original images/Microscopy/LUAD-1/MIA/ITGA3.tif]

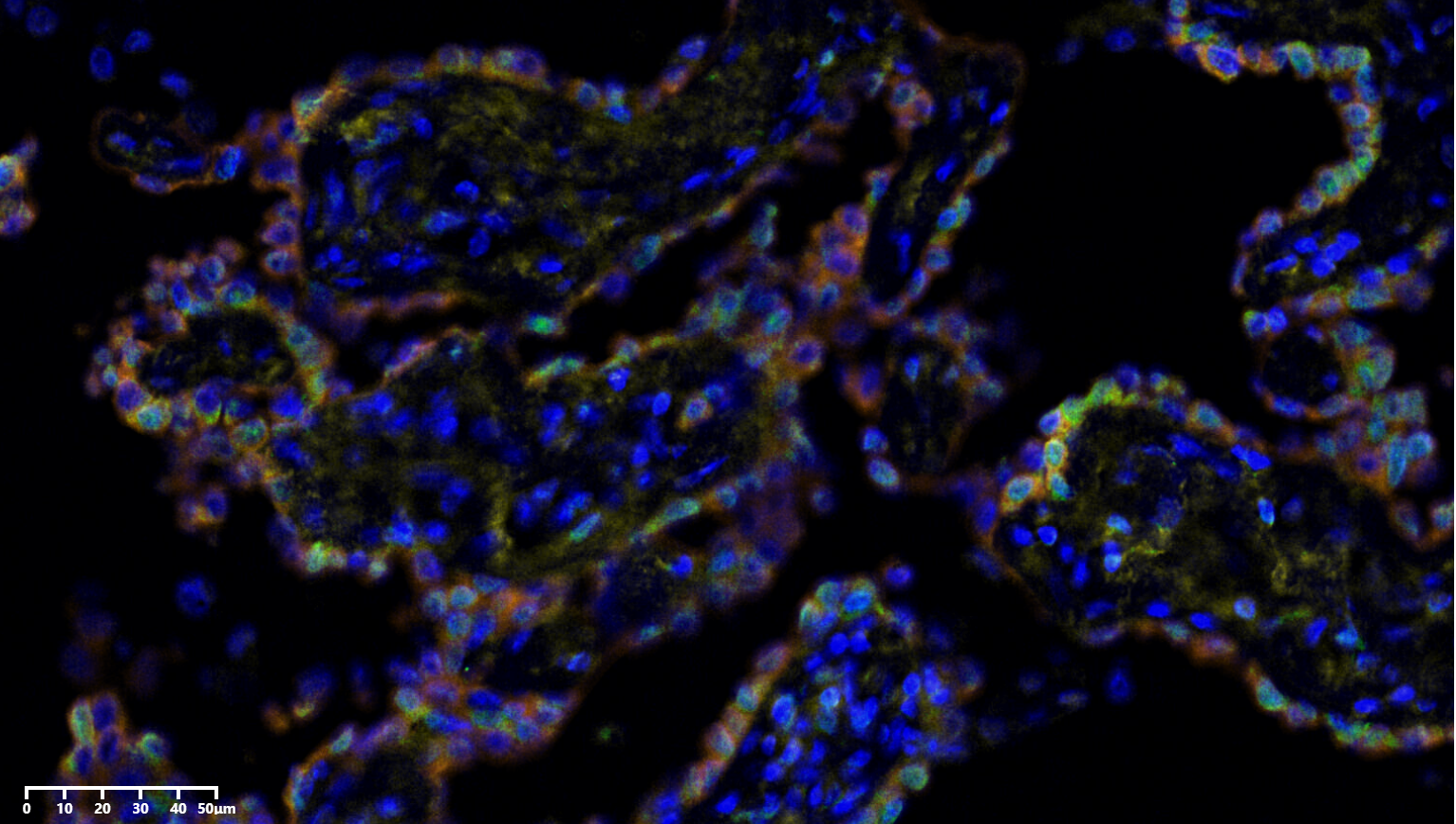

Supplement: Supplementary file 1 [file cancers-18-01384-s001.zip › original images/Microscopy/LUAD-1/MIA/MERGE.tif]

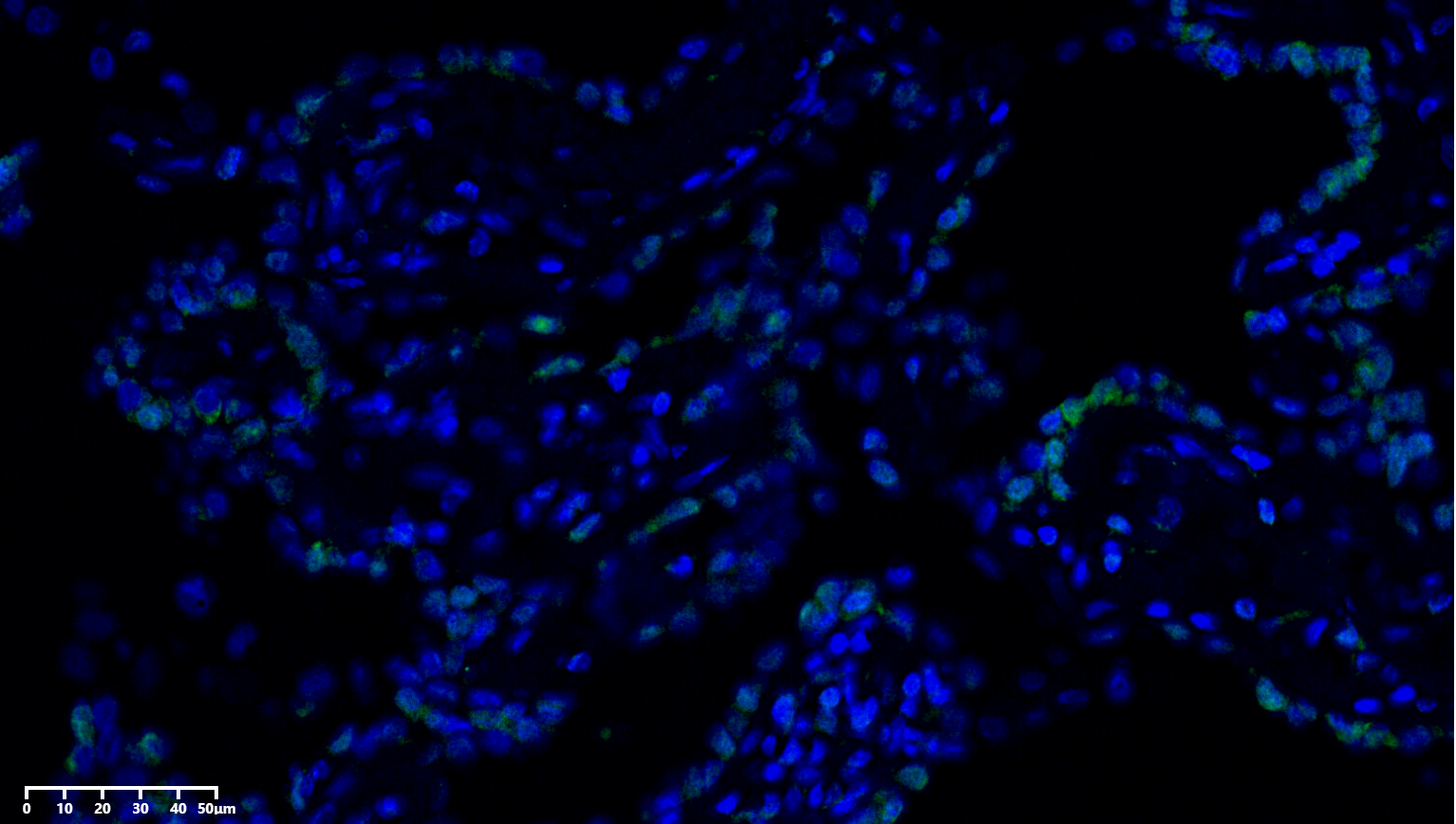

Supplement: Supplementary file 1 [file cancers-18-01384-s001.zip › original images/Microscopy/LUAD-1/MIA/METTL7B.tif]

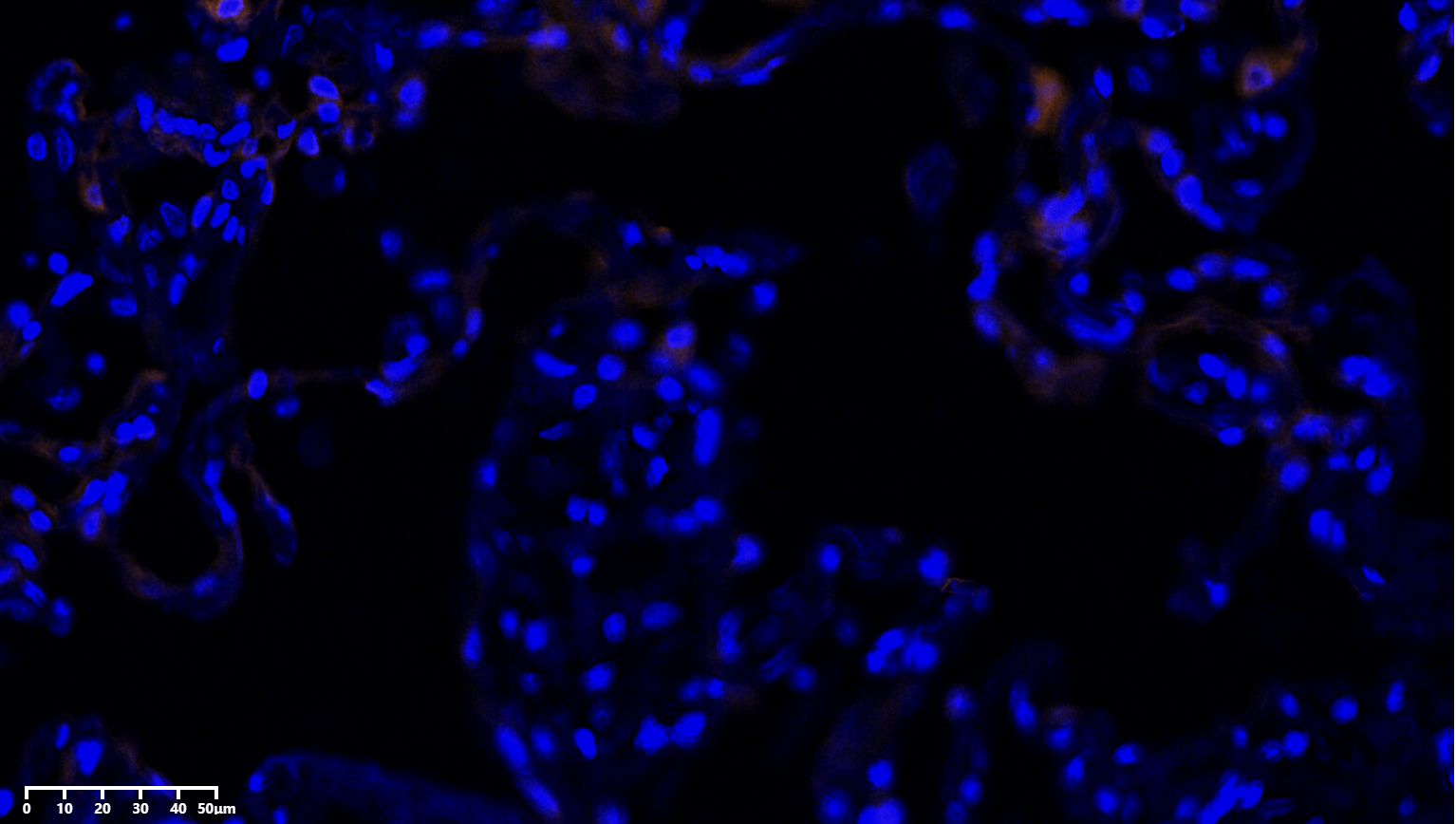

Supplement: Supplementary file 1 [file cancers-18-01384-s001.zip › original images/Microscopy/LUAD-1/Paracancerous/EPCAM.tif]

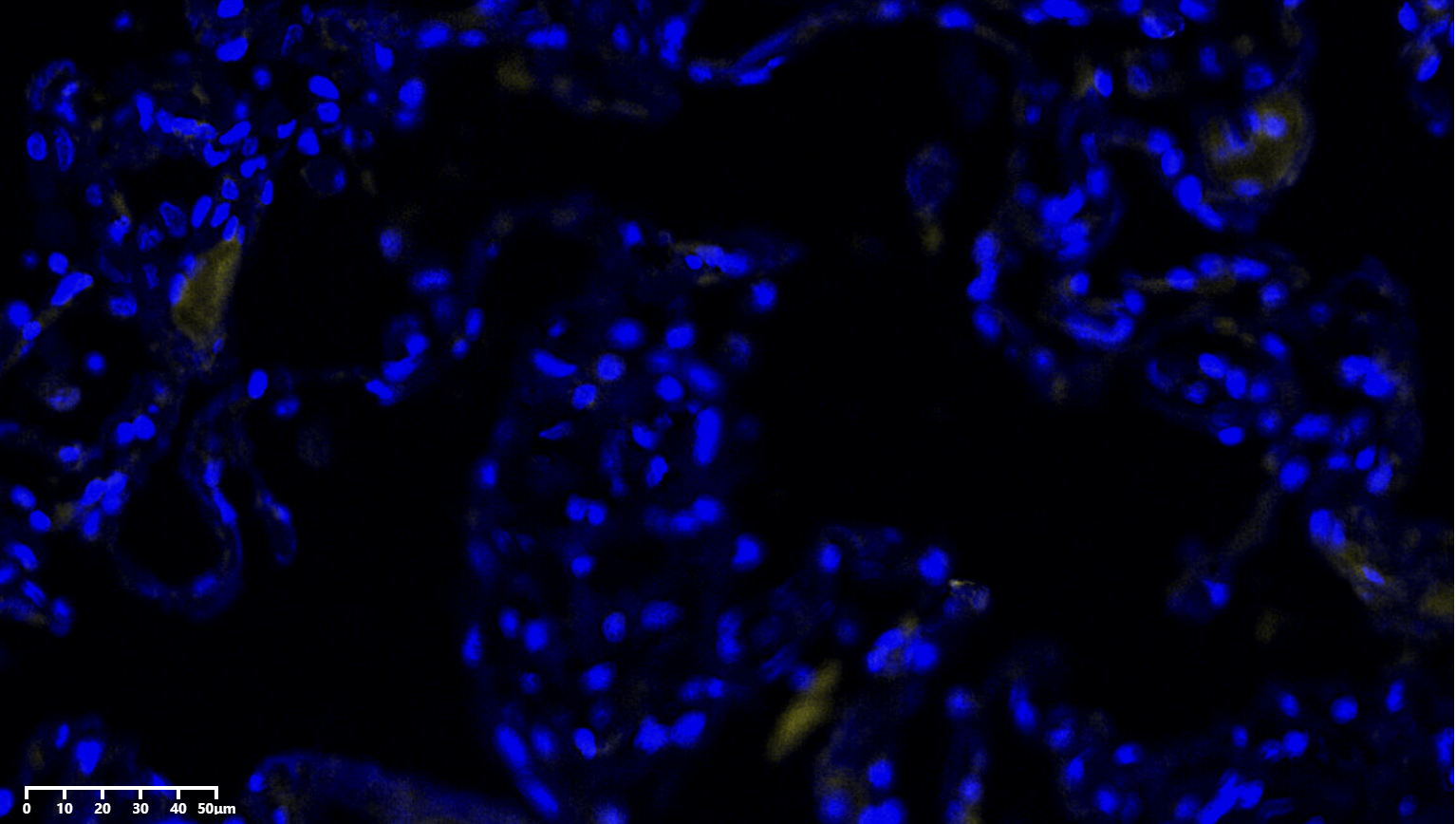

Supplement: Supplementary file 1 [file cancers-18-01384-s001.zip › original images/Microscopy/LUAD-1/Paracancerous/ITGA3.tif]

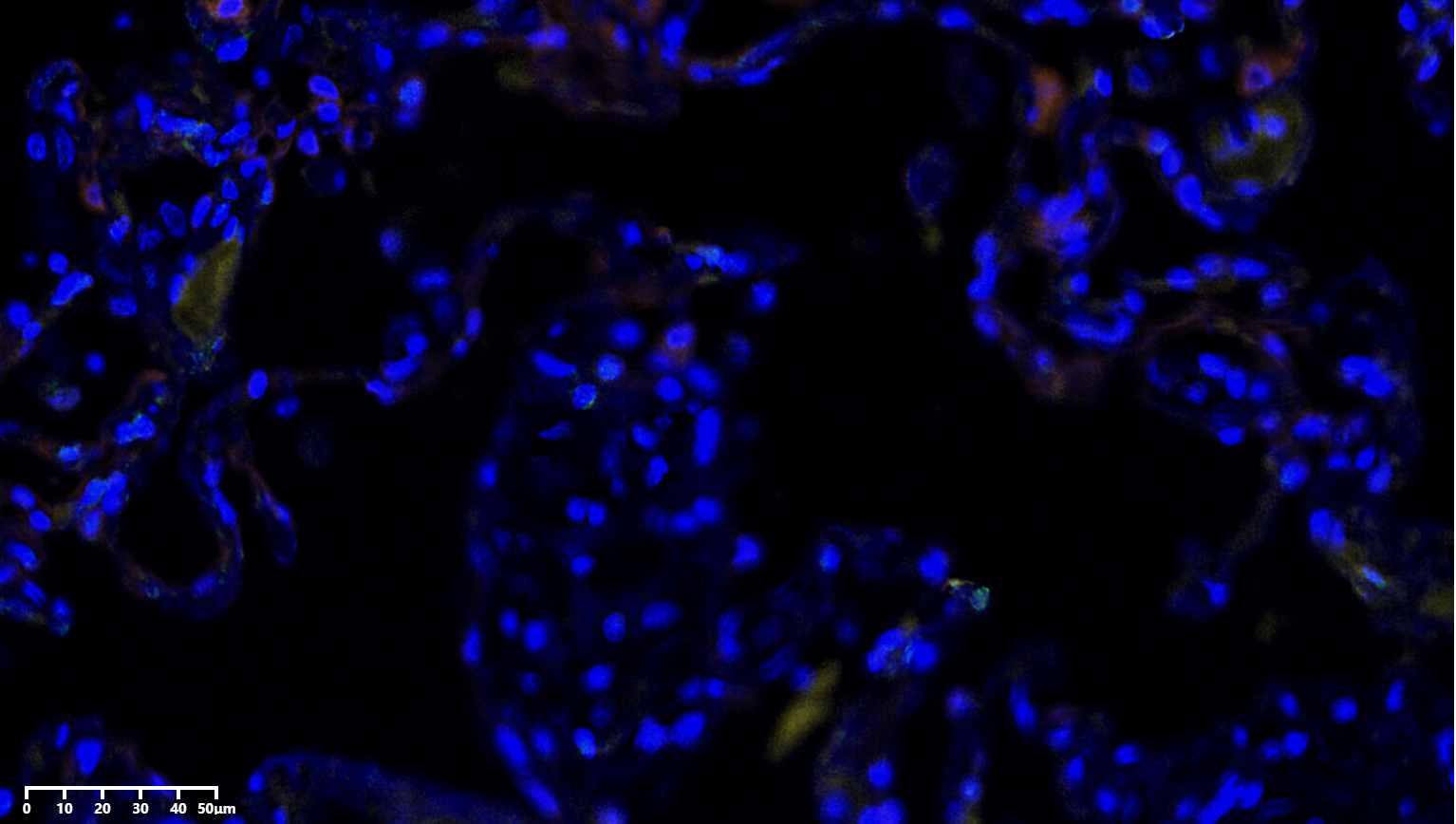

Supplement: Supplementary file 1 [file cancers-18-01384-s001.zip › original images/Microscopy/LUAD-1/Paracancerous/MERGE.tif]

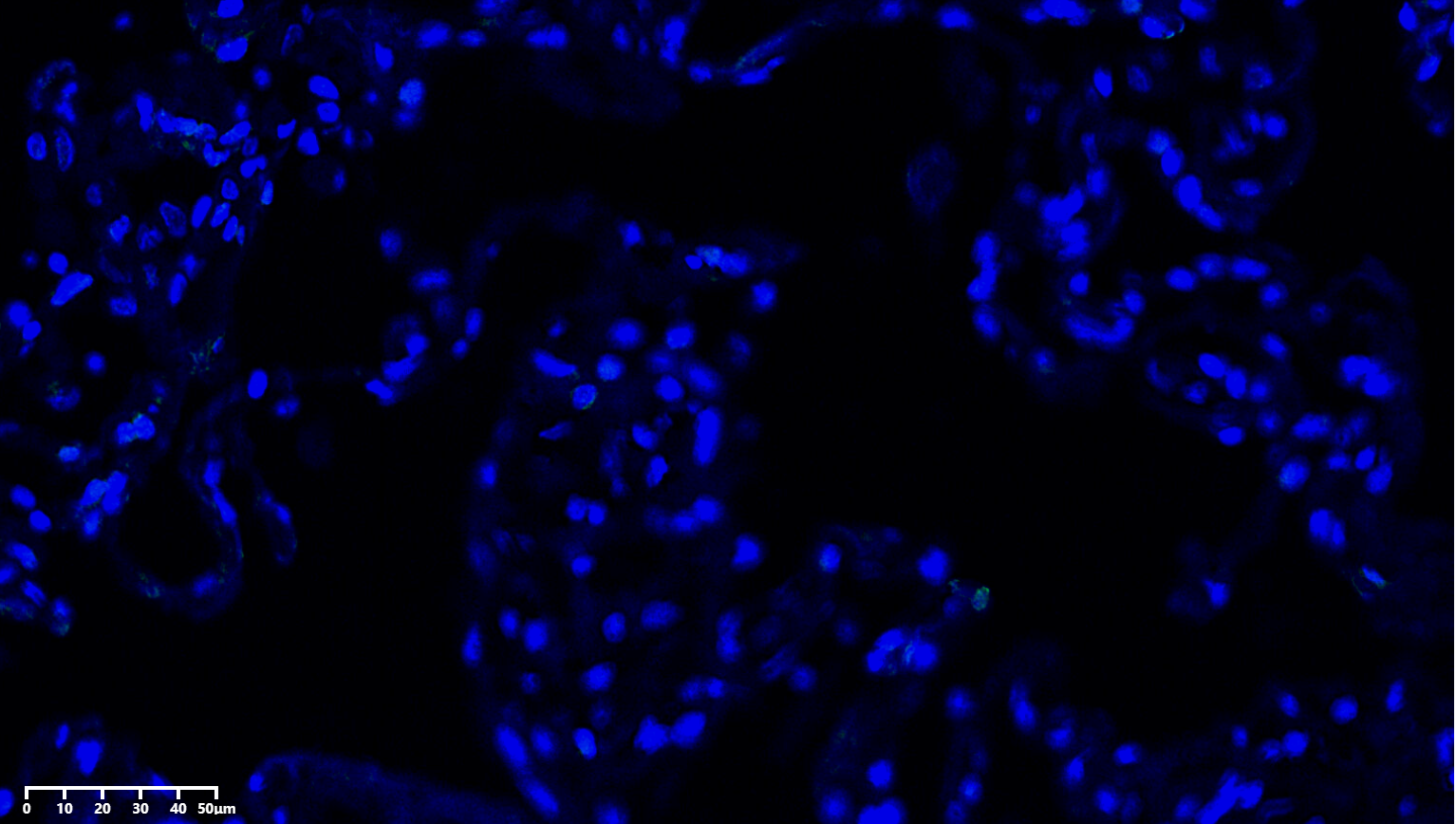

Supplement: Supplementary file 1 [file cancers-18-01384-s001.zip › original images/Microscopy/LUAD-1/Paracancerous/METTL7B.tif]

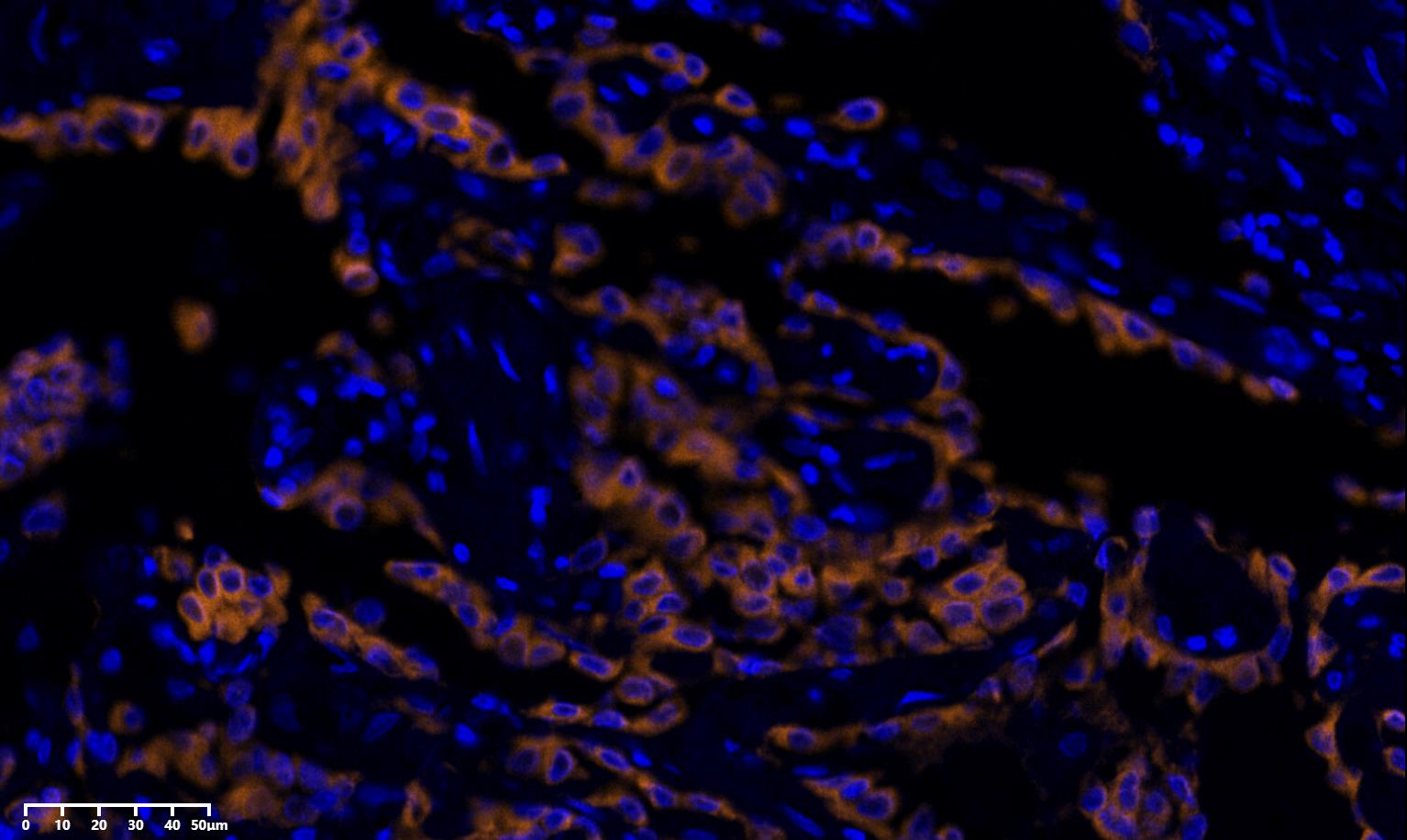

Supplement: Supplementary file 1 [file cancers-18-01384-s001.zip › original images/Microscopy/LUAD-2/IA/EPCAM.jpg]

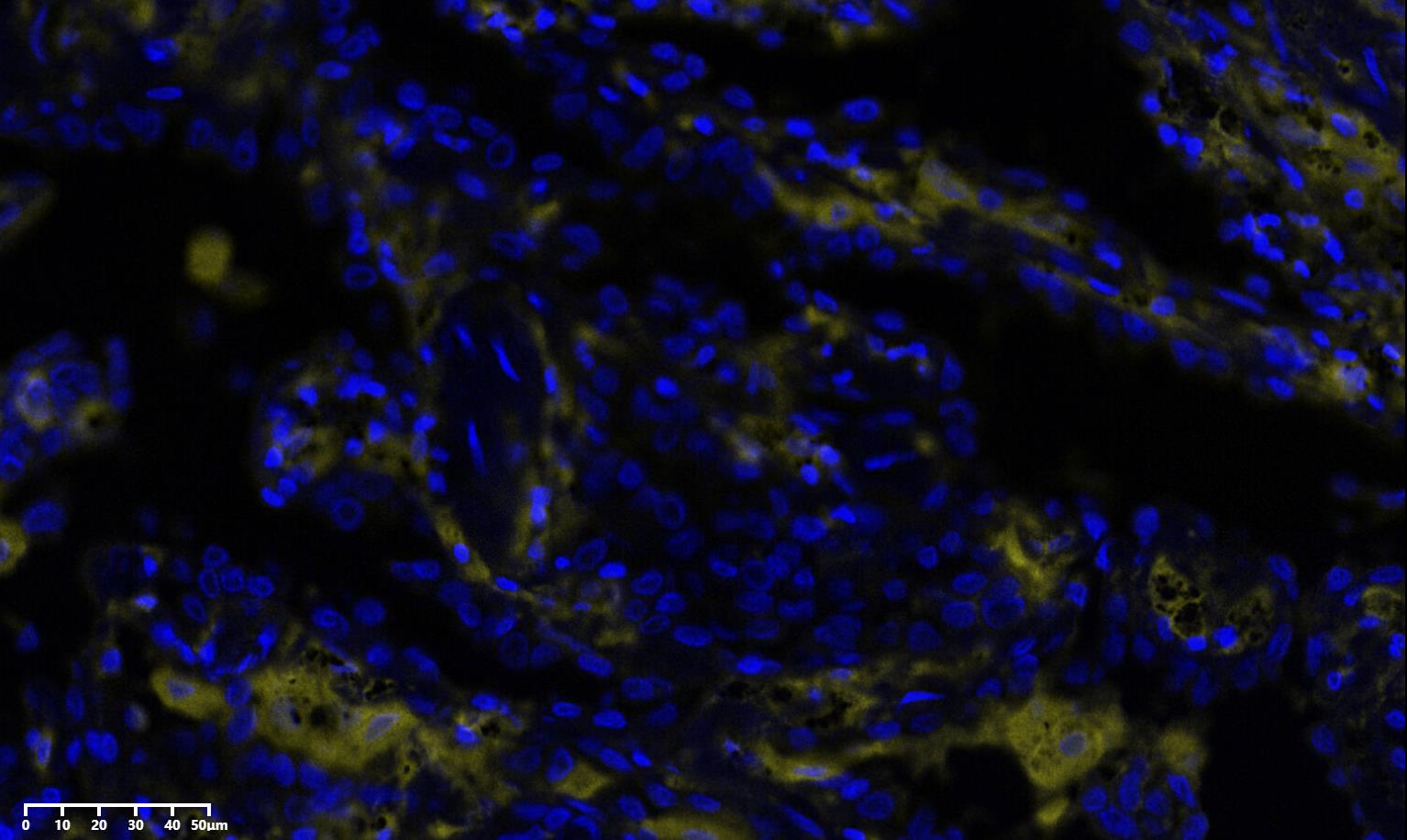

Supplement: Supplementary file 1 [file cancers-18-01384-s001.zip › original images/Microscopy/LUAD-2/IA/ITGA3.jpg]

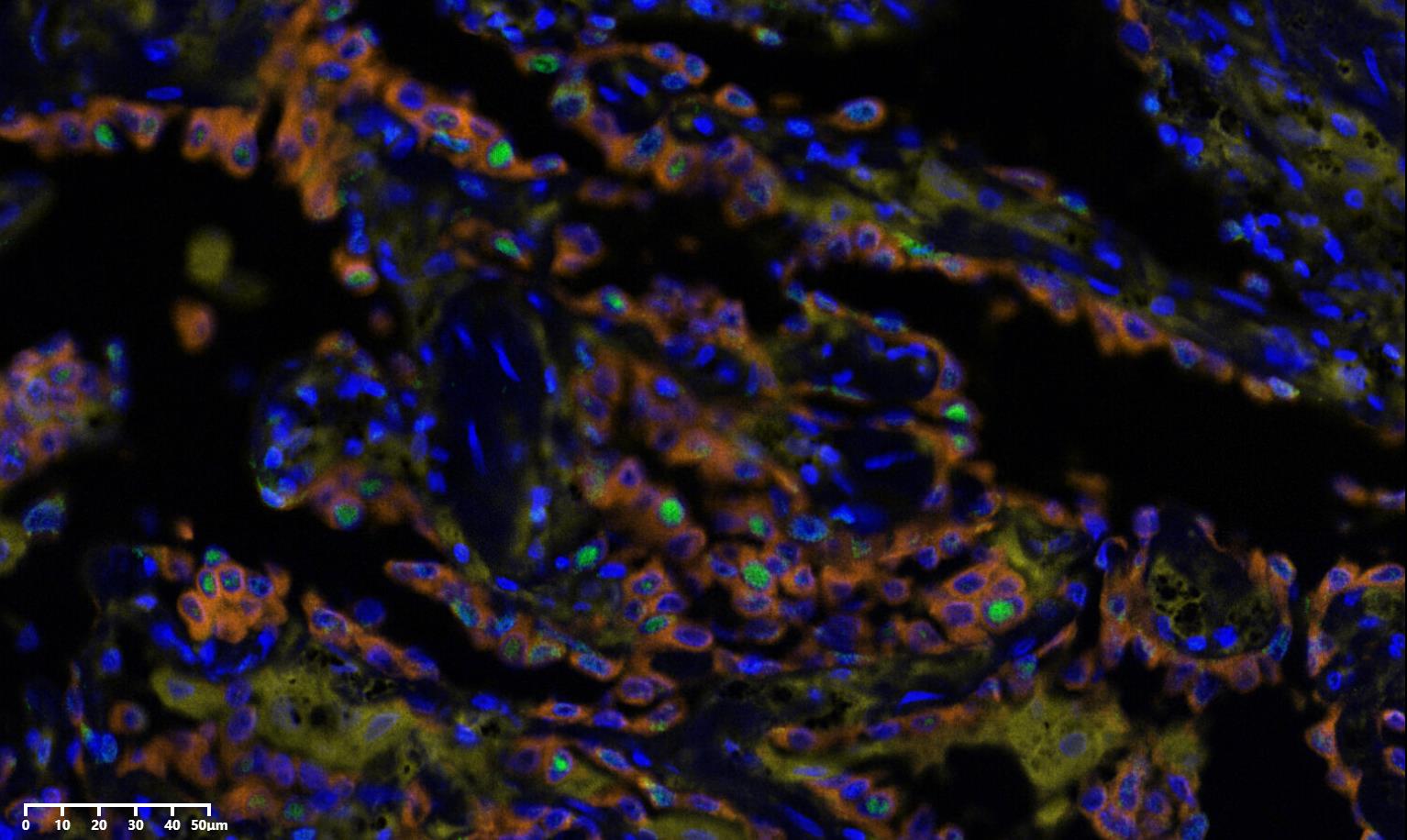

Supplement: Supplementary file 1 [file cancers-18-01384-s001.zip › original images/Microscopy/LUAD-2/IA/MERGE.jpg]

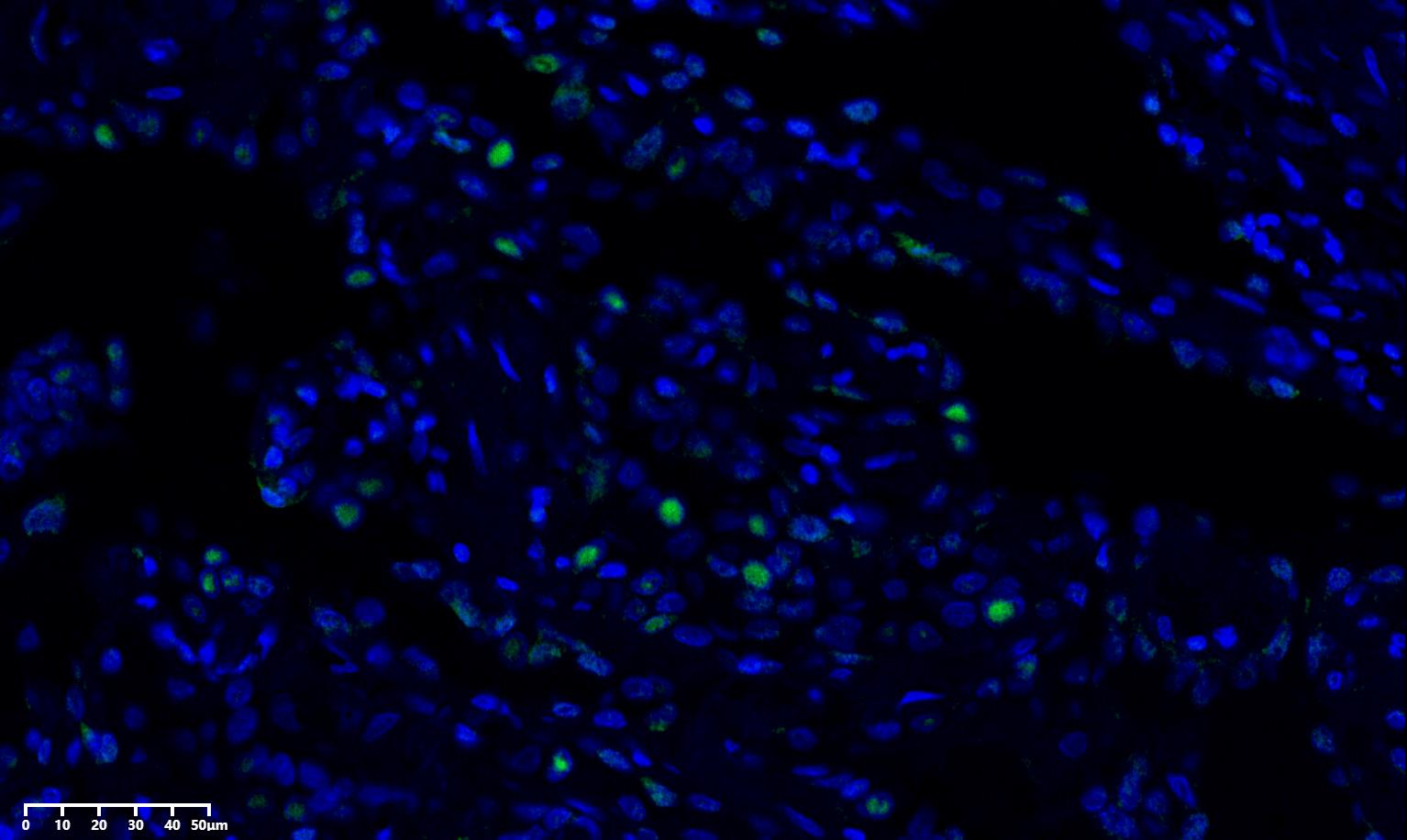

Supplement: Supplementary file 1 [file cancers-18-01384-s001.zip › original images/Microscopy/LUAD-2/IA/METTL7B.jpg]

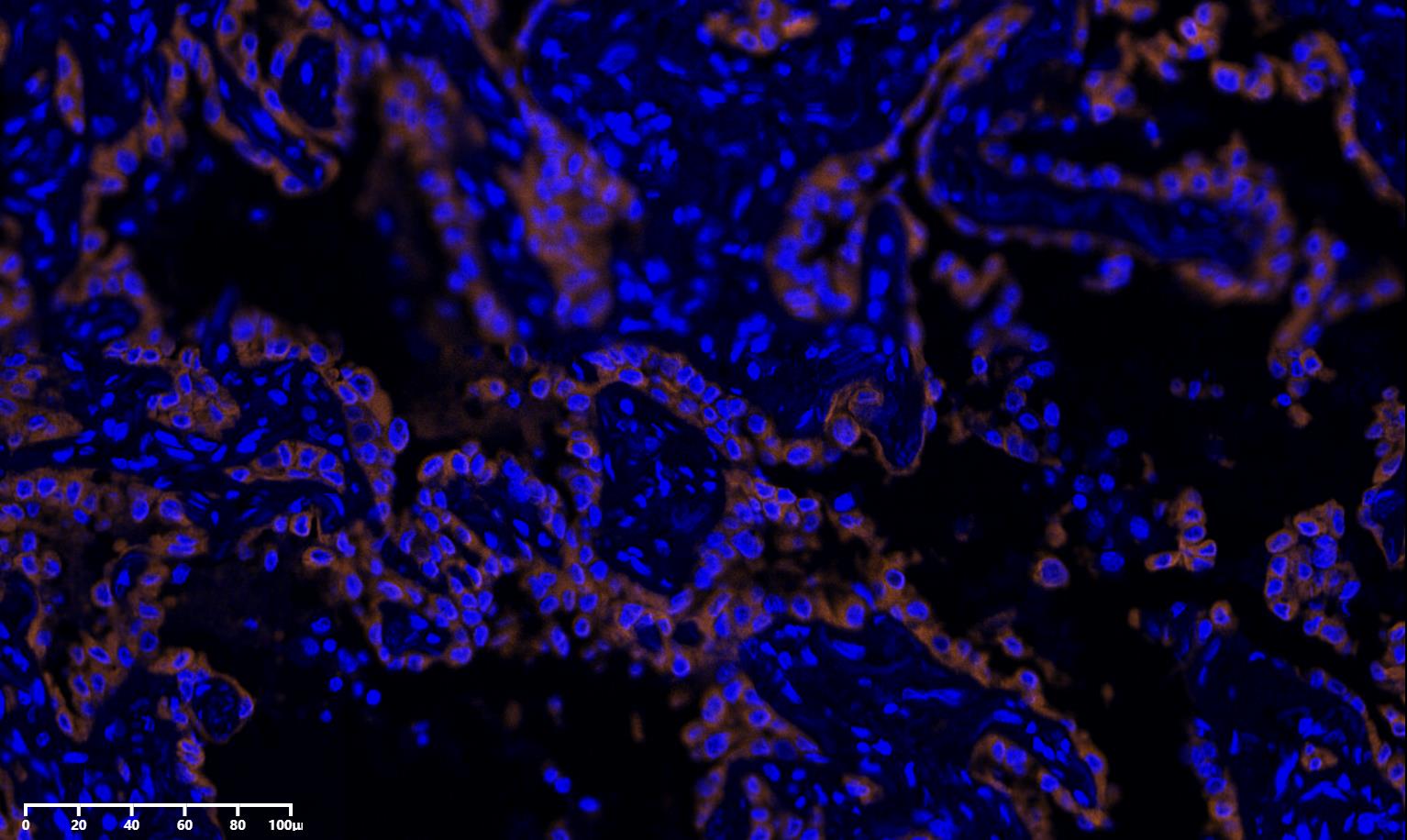

Supplement: Supplementary file 1 [file cancers-18-01384-s001.zip › original images/Microscopy/LUAD-2/MIA/EPCAM.jpg]

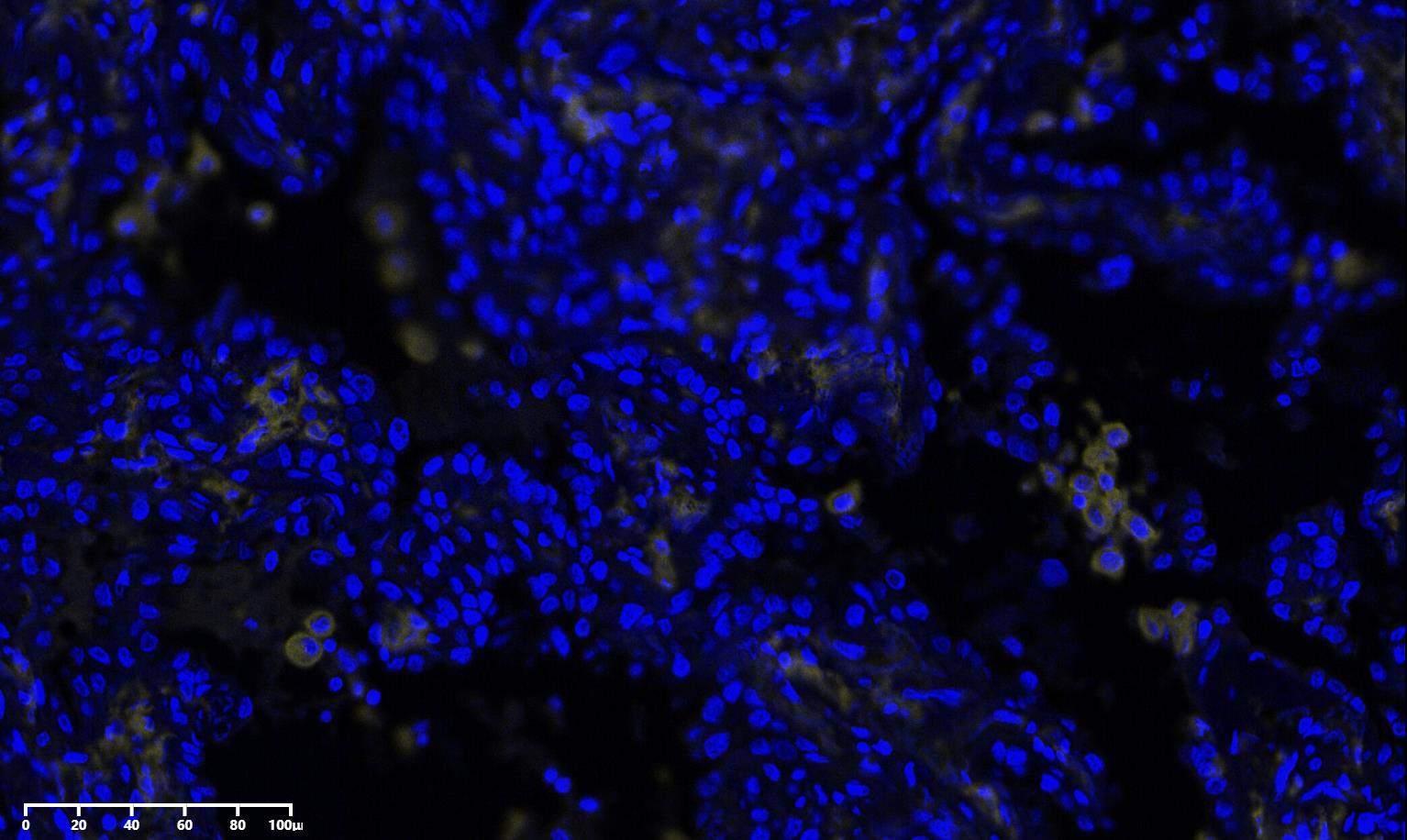

Supplement: Supplementary file 1 [file cancers-18-01384-s001.zip › original images/Microscopy/LUAD-2/MIA/ITGA3.jpg]

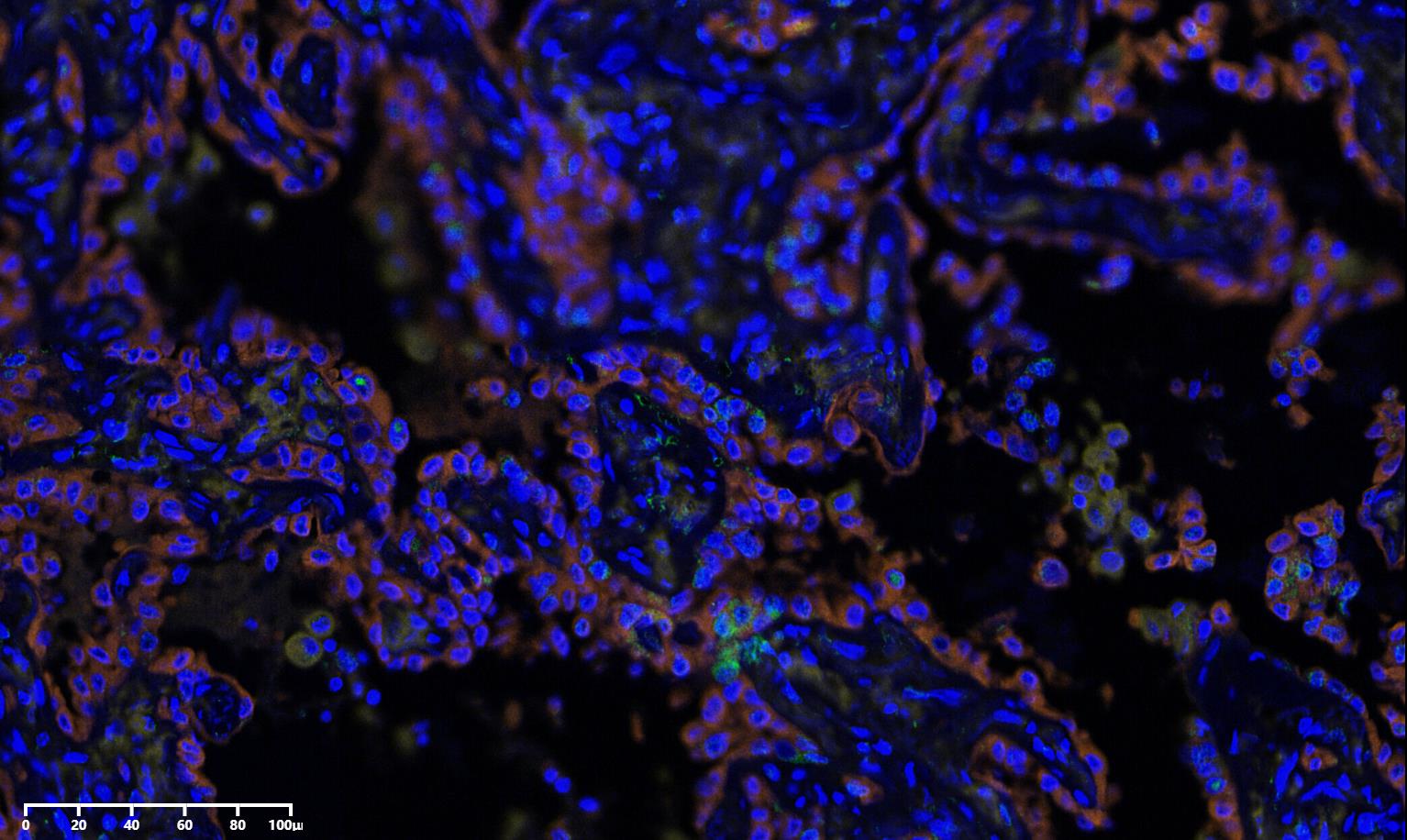

Supplement: Supplementary file 1 [file cancers-18-01384-s001.zip › original images/Microscopy/LUAD-2/MIA/MERGE.jpg]

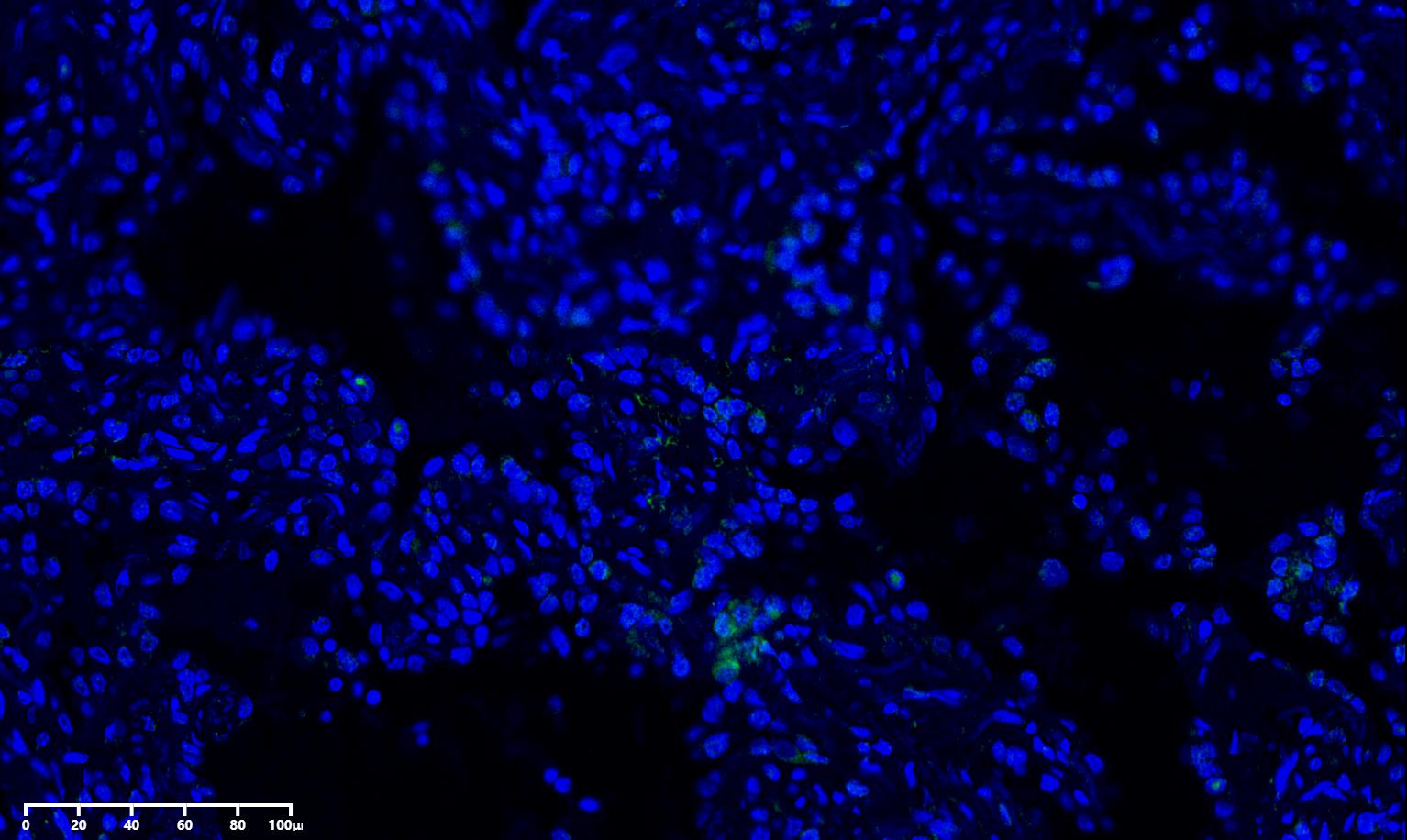

Supplement: Supplementary file 1 [file cancers-18-01384-s001.zip › original images/Microscopy/LUAD-2/MIA/METTL7B.jpg]

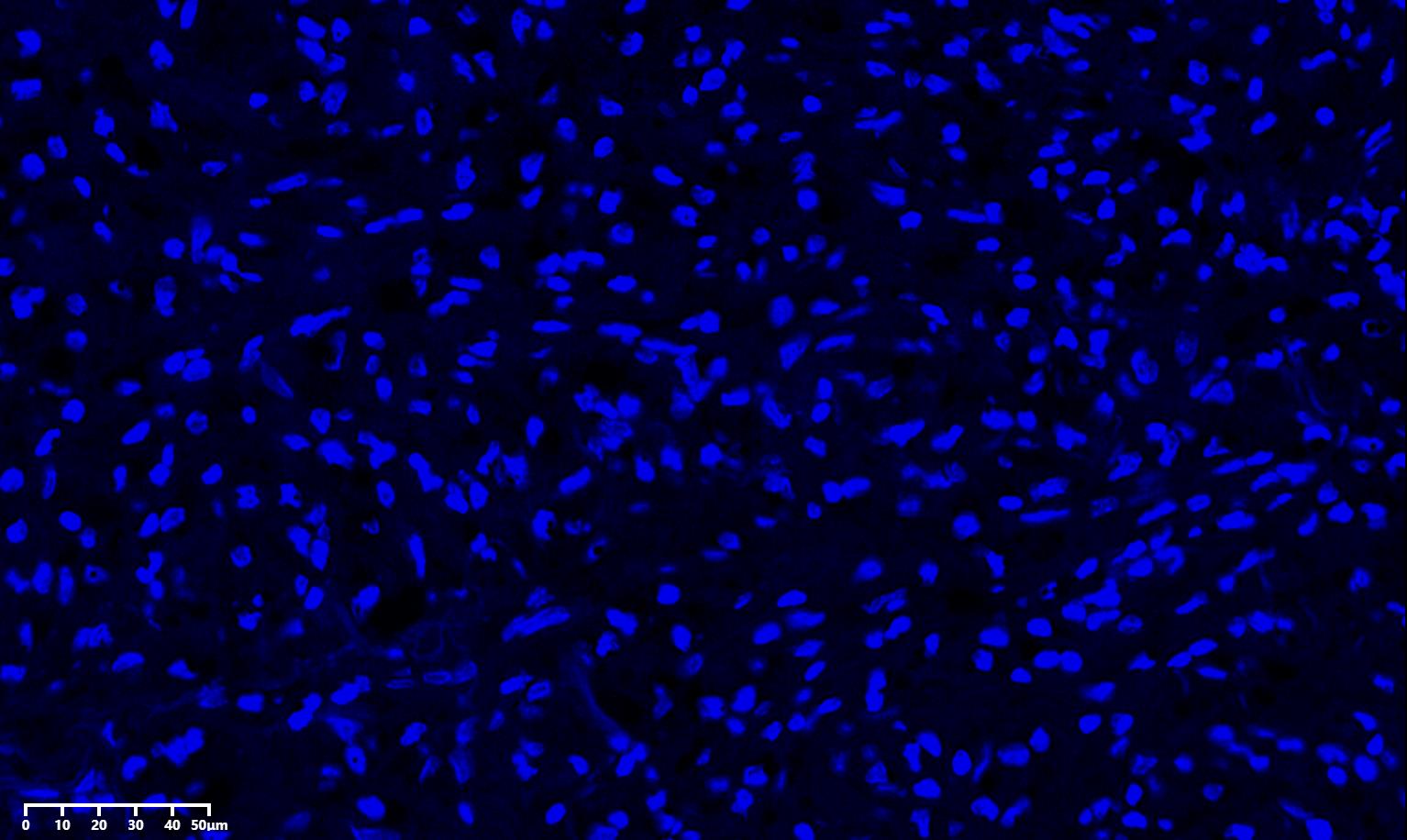

Supplement: Supplementary file 1 [file cancers-18-01384-s001.zip › original images/Microscopy/LUAD-2/paracanrous/EPCAM.jpg]

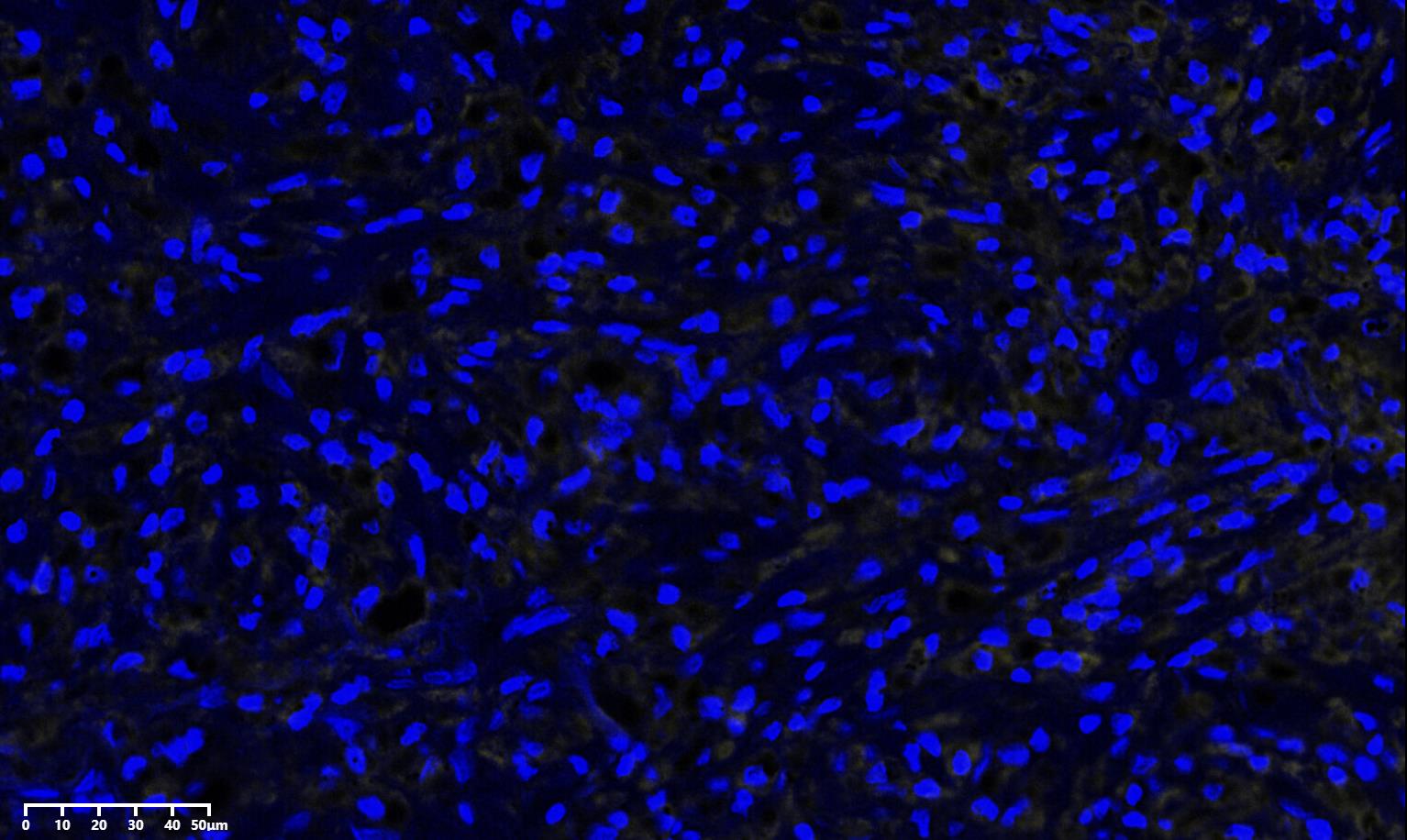

Supplement: Supplementary file 1 [file cancers-18-01384-s001.zip › original images/Microscopy/LUAD-2/paracanrous/ITGA3.jpg]

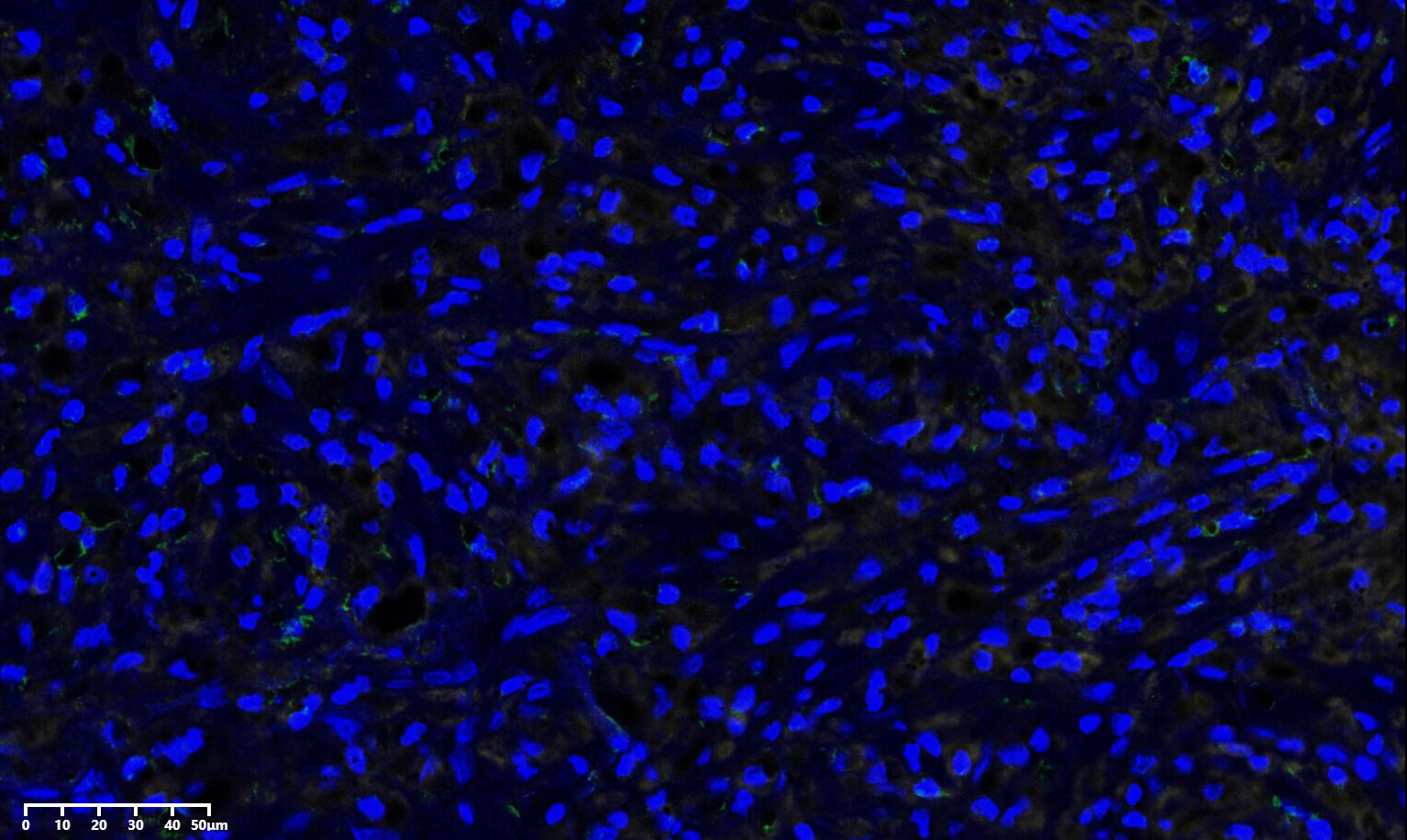

Supplement: Supplementary file 1 [file cancers-18-01384-s001.zip › original images/Microscopy/LUAD-2/paracanrous/MERGE.jpg]

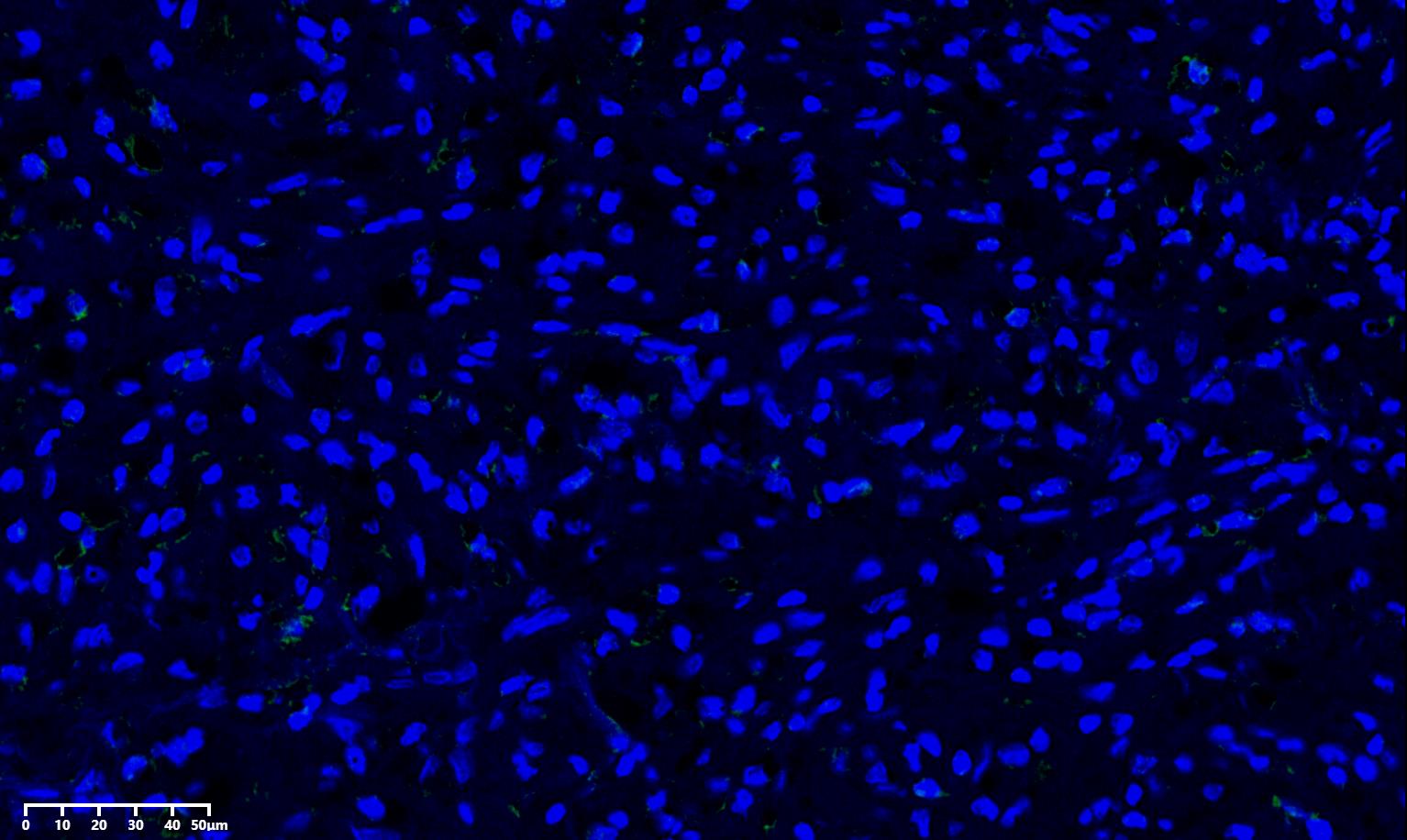

Supplement: Supplementary file 1 [file cancers-18-01384-s001.zip › original images/Microscopy/LUAD-2/paracanrous/METTL7B.jpg]

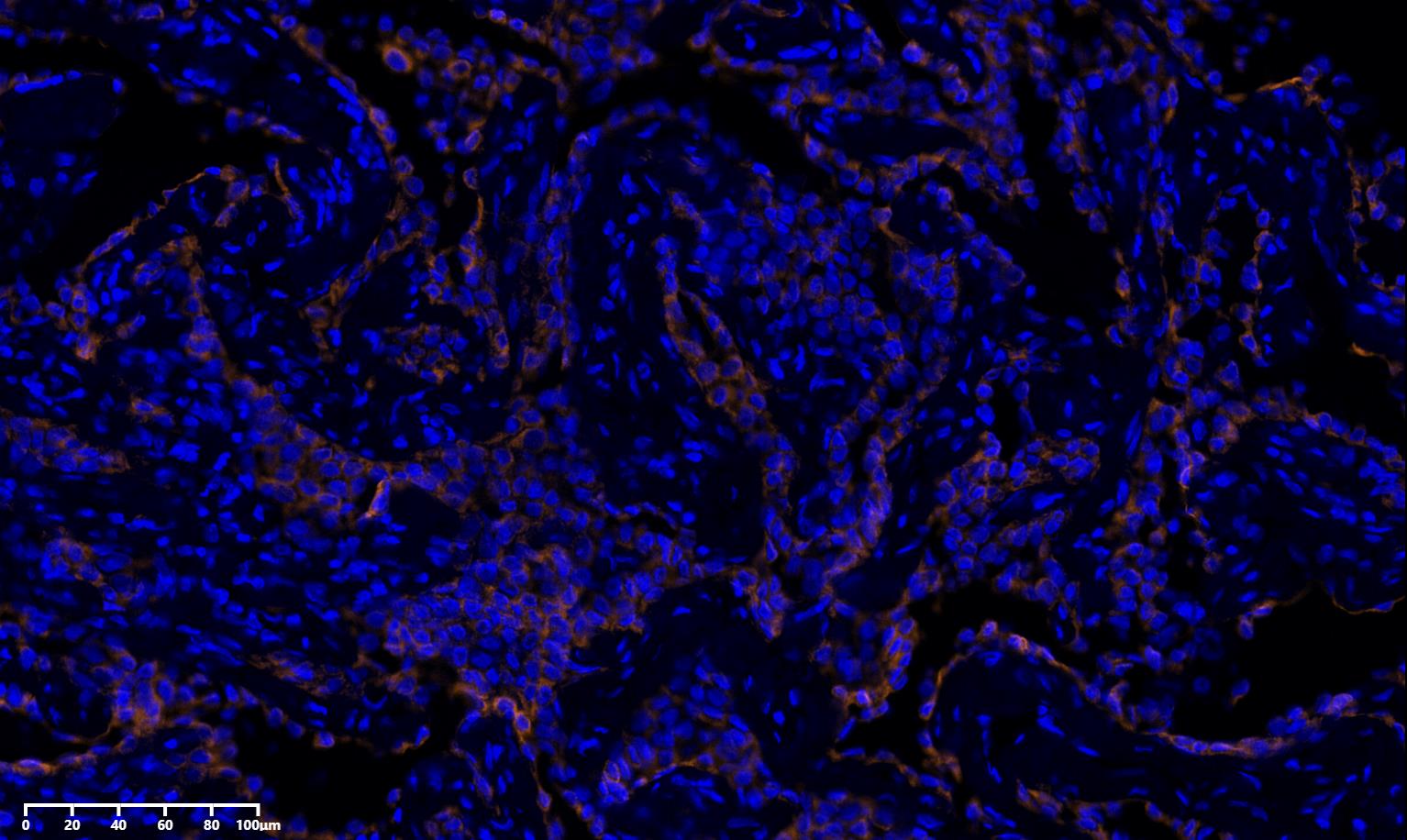

Supplement: Supplementary file 1 [file cancers-18-01384-s001.zip › original images/Microscopy/LUAD-3/IA/EPCAM.jpg]

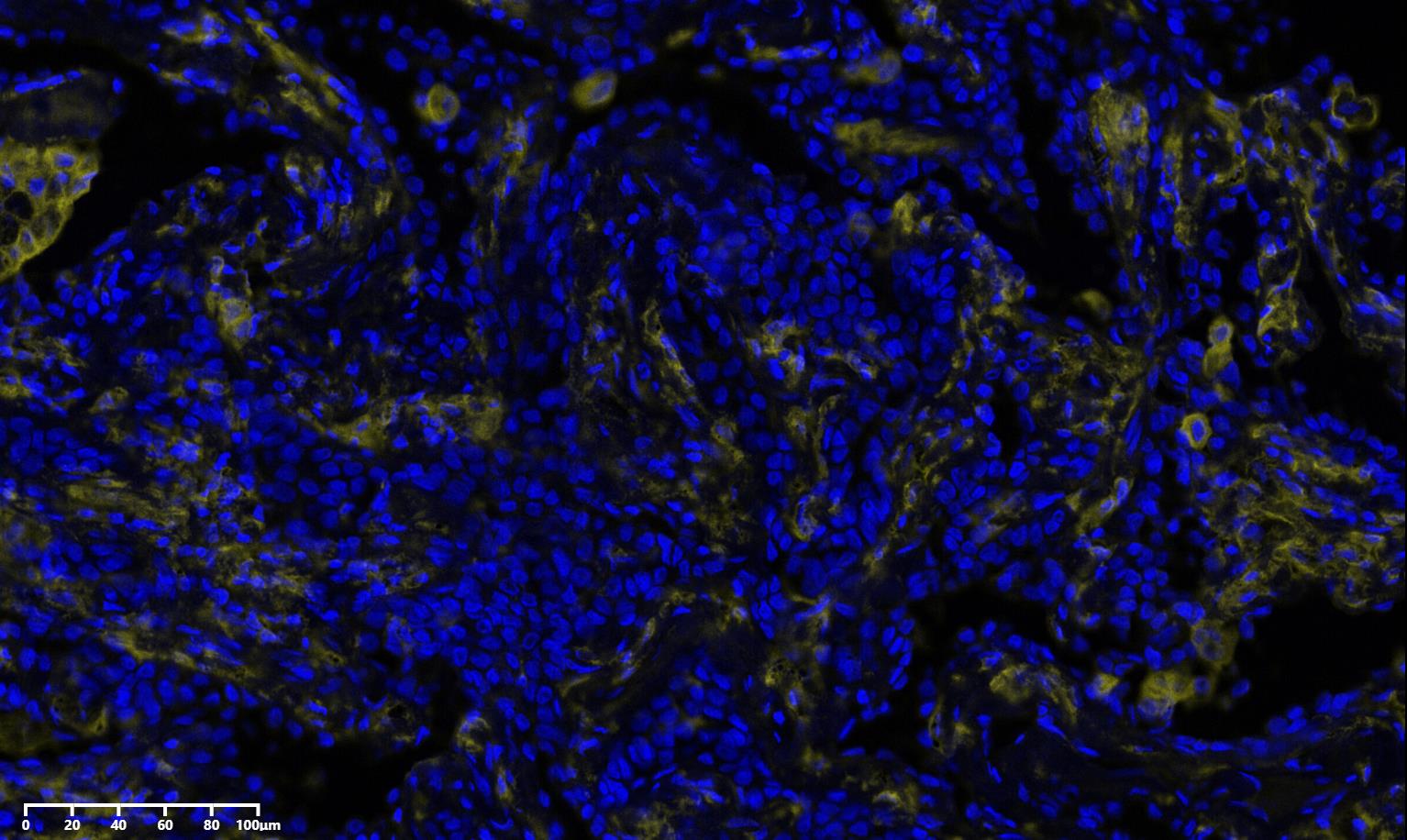

Supplement: Supplementary file 1 [file cancers-18-01384-s001.zip › original images/Microscopy/LUAD-3/IA/ITGA3.jpg]

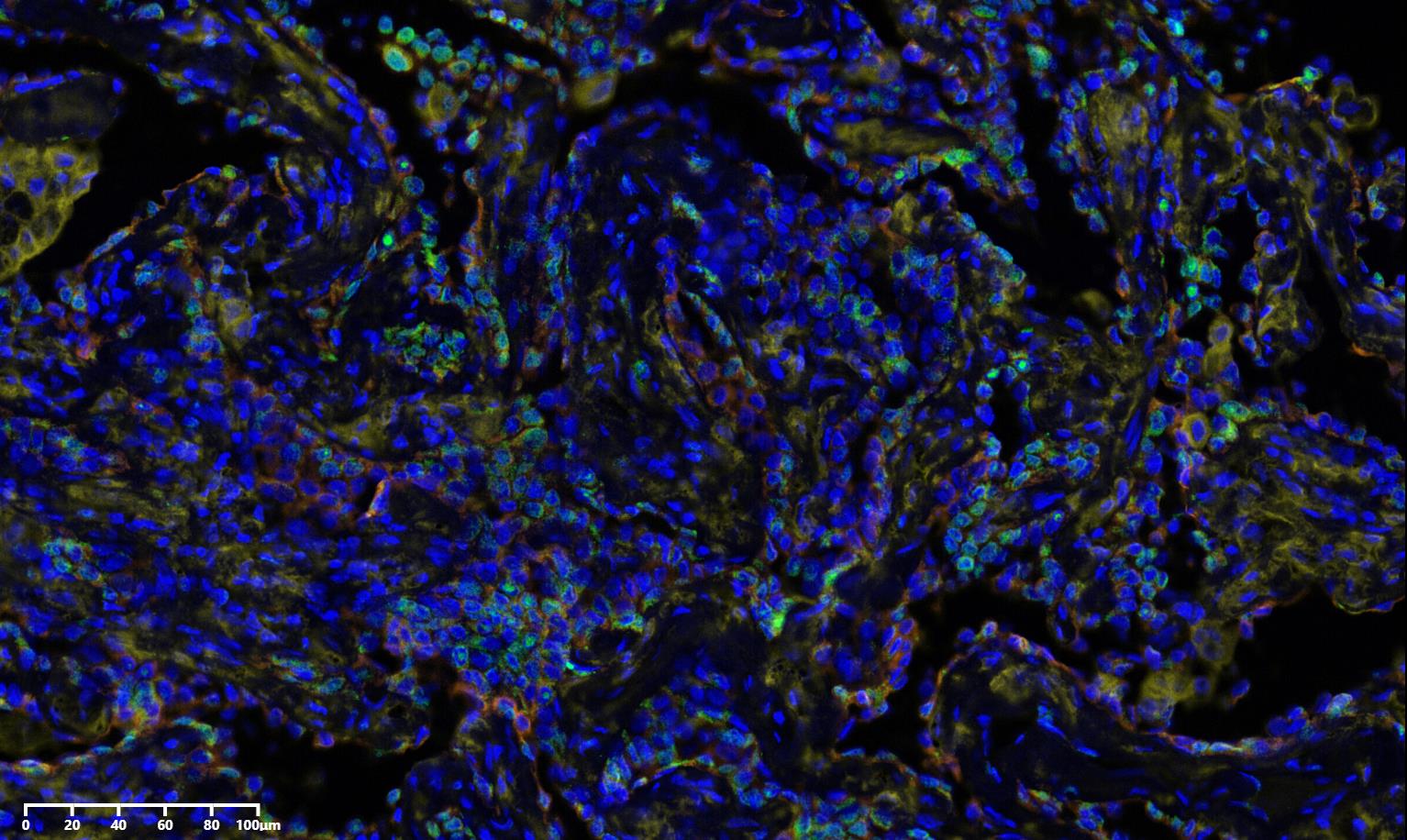

Supplement: Supplementary file 1 [file cancers-18-01384-s001.zip › original images/Microscopy/LUAD-3/IA/MERGE.jpg]

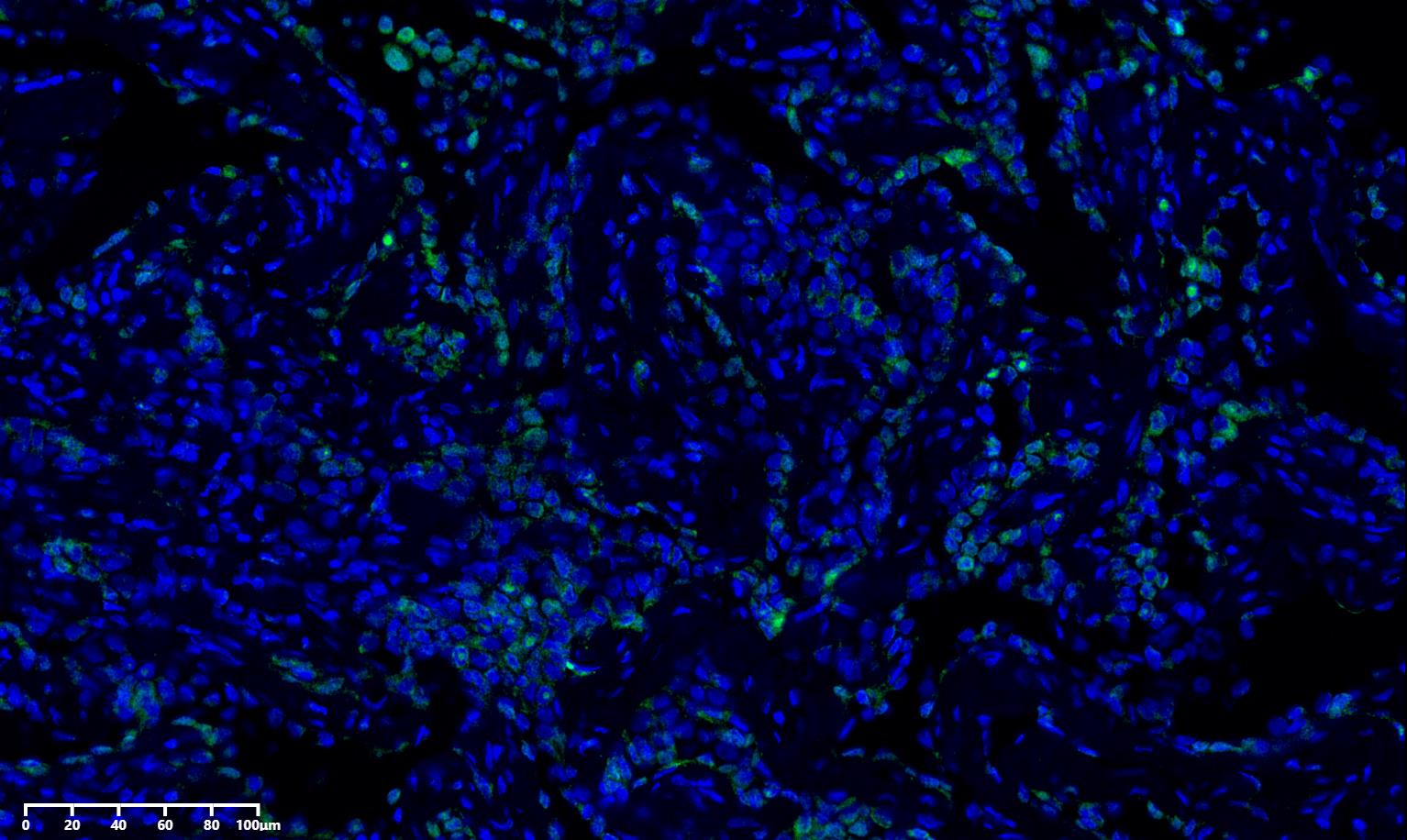

Supplement: Supplementary file 1 [file cancers-18-01384-s001.zip › original images/Microscopy/LUAD-3/IA/METTL7B.jpg]

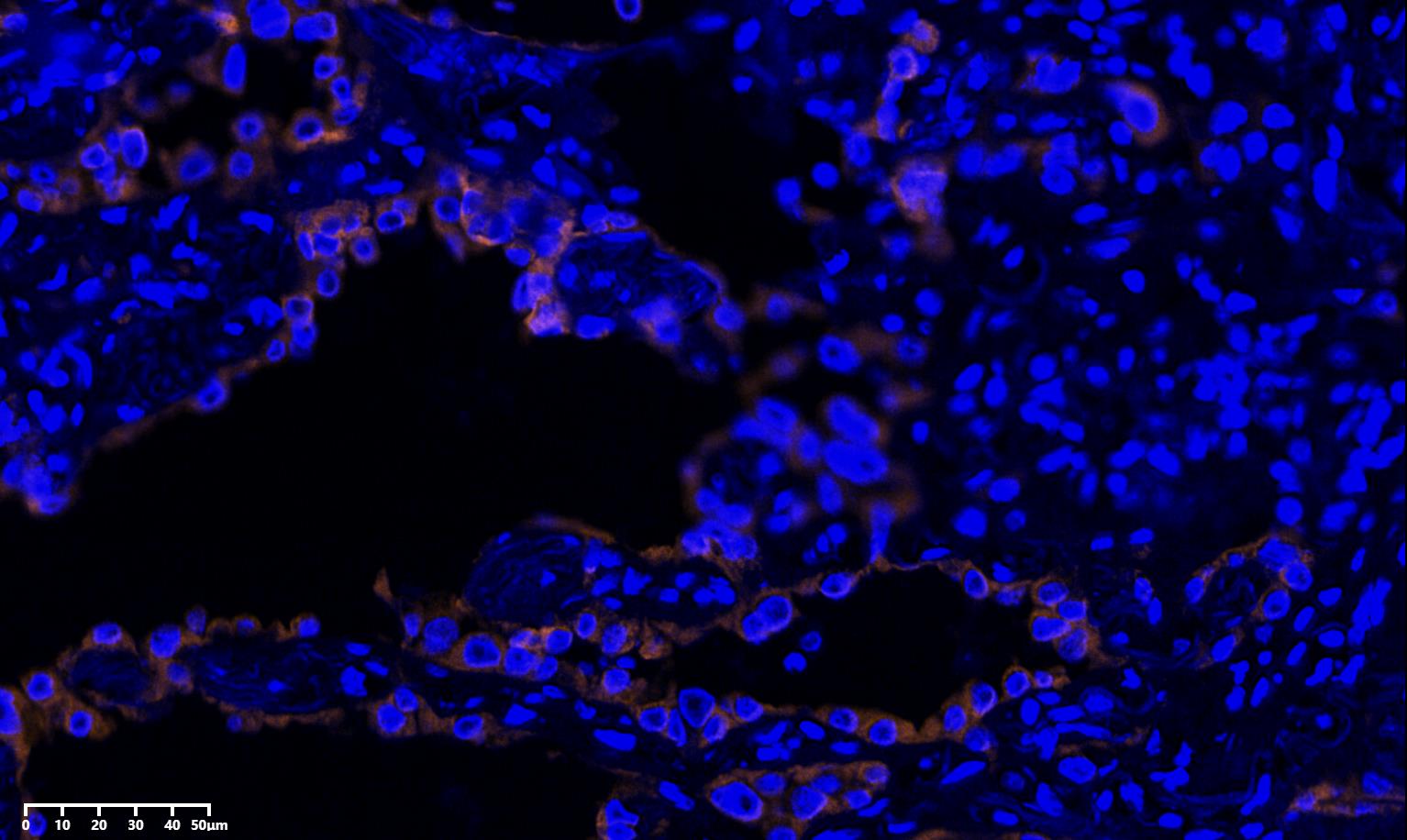

Supplement: Supplementary file 1 [file cancers-18-01384-s001.zip › original images/Microscopy/LUAD-3/MIA/EPCAM.jpg]

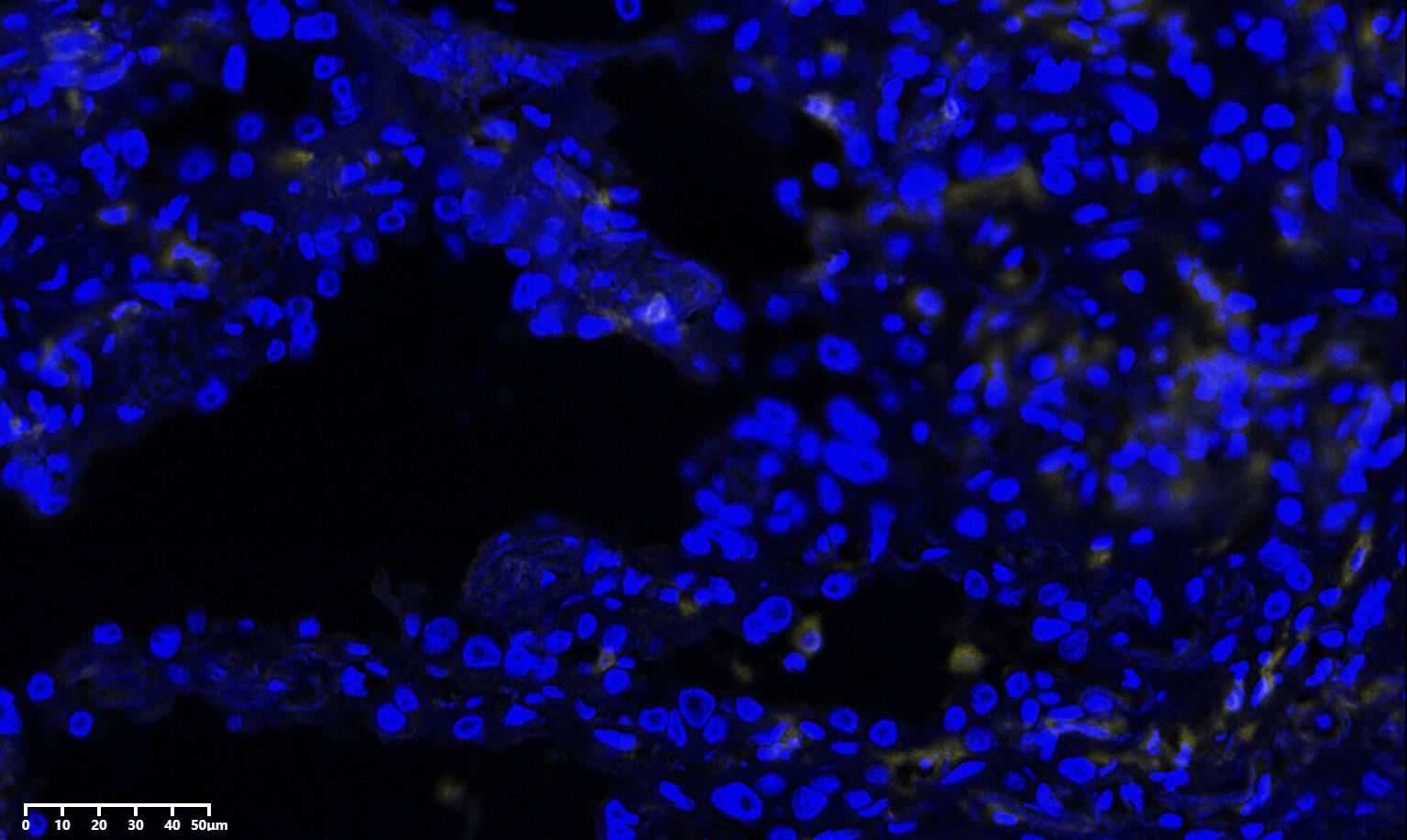

Supplement: Supplementary file 1 [file cancers-18-01384-s001.zip › original images/Microscopy/LUAD-3/MIA/ITGA3.jpg]

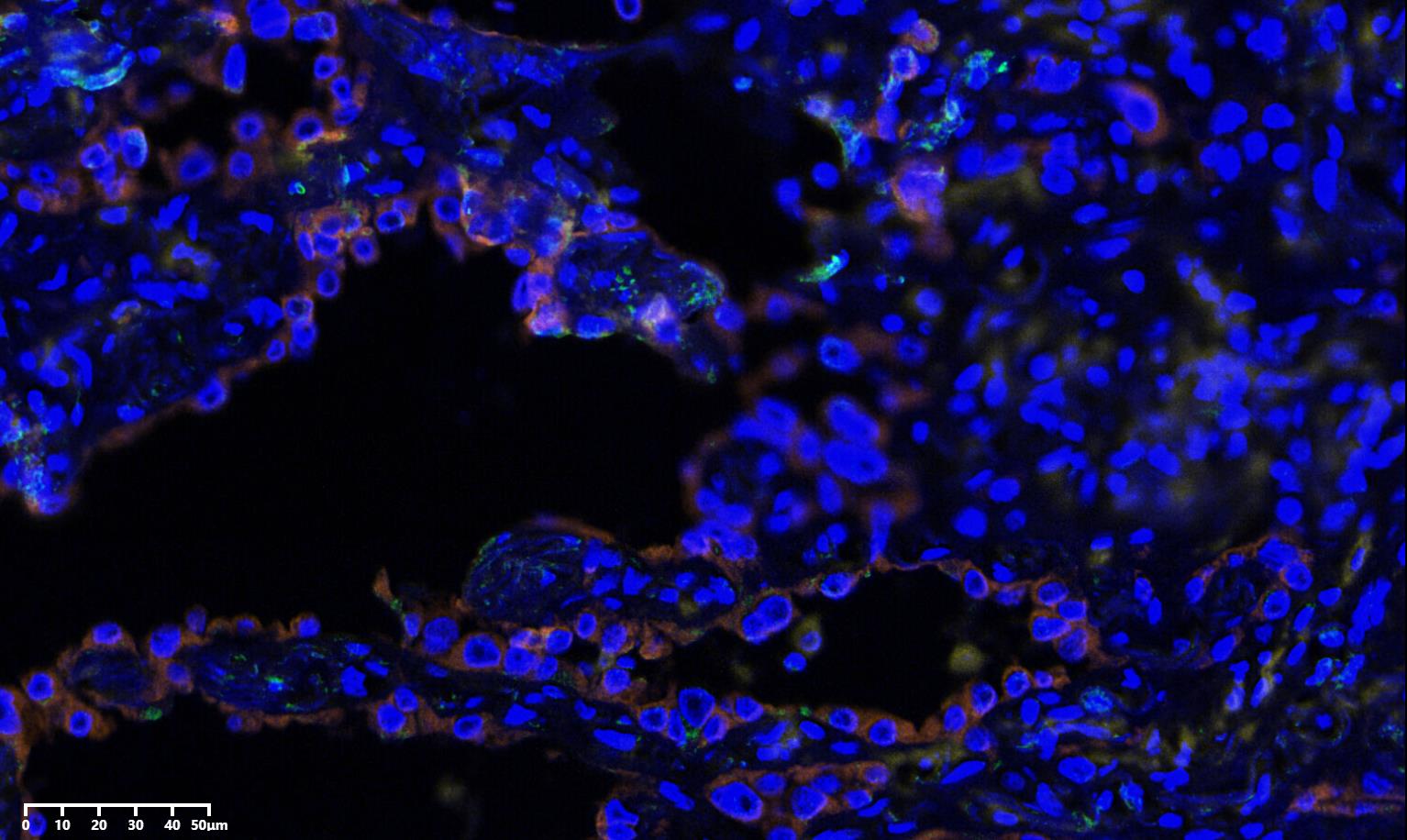

Supplement: Supplementary file 1 [file cancers-18-01384-s001.zip › original images/Microscopy/LUAD-3/MIA/MERGE.jpg]

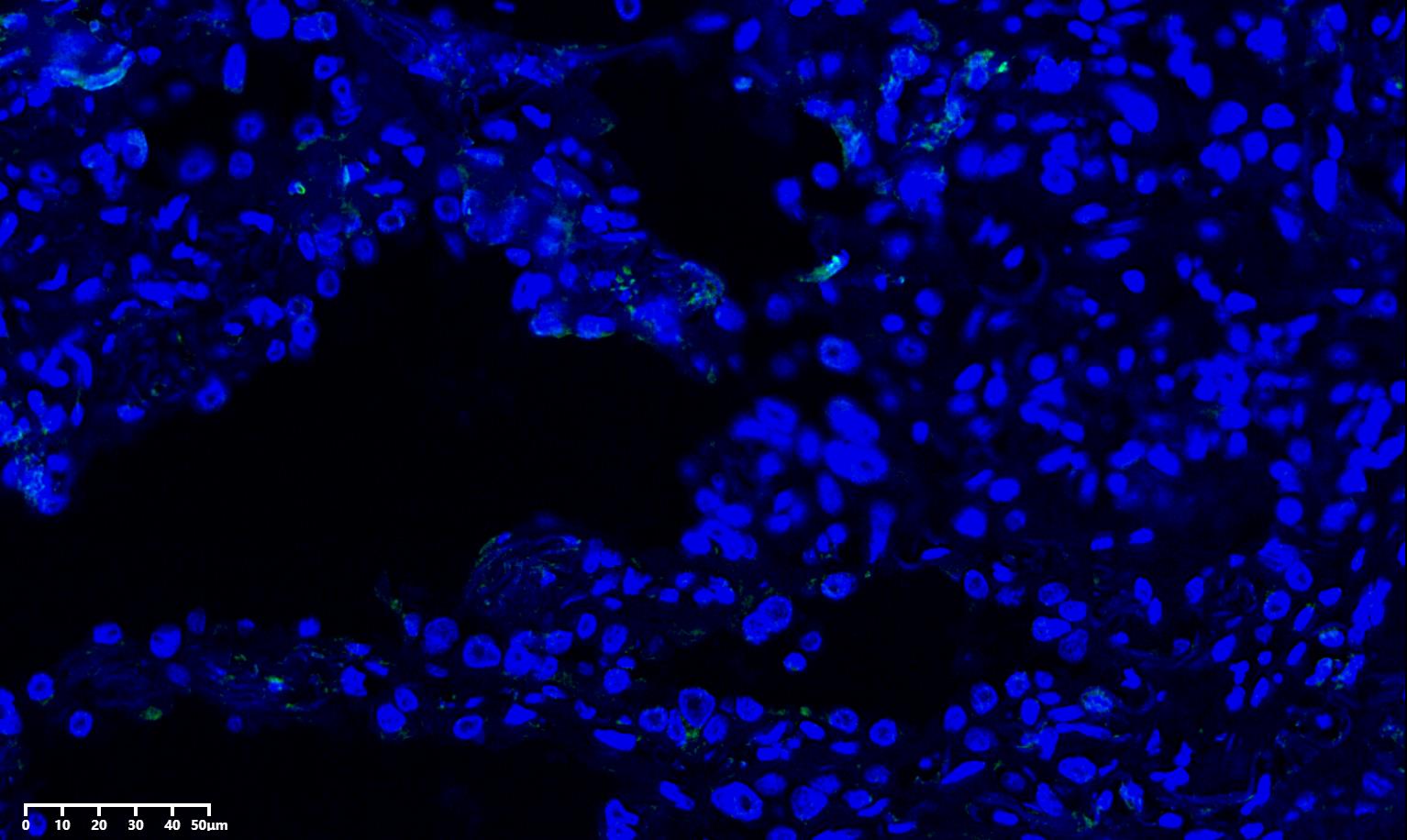

Supplement: Supplementary file 1 [file cancers-18-01384-s001.zip › original images/Microscopy/LUAD-3/MIA/METTL7B.jpg]

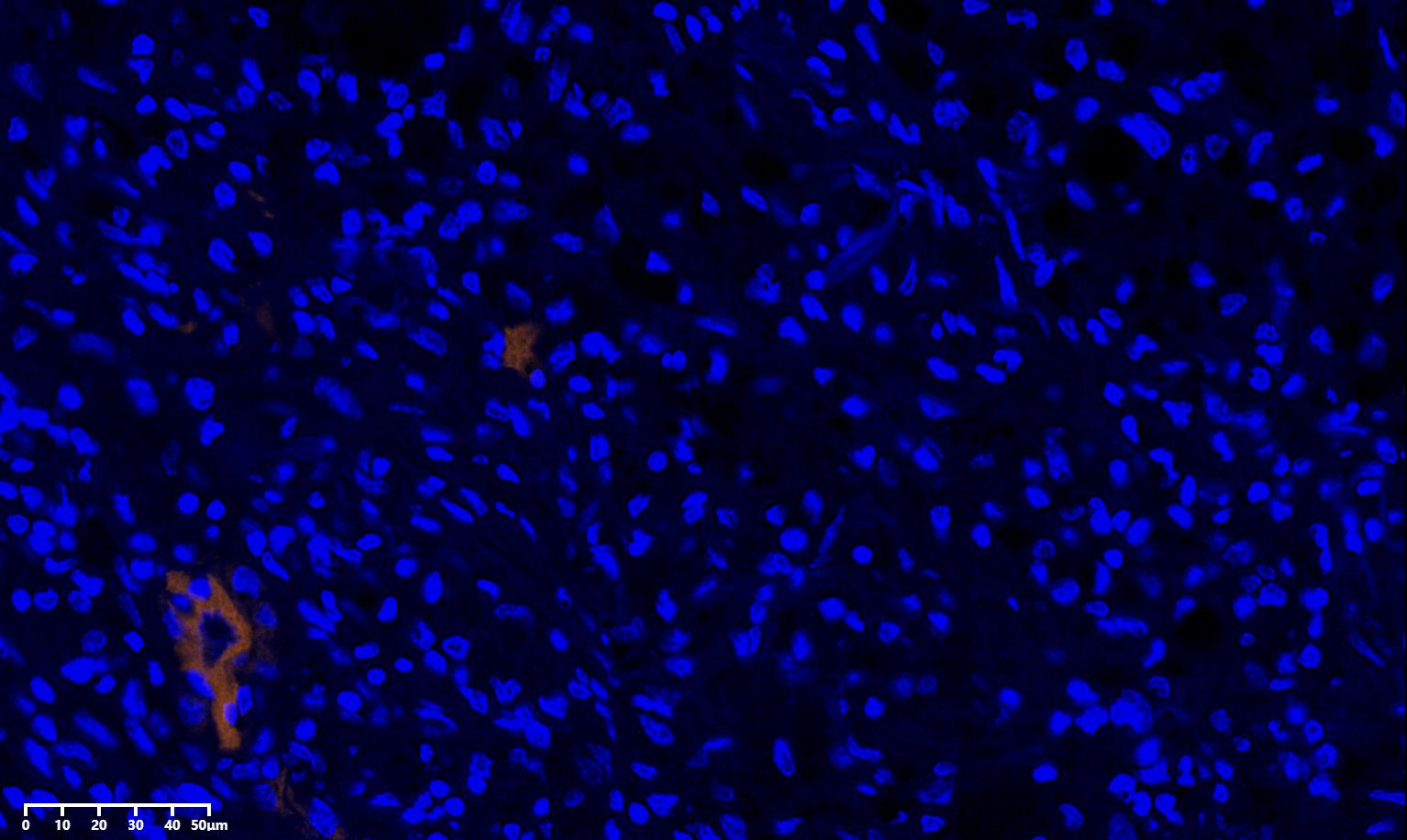

Supplement: Supplementary file 1 [file cancers-18-01384-s001.zip › original images/Microscopy/LUAD-3/paracanrous/EPCAM.jpg]

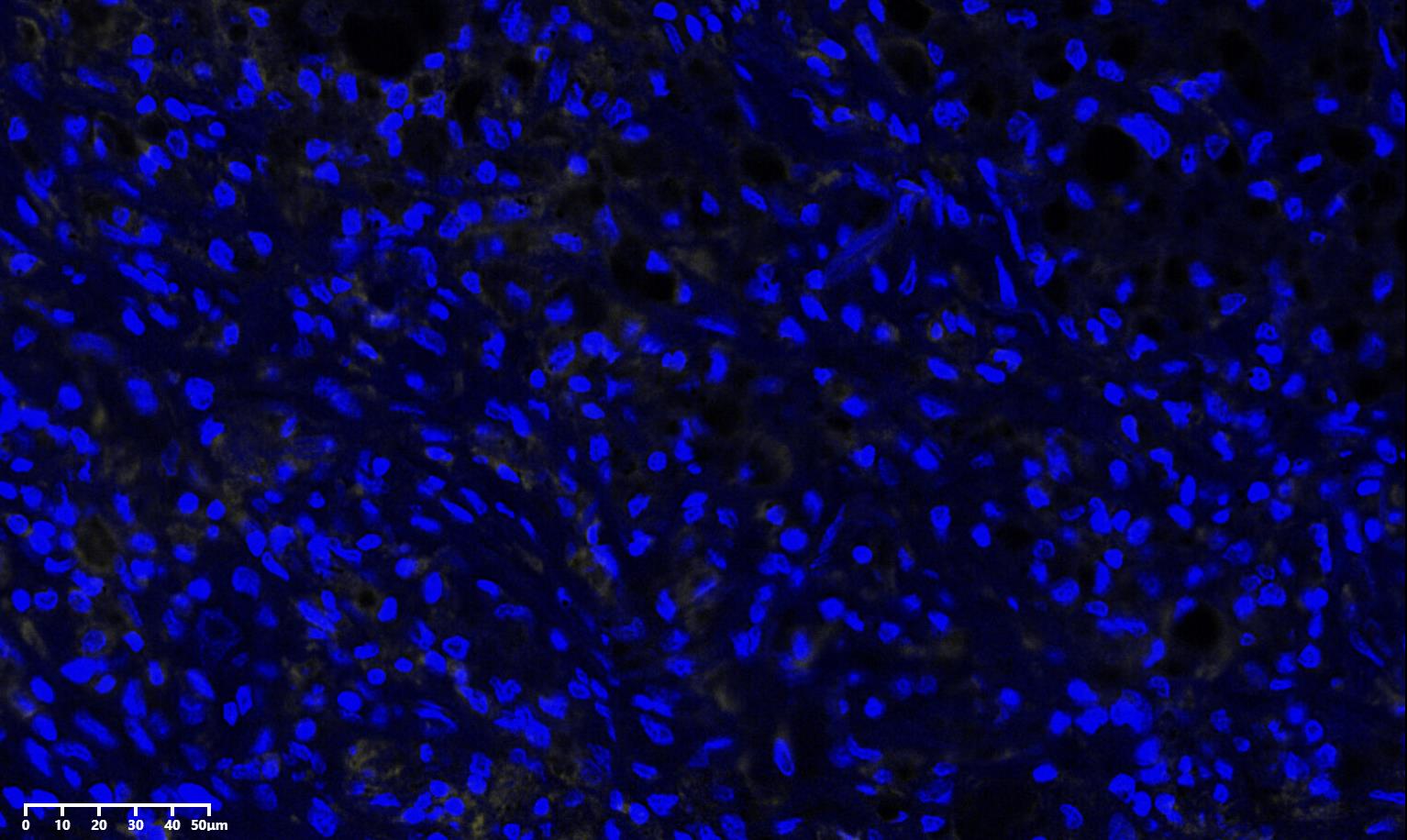

Supplement: Supplementary file 1 [file cancers-18-01384-s001.zip › original images/Microscopy/LUAD-3/paracanrous/ITGA3.jpg]

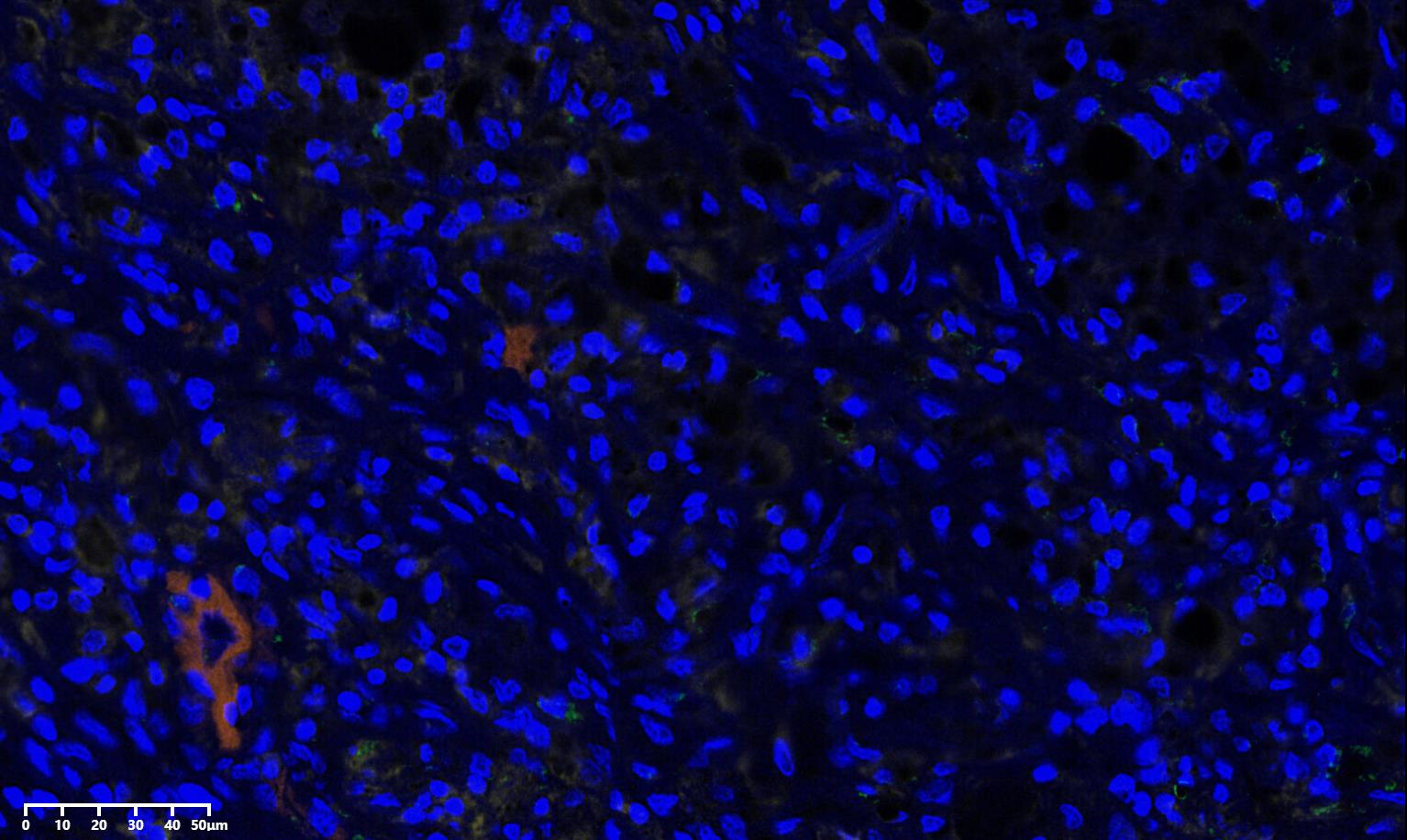

Supplement: Supplementary file 1 [file cancers-18-01384-s001.zip › original images/Microscopy/LUAD-3/paracanrous/MERGE.jpg]

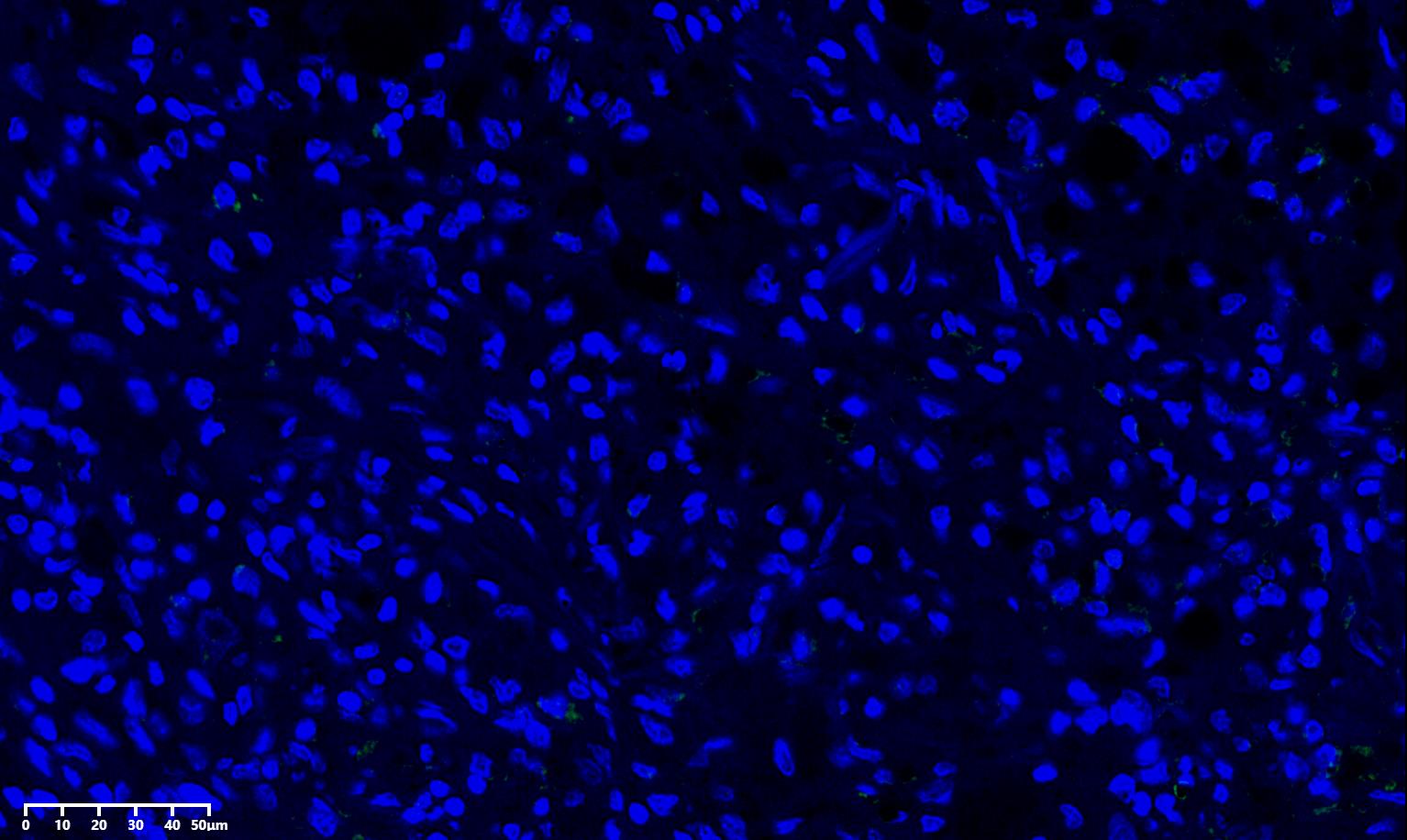

Supplement: Supplementary file 1 [file cancers-18-01384-s001.zip › original images/Microscopy/LUAD-3/paracanrous/METTL7B.jpg]
